# Highly Potent, Selective, and Competitive Indole-Based MAO-B Inhibitors Protect PC12 Cells against 6-Hydroxydopamine- and Rotenone-Induced Oxidative Stress

**DOI:** 10.3390/antiox10101641

**Published:** 2021-10-19

**Authors:** Mohamed H. Elsherbeny, Jushin Kim, Noha A. Gouda, Lizaveta Gotina, Jungsook Cho, Ae Nim Pae, Kyeong Lee, Ki Duk Park, Ahmed Elkamhawy, Eun Joo Roh

**Affiliations:** 1 Chemical Kinomics Research Center, Korea Institute of Science and Technology (KIST), Seoul 02792, Korea; mohamed.alsherbeny@pharma.asu.edu.eg; 2 Division of Bio-Medical Science & Technology, KIST School, University of Science and Technology, Seoul 02792, Korea; liz.chem.13@kist.re.kr (L.G.); anpae@kist.re.kr (A.N.P.); kdpark@kist.re.kr (K.D.P.); 3 Pharmaceutical Chemistry Department, Faculty of Pharmacy, Ahram Canadian University, Giza 12566, Egypt; 4 Convergence Research Center for Diagnosis, Treatment and Care System of Dementia, Korea Institute of Science and Technology (KIST), Seoul 02792, Korea; kimjs@kist.re.kr; 5 Department of Biotechnology, College of Life Science and Biotechnology, Yonsei University, Seoul 03722, Korea; 6 College of Pharmacy, Dongguk University-Seoul, Goyang 10326, Korea; noha.gooda@dongguk.edu (N.A.G.); kaylee@dongguk.edu (K.L.); 7 Department of Pharmaceutical Organic Chemistry, Faculty of Pharmacy, Mansoura University, Mansoura 35516, Egypt

**Keywords:** monoamine oxidase B, MAO-B inhibitors, competitive inhibitors, 6-hydroxydopamine, rotenone, oxidative stress, Parkinson’s disease, PC12 cells

## Abstract

Monoamine oxidase B (MAO-B) is responsible for dopamine metabolism and plays a key role in oxidative stress by changing the redox state of neuronal and glial cells. To date, no disease-modifying therapy for Parkinson’s disease (PD) has been identified. However, MAO-B inhibitors have emerged as a viable therapeutic strategy for PD patients. Herein, a novel series of indole-based small molecules was synthesized as new MAO-B inhibitors with the potential to counteract the induced oxidative stress in PC12 cells. At a single dose concentration of 10 µM, 10 compounds out of 30 were able to inhibit MAO-B with more than 50%. Among them, compounds **7b**, **8a**, **8b**, and **8e** showed 84.1, 99.3, 99.4, and 89.6% inhibition over MAO-B and IC_50_ values of 0.33, 0.02, 0.03, and 0.45 µM, respectively. When compared to the modest selectivity index of rasagiline (**II**, a well-known MAO-B inhibitor, SI > 50), compounds **7b**, **8a**, **8b** and **8e** showed remarkable selectivity indices (SI > 305, 3649, 3278, and 220, respectively). A further kinetic study displayed a competitive mode of action for **8a** and **8b** over MAO-B with *K*i values of 10.34 and 6.63 nM. Molecular docking studies of the enzyme-inhibitor binding complexes in MAO-B revealed that free NH and substituted indole derivatives share a common favorable binding mode: H-bonding with a crucial water “anchor” and Tyr326. Whereas in MAO-A the compounds failed to form favorable interactions, which explained their high selectivity. In addition, compounds **7b**, **8a**, **8b**, and **8e** exhibited safe neurotoxicity profiles in PC12 cells and partially reversed 6-hydroxydopamine- and rotenone-induced cell death. Accordingly, we report compounds **7b**, **8a**, **8b**, and **8e** as novel promising leads that could be further exploited for their multi-targeted role in the development of a new oxidative stress-related PD therapy.

## 1. Introduction

**Figure 1:**
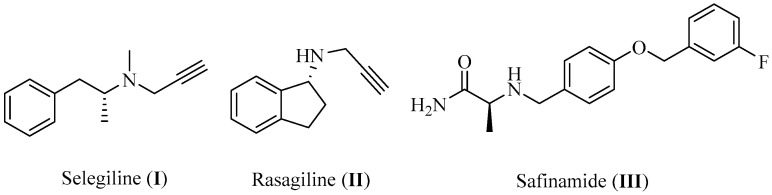
Chemical structures of current MAO-B inhibitors in the market.

Parkinson’s disease (PD), a chronic progressive condition that manifests as motor, cognitive, emotional, and autonomic impairments, is the second most prevalent neurodegenerative disease worldwide after Alzheimer’s disease (AD) [1]. As a geriatric disorder, the prevalence of PD rises sharply with aging, with incidence rates at least doubling for each 10 years increase in age [2]. Furthermore, it was found to be more predominant in males compared to females [3,4], and the prevalence was also observed to differ significantly according to ethnicity, with the least cases reported among Asians [5]. So far, there is no cure for PD, which results pathologically from the selective degeneration of the dopaminergic neurons in the Substantia nigra pars compacta [6]. Thus, the current treatments are mainly used to control the symptoms and alleviate the impairments associated with the disease through resolving the depletion of dopamine (DA) content in the patient’s midbrain. The most common treatment option for PD patients is DA replacement by administering levodopa, a precursor that is converted to DA in the brain and can be prescribed along with carbidopa to inhibit its peripheral metabolism. To avoid the complications associated with levodopa prolonged usage, other strategies for PD therapy have been developed including DA agonists which exert their pharmacological action by triggering the dopaminergic receptors directly, in addition to COMT and MAO-B inhibitors, which are both responsible for the degradation and metabolism of DA [7]. In the case of MAO-B inhibitors, certain efforts should be applied to achieve inhibition selectivity. Although MAO-B resembles its isoform MAO-A in more than 70% of its overall structure, they show differences in their substrate affinities. While MAO-A is more active towards the oxidative deamination of hydroxylated amines such as serotonin (5-HT) and norepinephrine (NE), both MAO-A and MAO-B show similar affinity to DA. Thus, an urgent need for selective MAO-B inhibitors has arisen to avoid cross inhibition of other amines. Fortunately, the structural similarity of the two isoforms still provides room for obtaining inhibitor selectivity [8]. Several MAO-B inhibitors have been introduced in the market, such as selegiline (**I**, IC_50_ = 0.007 μM), rasagiline (**II**, IC_50_ = 0.014 μM), and safinamide mesylate (**III**, IC_50_ = 0.08 μM, Figure 1) [9,10]. Although they are commonly used in the clinic as therapeutic options for PD patients, there is evidence that prolonged treatment was not effective in reversing memory deterioration in PD patients. This can be attributed to the irreversibility of **I** and **II**, which induces overexpression of MAO-B as a compensatory mechanism leading to a decline of efficacy in long-term usage [11]. Thus, vast research efforts have been directed towards the discovery of novel selective MAO-B inhibitors with a reversible mechanism of action. Several scaffolds have been investigated lately for this purpose, including chalcones, coumarins, chromones, pyrazolines, xanthines, imidazoles, indazoles, and indoles [12].

**Figure 2:**
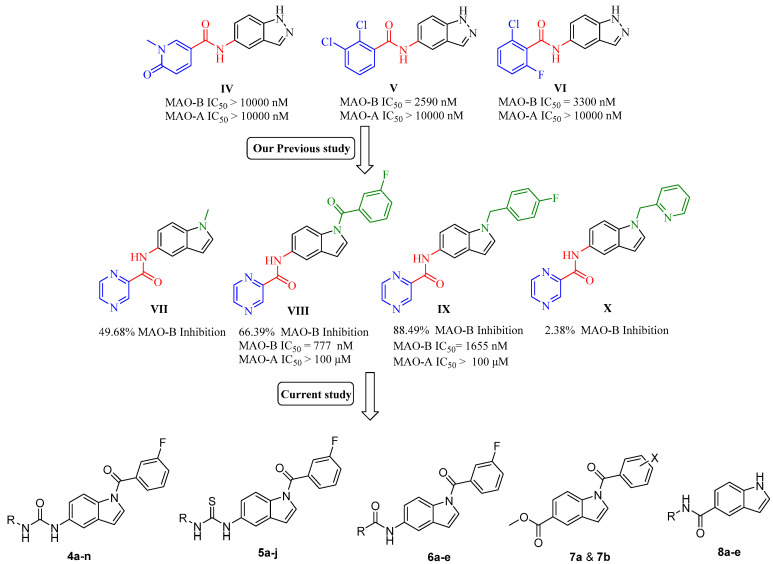
Chemical structures and MAO-B activity of compounds **IV**–**X** and the general chemical structures of the newly synthesized small molecules **4a**–**n**, **5a**–**j**, **6a**–**e**, **7a**, 7**b**, and **8a**–**e**.

The indole scaffold is considered one of the most privileged heterocyclic motifs among those attracting the attention of medicinal chemists and has become a center of research due to its unique pharmacological properties, with anticancer, anti-inflammatory, and antimicrobial applications [13,14,15]. As a continuation of our efforts to develop new small molecules as potential candidates for neurodegenerative disorders [16,17], we recently generated new indole-based MAO-B inhibitory leads [18] from previously reported indazole derivatives (**IV**, **V**, and **VI**, Figure 2) [19]. In our previous study, the indole-based compound **VIII** bearing *N*-1-(3-fluorobenzoyl) moiety was found to be the most active MAO-B inhibitor with 66.39% inhibition at a single dose concentration of 10 μM. Compound **VIII** also exhibited a far better profile in terms of potency and selectivity, with an IC_50_ value in the submicromolar range (777 nM) and selectivity index (SI > 120), which was slightly better than rasagiline **II** (SI > 50). Based on these promising results, we designed thirty novel indole-based analogs in a trial to further optimize the scaffold aiming to afford more potent derivatives, identify their mode of interaction in addition to their kinetic properties. Our design was based on keeping the N1 positioned (3-fluorobenzoyl) moiety of **VIII**, which led to a better overall inhibitory profile compared to other derivatives of the same series. Four different classes (amides, ureas, thioureas, and esters) were obtained by introducing various linkers to C5 of the indole core. The synthesized library was obtained by replacing the pyrazinyl heterocyclic ring of **VIII** with different alkyl and aryl substitutions. Furthermore, we added five more compounds based on a free NH indole scaffold while keeping the C5 amide linker. Accordingly, thirty novel compounds were obtained (**4a**–**n**, **5a**–**j**, **6a**–**e**, **7a**, 7**b**, and **8a**–**e**); among them, compounds **7b**, **8a**, **8b**, and **8e** exhibited nanomolar activity over MAO-B and successfully attenuated the induced-oxidative stress in PC12 cells. Moreover, kinetic studies and molecular docking simulations indicated a competitive and reversible mechanism of action of these new MAO-B inhibitors.

## 2. Materials and Methods

### 2.1. Chemical Reagents, Purification, and Instrumentation

All the chemicals, reagents, and solvents were purchased from commercial suppliers and used without purification, unless otherwise noted. Reactions were monitored by analytical TLC carried out using glass sheets pre-coated with silica gel 60 F_254_ purchased from Merck, with visualization under UV light (254 nm). The NMR spectra were obtained on Bruker Avance 400. Column chromatography was performed on Merck Silica Gel 60 (230–400 mesh). The high-resolution electrospray ionization mass spectrometry (HR-ESIMS) data were recorded on a JMS-700 mass spectrometer (Jeol, Japan) or by HR-ESIMS data recorded on a G2 QTOF mass spectrometer. Product purity was determined by reversed-phase high-performance liquid chromatography (RP-HPLC) using a Waters Corp. HPLC system equipped with a UV detector set at 254 nm. The mobile phases used were: (A) H_2_O containing 0.05% TFA and (B) CH_3_CN. HPLC employed a YMC Hydrosphere C18 (HS-302) column (5 μm particle size, 12 nm pore size) that was 4.6 mm in diameter × 150 mm in size with a flow rate of 1.0 mL/min. The compound purity was assessed using either a gradient of 25% B or 100% B in 30 min (method A).

### 2.2. Synthesis of (3-fluorophenyl)(5-nitro-1H-indol-1-yl)methanone (***2***)

Powdered NaOH (15 mmol, 3 eq.) was added to a solution of the starting material 5-nitroindole (**1**, 5 mmol) dissolved in 50 mL of DCM. To the stirred mixture, a small amount of tetrabutylammonium hydrogensulfate (0.03 mmol) was added and the mixture was stirred for 15 min. or till the color change to red can be noticed. Then, a 5 mL solution of 3-fluorobenzoyl chloride (1.5 eq.) in dichloromethane was added dropwise to the vigorously stirred solution. The reaction was kept stirring under nitrogen and monitored by TLC till the consumption of the starting material. The reaction mixture was then loaded on silica and purified by flash chromatography (SiO_2_, EA/n-Hex, 1:3) to obtain the target intermediate (**2**) as a white solid, yield: 72%, ^1^H NMR (400 MHz, CDCl_3_) δ 8.49 (d, J = 9.1 Hz, 2H), 8.28 (dd, J = 9.1, 2.2 Hz, 1H), 7.57–7.55 (m, 2H), 7.50–7.47 (m, 2H), 7.38–7.37 (m, 1H), 6.79 (d, J = 3.8 Hz, 1H). Reported [18].

### 2.3. Synthesis of (5-amino-1H-indol-1-yl)(3-fluorophenyl)methanone (***3***)

In a 100 mL flask, 3 mmol of the nitro group containing derivative (**2**) was dissolved in 40 mL of EtOAc, followed by adding 150 mg of Pt/C catalyst powder under N_2_. The mixture was stirred under hydrogen gas for 75 min at room temperature. The mixture was then filtered on a celite pad to remove the metal catalyst, and the solvent was removed in vacuo. The product was purified by silica gel chromatography (SiO_2_, CH_2_Cl_2_/EtOAc, 9:1) to yield the amine derivative (**3**) as a yellow oil that solidifies on cooling, yield: 79%, ^1^H NMR (400 MHz, CDCl_3_) δ 8.19 (d, J = 8.7 Hz, 1H), 7.49–7.47 (m, 2H), 7.40 (dd, J = 9.2, 2.5 Hz, 1H)), 7.30–7.27 (m, 1H), 7.15 (d, J = 3.7 Hz, 1H), 6.86 (d, J = 2.2 Hz, 1H), 6.76 (dd, J = 8.7, 2.3 Hz, 1H), 6.46 (d, J = 3.7 Hz, 1H), 3.70 (s, 2H). Reported [18].

### 2.4. General Procedure of Urea Derivatives (***4a***–***n***)

To a dry 25 mL round bottom flask containing a solution of the pre-final amine (**3**, 0.5 mmol) and DIPEA (0.6 mmol) dissolved in 10 mL of DCM, the appropriate isocyanate reagent (0.5 mmol) was added, and the reaction mixture was stirred overnight at room temperature to yield the desired urea derivative. The target compound was either purified by filtration followed by washing using DCM, or by flash column chromatography (SiO2, DCM:EA, 9:1) if required.

#### 2.4.1. 1-Ethyl-3-(1-(3-fluorobenzoyl)-1*H*-indol-5-yl)urea (**4a**)

White solid, yield: 83%, mp: 226.7–227.7 °C, ^1^H NMR (400 MHz, DMSO-d_6_): δ 8.51 (s, 1H), 8.13 (d, J = 8.8 Hz, 1H), 7.84 (d, J = 1.8 Hz, 1H), 7.66–7.51 (m, 4H), 7.31 (d, J = 3.7 Hz, 1H), 7.25 (dd, J = 8.8, 1.9 Hz, 1H), 6.69 (d, J = 3.7 Hz, 1H), 6.10 (t, J = 5.5 Hz, 1H), 3.13 (p, J = 7.1 Hz, 2H), 1.08 (t, J = 7.2 Hz, 3H). ^13^C NMR (100 MHz, DMSO-d_6_) δ 166.92, 163.42, 160.97, 155.79, 137.64, 136.85, 131.62, 131.40, 130.77, 128.80, 125.42, 119.27, 119.06, 116.40, 116.13, 109.74, 34.45, 15.99.

#### 2.4.2. Ethyl ((1-(3-fluorobenzoyl)-1*H*-indol-5-yl)carbamoyl)glycinate (**4b**)

White solid, yield: 26%, mp: 203.3–204.3 °C, HPLC purity: 14.15 min, 95.70%, ^1^H NMR (400 MHz, DMSO-d_6_): δ 8.90 (s, 1H), 8.16 (d, J = 8.8 Hz, 1H), 7.85 (d, J = 1.4 Hz, 1H), 7.66–7.58 (m, 3H), 7.54 (t, J = 8.2 Hz, 1H), 7.33 (d, J = 3.7 Hz, 1H), 7.28 (dd, J = 8.9, 1.6 Hz, 1H), 6.70 (d, J = 3.7 Hz, 1H), 6.47 (t, J = 5.8 Hz, 1H), 4.13 (q, J = 7.1 Hz, 2H), 3.90 (d, J = 5.8 Hz, 2H), 1.23 (t, J = 7.1 Hz, 3H). ^13^C NMR (100 MHz, DMSO-d_6_) δ 171.38, 166.96, 163.41, 160.97, 155.83, 137.24, 136.82, 131.63, 131.41, 130.97, 128.91, 125.44, 119.08, 116.47, 116.34, 116.14, 109.91, 60.83, 41.89, 14.61. HRMS (ESI) *m*/*z* calculated for C_20_H_18_FN_3_O_4_ [M + H]^+^: 384.1360. Found: 384.1349.

#### 2.4.3. 1-(2-Chloroethyl)-3-(1-(3-fluorobenzoyl)-1*H*-indol-5-yl)urea (**4c**)

Brown solid, yield: 74%, mp: 191.3–192.3 °C, ^1^H NMR (400 MHz, DMSO-d_6_): δ 8.77 (d, J = 8.5 Hz, 1H), 8.17 (t, J = 8.8 Hz, 1H), 7.86 (d, J = 8.8 Hz, 1H), 7.68–7.49 (m, 4H), 7.37–7.26 (m, 2H), 6.71 (d, J = 5.8 Hz, 1H), 6.44 (s, 1H), 3.74–3.66 (m, 2H), 3.51–3.42 (m, 2H). ^13^C NMR (100 MHz, DMSO-d_6_) δ 166.93, 163.41, 160.97, 155.71, 153.40, 137.31, 136.80, 131.62, 131.38, 130.94, 128.85, 125.41, 119.27, 116.36, 116.12, 109.56, 44.92, 41.73. HRMS (ESI) *m*/*z* calculated for C_18_H_15_ClFN_3_O_2_ [M + H]^+^: 360.0915. Found: 360.0905.

#### 2.4.4. 1-Cyclohexyl-3-(1-(3-fluorobenzoyl)-1*H*-indol-5-yl)urea (**4d**)

White solid, yield: 79%, mp: 222.7–223.7 °C, ^1^H NMR (400 MHz, DMSO-d_6_): δ 8.40 (s, 1H), 8.14 (d, J = 8.8 Hz, 1H), 7.83 (d, J = 1.7 Hz, 1H), 7.68–7.50 (m, 4H), 7.31 (d, J = 3.7 Hz, 1H), 7.24 (dd, J = 8.9, 1.8 Hz, 1H), 6.69 (d, J = 3.6 Hz, 1H), 6.07 (d, J = 7.8 Hz, 1H), 3.50 (t, J = 3.8 Hz, 1H), 1.89–1.78 (m, 2H), 1.71–1.64 (m, 2H), 1.58–1.52 (m, 1H), 1.36–1.27 (m, 2H), 1.23–1.16 (m, 2H). ^13^C NMR (100 MHz, DMSO-d_6_) δ 166.89, 163.42, 160.98, 155.07, 137.62, 136.85, 131.64, 131.48, 131.40, 130.76, 128.79, 125.41, 119.05, 116.43, 116.17, 109.59, 48.09, 33.49, 25.74, 24.84.

#### 2.4.5. 1-(1-(3-Fluorobenzoyl)-1*H*-indol-5-yl)-3-(4-nitrophenyl)urea (**4e**)

White solid, yield: 88%, mp: 249.1–250.1 °C, ^1^H NMR (400 MHz, DMSO-d_6_): δ 9.46 (s, 1H), 9.04 (s, 1H), 8.22 (t, J = 9.5 Hz, 3H), 7.93 (s, 1H), 7.71 (d, J = 9.1 Hz, 2H), 7.65–7.61 (m, 3H), 7.54 (t, J = 7.8 Hz, 1H), 7.38 (d, J = 4.1 Hz, 2H), 6.76 (d, J = 3.6 Hz, 1H). ^13^C NMR (100 MHz, DMSO-d_6_) δ 167.05, 163.43, 160.99, 152.59, 146.95, 141.42, 136.65, 135.86, 131.68, 131.43, 129.20, 125.64, 125.50, 119.19, 117.91, 117.15, 116.60, 116.20, 111.05, 109.53.

#### 2.4.6. 1-(1-(3-Fluorobenzoyl)-1*H*-indol-5-yl)-3-(4-methoxyphenyl)urea (**4f**)

White solid, yield: 81%, mp: 250.0–251.0 °C, ^1^H NMR (400 MHz, DMSO-d_6_): δ 8.69 (s, 1H), 8.49 (s, 1H), 8.19 (d, J = 8.8 Hz, 1H), 7.91 (d, J = 1.5 Hz, 1H), 7.68–7.50 (m, 4H), 7.44–7.31 (m, 4H), 6.88 (d, J = 8.9 Hz, 2H), 6.73 (d, J = 3.6 Hz, 1H), 3.73 (s, 3H). ^13^C NMR (100 MHz, DMSO-d_6_) δ 166.99, 163.42, 160.98, 154.91, 153.38, 136.86, 136.72, 133.27, 131.67, 131.42, 131.18, 128.99, 125.46, 120.48, 119.12, 116.70, 116.17, 114.47, 110.33, 109.58, 55.64. HRMS (ESI) *m*/*z* calculated for C_23_H_18_FN_3_O_3_ [M + H]^+^: 404.1410. Found: 404.1407.

#### 2.4.7. 1-(3,5-Dimethoxyphenyl)-3-(1-(3-fluorobenzoyl)-1*H*-indol-5-yl)urea (**4g**)

White solid, yield: 85%, mp: 235.6–236.6 °C, ^1^H NMR (400 MHz, DMSO-d_6_): δ 8.76 (s, 1H), 8.70 (s, 1H), 8.20 (d, J = 8.8 Hz, 1H), 7.92 (d, J = 1.8 Hz, 1H), 7.67–7.59 (m, 3H), 7.65–7.61 (m, 3H), 7.54 (t, J = 6.9 Hz, 1H), 7.37 (d, J = 3.7 Hz, 1H), 7.33 (dd, J = 8.9, 1.9 Hz, 1H), 6.75 (d, J = 3.7 Hz, 1H), 6.72 (d, J = 2.1 Hz, 2H), 6.16 (t, J = 2.0 Hz, 1H), 3.74 (s, 6H). ^13^C NMR (100 MHz, DMSO-d_6_) δ 166.99, 163.42, 161.10, 160.98, 153.04, 141.98, 136.76, 136.49, 131.68, 131.50, 131.33, 129.04, 125.47, 119.35, 116.83, 116.17, 110.55, 109.58, 96.87, 94.40, 55.50.

#### 2.4.8. 1-(3,5-Dichlorophenyl)-3-(1-(3-fluorobenzoyl)-1*H*-indol-5-yl)urea (**4h**)

White solid, yield: 87%, ^1^H NMR (400 MHz, DMSO-d_6_): δ 9.06 (s, 1H), 8.98 (s, 1H), 8.21 (d, J = 8.8 Hz, 1H), 7.91 (s, 1H), 7.64–7.57 (m, 6H), 7.38 (d, J = 4 Hz, 2H), 7.15 (s, 1H), 6.74 (d, J = 3.6 Hz, 1H). ^13^C NMR (100 MHz, DMSO-d_6_) δ 167.02, 163.42, 160.98, 152.86, 142.84, 136.64, 136.01, 134.56, 131.63, 131.49, 129.10, 125.48, 121.26, 119.37, 117.16, 116.75, 116.54, 116.19, 111.06, 109.51.

#### 2.4.9. 1-(1-(3-Fluorobenzoyl)-1*H*-indol-5-yl)-3-(4-fluorophenyl)urea (**4i**)

White solid, yield: 75%, mp: 264.3–265.3 °C, ^1^H NMR (400 MHz, DMSO-d_6_): δ 8.77 (s, 1H), 8.72 (s, 1H), 8.20 (d, J = 8.8 Hz, 1H), 7.91 (s, 1H), 7.69–7.47 (m, 6H), 7.38–7.33 (m, 2H), 7.14 (t, J = 8.8 Hz, 2H), 6.74 (d, J = 3.6 Hz, 1H). ^13^C NMR (100 MHz, DMSO-d_6_) δ 166.97, 163.43, 160.99, 158.98, 156.61, 153.28, 136.77, 136.61, 131.66, 131.32, 129.02, 125.43, 120.38, 119.13, 116.85, 116.17, 115.85, 115.63, 110.55, 109.55.

#### 2.4.10. 1-(3,4-Dichlorophenyl)-3-(1-(3-fluorobenzoyl)-1*H*-indol-5-yl)urea (**4j**)

White solid, yield: 77%, mp: 247.8–248.8 °C, ^1^H NMR (400 MHz, DMSO-d_6_): δ 9.00 (s, 1H), 8.91 (s, 1H), 8.21 (d, J = 8.8, 1H), 7.91 (dd, J = 7.0, 1.9 Hz, 2H), 7.67–7.51 (m, 5H), 7.38–7.35 (m, 3H), 6.74 (d, J = 3.7 Hz, 1H). ^13^C NMR (100 MHz, DMSO-d_6_) δ 166.98, 163.43, 160.98, 152.95, 140.54, 136.74, 136.15, 131.64, 131.52, 131.41, 131.02, 129.09, 125.47, 123.49, 119.76, 118.79, 117.07, 116.54, 116.42, 116.18, 110.92, 109.52.

#### 2.4.11. 1-(4-Chloro-3-(trifluoromethyl)phenyl)-3-(1-(3-fluorobenzoyl)-1*H*-indol-5-yl)urea (**4k**)

White solid, yield: 84%, mp: 210.9–211.9 °C, ^1^H NMR (400 MHz, DMSO-d_6_): δ 9.28 (s, 1H), 9.03 (s, 1H), 8.24–8.21 (m, 1H), 8.17 (s, 1H), 7.93 (s, 1H), 7.65–7.54 (m, 6H), 7.38 (s, 2H), 6.75 (s, 1H). ^13^C NMR (100 MHz, DMSO-d_6_) δ 167.00, 163.42, 160.98, 153.06, 139.96, 136.75, 136.08, 132.46, 131.65, 131.50, 129.11, 127.03, 125.44, 123.43, 122.64, 119.36, 119.15, 117.11, 116.54, 116.18, 111.00, 109.53.

#### 2.4.12. 1-(1-(3-Fluorobenzoyl)-1*H*-indol-5-yl)-3-(4-fluorophenethyl)urea (**4l**)

White solid, yield: 76%, mp: 231.5–232.5 °C, HPLC purity: 18.28 min, 95.50%, ^1^H NMR (400 MHz, DMSO-d_6_): δ 8.58 (s, 1H), 8.14 (d, J = 8.76 Hz, 1H), 7.86 (s, 1H), 7.68–7.50 (m, 4H), 7.35–7.23 (m, 4H), 7.15 (t, J = 8.5 Hz, 2H), 6.69 (d, J = 2.9 Hz, 1H), 6.14 (t, J = 5.1 Hz, 1H), 3.37 (s, 2H), 2.78 (t, J = 7.3 Hz, 2H). ^13^C NMR (100 MHz, DMSO-d_6_) δ 166.93, 163.42, 162.52, 160.98, 160.12, 155.80, 137.54, 136.84, 136.21, 131.63, 131.40, 130.97, 130.82, 128.83, 125.42, 119.28, 116.43, 116.28, 115.61, 109.60, 41.15, 35.47. HRMS (ESI) *m*/*z* calculated for C_24_H_19_F_2_N_3_O_2_ [M + H]^+^: 420.1524. Found: 420.1517.

#### 2.4.13. 1-(1-(3-Fluorobenzoyl)-1*H*-indol-5-yl)-3-(4-phenoxyphenyl)urea (**4m**)

White solid, yield: 71%, mp: 235.2–236.2 °C, ^1^H NMR (400 MHz, DMSO-d_6_): δ 8.77 (s, 1H), 8.71 (s, 1H), 8.21 (d, J = 8.8 Hz, 1H), 7.93 (d, J = 1.6 Hz, 1H), 7.70–7.48 (m, 6H), 7.42–7.33 (m, 4H), 7.10 (t, J = 7.4 Hz, 1H), 7.04–6.95 (m, 4H), 6.74 (d, J = 3.6 Hz, 1H). ^13^C NMR (100 MHz, DMSO-d_6_) δ 167.00, 163.43, 160.98, 158.15, 153.27, 151.10, 136.68, 136.27, 131.67, 131.42, 131.28, 130.40, 129.03, 125.47, 123.24, 120.38, 120.26, 119.35, 119.14, 118.10, 116.79, 116.18, 110.47, 109.57.

#### 2.4.14. 1-(1-(3-Fluorobenzoyl)-1*H*-indol-5-yl)-3-(4-morpholinophenyl)urea (**4n**)

White solid, yield: 62%, mp: 271.1–272.1 °C, ^1^H NMR (400 MHz, DMSO-d_6_): δ 8.69 (s, 1H), 8.44 (s, 1H), 8.19 (d, J = 8.8 Hz, 1H), 7.91 (d, J = 1.8 Hz, 1H), 7.70–7.50 (m, 4H), 7.40–7.30 (m, 4H), 6.90 (d, J = 8.9 Hz, 2H), 6.73 (d, J = 3.7 Hz, 1H), 3.75 (t, J = 4.9 Hz, 4H), 3.04 (t, J = 4.7 Hz, 4H). ^13^C NMR (100 MHz, DMSO-d_6_) δ 167.05, 163.68, 161.05, 153.35, 146.92, 136.91, 132.56, 131.67, 131.51, 131.43, 131.14, 128.98, 125.46, 120.08, 119.35, 116.65, 116.52, 116.16, 110.26, 109.59, 66.64, 49.73.

### 2.5. General Procedure of Thiourea Derivatives (***5a***–***j***)

A mixture of the pre-final amine **3** (0.5 mmol) and the appropriate isothiocyanate derivative (0.5 mmol) was dissolved in acetonitrile (5 mL) and stirred for 6 h at 80 °C to yield the corresponding thiourea derivative. After the completion of the reaction, acetonitrile was removed in vacuo, and the final compound was purified using the same general procedure for derivatives **4a**–**n**.

#### 2.5.1. 1-Ethoxycarbonyl-3-(1-(3-fluorobenzoyl)-1*H*-indol-5-yl)thiourea (**5a**)

White solid, yield: 47%, mp: 199.4–200.4 °C, ^1^H NMR (400 MHz, CDCl_3_): δ 11.51 (s, 1H), 8.44–8.36 (m, 1H), 8.11 (s, 1H), 8.01 (s, 1H), 7.50 (d, J = 3.4 Hz, 2H), 7.44 (s, 2H), 7.35–7.23 (m, 2H), 6.64 (d, J = 3.9 Hz, 1H), 4.35–4.25 (m, 2H), 1.36 (t, J = 5.9 Hz, 3H). ^13^C NMR (100 MHz, CDCl_3_) δ 178.32, 163.72, 152.83, 134.39, 133.93, 131.07, 130.53, 128.21, 124.84, 122.13, 119.26, 119.05, 117.06, 116.73, 116.46, 116.23, 109.21, 63.10, 14.23.

#### 2.5.2. 1-(2-Chloroethyl)-3-(1-(3-fluorobenzoyl)-1*H*-indol-5-yl)thiourea (**5b**)

White solid, yield: 68%, mp: 221.5–222.5 °C, ^1^H NMR (400 MHz, DMSO-d_6_): δ 8.35 (d, J = 8.0 Hz, 1H), 7.72–7.54 (m, 7H), 7.37 (d, J = 7.6 Hz, 1H), 6.84 (s, 1H), 5.77 (s, 1H), 3.98 (s, 2H), 3.61 (s, 2H). ^13^C NMR (100 MHz, DMSO-d_6_) δ 167.33, 163.42, 160.98, 136.19, 134.68, 131.87, 131.54, 130.34, 125.70, 121.49, 119.76, 117.28, 116.62, 116.38, 109.20, 49.81, 31.34.

#### 2.5.3. 1-(1-(3-Fluorobenzoyl)-1*H*-indol-5-yl)-3-(4-morpholinophenyl)thiourea (**5c**)

White solid, yield: 66%, mp: 192.7–193.7 °C, ^1^H NMR (400 MHz, DMSO-d_6_): δ 9.67 (s, 1H), 9.56 (s, 1H), 8.22 (d, J = 8.8 Hz, 1H), 7.78–7.53 (m, 4H), 7.43–7.38 (m, 2H), 7.31 (d, J = 8.9 Hz, 2H), 6.92 (d, J = 9.0 Hz, 2H), 6.77 (d, J = 3.7 Hz, 1H), 3.75 (t, J = 4.8 Hz, 4H), 3.10 (t, J = 4.8 Hz, 4H). ^13^C NMR (100 MHz, DMSO-d_6_) δ 180.46, 167.16, 163.44, 160.99, 148.91, 136.63, 136.25, 132.99, 131.55, 131.19, 129.13, 125.92, 125.54, 122.38, 119.50, 117.06, 116.48, 116.06, 115.50, 109.49, 66.59, 49.15. HRMS (ESI) *m*/*z* calculated for C_26_H_23_FN_4_O_2_S [M + H]^+^: 475.1604. Found: 475.1602.

#### 2.5.4. 1-(1-(3-Fluorobenzoyl)-1*H*-indol-5-yl)-3-(2-morpholinoethyl)thiourea (**5d**)

White solid, yield: 59%, mp: 179.9–180.9 °C, ^1^H NMR (400 MHz, CDCl_3_): δ 8.44 (d, J = 8.8 Hz, 1H), 8.05 (s, 1H), 7.55–7.45 (m, 4H), 7.36 (d, J = 2.4 Hz, 2H), 7.23 (d, J = 8.8 Hz, 1H), 7.01 (s, 1H), 6.63 (d, J = 3.2 Hz, 1H), 3.69 (s, 2H), 3.46 (s, 4H), 2.53 (s, 2H), 2.37 (s, 4H). ^13^C NMR (100 MHz, CDCl_3_) δ 167.00, 163.74, 161.26, 135.94, 134.50, 131.85, 130.64, 130.56, 128.87, 124.92, 122.62, 119.52, 119.31, 117.77, 116.51, 116.28, 108.60, 66.88, 55.78, 52.90, 41.41.

#### 2.5.5. 1-(1-(3-Fluorobenzoyl)-1*H*-indol-5-yl)-3-(2-(piperidin-1-yl)ethyl)thiourea (**5e**)

White solid, yield: 56%, mp: 187.1–188.1 °C, ^1^H NMR (400 MHz, CDCl_3_): δ 8.41 (d, J = 8.5 Hz, 1H), 8.03 (s, 1H), 7.55–7.50 (m, 2H), 7.43 (d, J = 8.6 Hz, 2H), 7.33 (s, 2H), 7.23 (d, J = 7.9 Hz, 1H), 6.62 (d, J = 3.6 Hz, 1H), 3.65 (s, 2H), 2.45 (s, 2H), 2.28 (s, 4H), 1.33–1.26 (m, 6H). ^13^C NMR (100 MHz, CDCl_3_) δ 166.97, 163.71, 161.23, 136.06, 134.31, 132.70, 131.77, 130.59, 128.56, 124.87, 124.83, 122.47, 119.17, 117.57, 116.45, 108.78, 55.87, 53.83, 41.79, 25.83, 24.10.

#### 2.5.6. 1-(1-(3-Fluorobenzoyl)-1*H*-indol-5-yl)-3-(furan-2-ylmethyl)thiourea (**5f**)

Brown solid, yield: 21%, mp: 152.6–153.6 °C, ^1^H NMR (400 MHz, CDCl_3_): δ 8.42 (s, 1H), 8.13 (s, 1H), 7.51 (s, 2H), 7.46 (s, 2H), 7.33 (s, 3H), 7.28–7.20 (m, 1H), 6.63 (s, 1H), 6.29 (s, 3H), 4.86 (s, 2H). ^13^C NMR (100 MHz, CDCl_3_) δ 181.05, 167.06, 163.71, 161.23, 150.32, 142.41, 135.90, 134.76, 132.04, 130.65, 128.85, 124.89, 119.53, 118.24, 117.93, 116.50, 116.27, 110.50, 108.79, 108.17, 42.41.

#### 2.5.7. 1-(1-(3-Fluorobenzoyl)-1*H*-indol-5-yl)-3-(4-nitrophenyl)thiourea (**5g**)

Yellow solid, yield: 64%, mp: 220.7–221.7 °C, ^1^H NMR (400 MHz, DMSO-d_6_): δ 10.37 (s, 2H), 8.28–8.21 (m, 3H), 7.87 (t, J = 7.3 Hz, 3H), 7.77–7.53 (m, 4H), 7.46–7.40 (m, 2H), 6.80 (d, J = 3.2 Hz, 1H). ^13^C NMR (100 MHz, DMSO-d_6_) δ 180.15, 167.19, 163.44, 160.99, 156.17, 146.86, 142.75, 136.11, 133.11, 131.49, 129.19, 126.87, 125.56, 124.83, 122.51, 122.11, 117.27, 116.13, 112.85, 109.48.

#### 2.5.8. 1-(3,5-Dimethoxyphenyl)-3-(1-(3-fluorobenzoyl)-1*H*-indol-5-yl)thiourea (**5h**)

White solid, yield: 61%, mp: 171.1–172.1 °C, ^1^H NMR (400 MHz, DMSO-d_6_): δ 9.87 (s, 1H), 9.76 (s, 1H), 8.25 (s, 1H), 7.81 (s, 1H), 7.66–7.56 (m, 4H), 7.42 (s, 2H), 6.78–6.74 (m, 3H), 6.30 (s, 1H), 3.74 (s, 6H). ^13^C NMR (100 MHz, DMSO-d_6_) δ 180.12, 167.18, 163.44, 160.68, 141.44, 136.62, 136.02, 133.14, 131.56, 131.22, 129.19, 125.56, 122.50, 119.52, 117.26, 116.49, 116.12, 109.46, 101.96, 96.80, 55.66.

#### 2.5.9. 1-(3,4-Dichlorophenyl)-3-(1-(3-fluorobenzoyl)-1*H*-indol-5-yl)thiourea (**5i**)

White solid, yield: 69%, mp: 193.4–194.4 °C, ^1^H NMR (400 MHz, DMSO-d_6_): δ 10.12 (s, 1H), 9.93 (s, 1H), 8.24 (d, J = 8.8, 1H), 7.93 (d, J = 2.2, 1H), 7.80 (d, J = 1.2, 1H), 7.70–7.56 (m, 5H), 7.48 (dd, J = 8.8, 2.9 Hz, 1H), 7.43 (d, J = 3.7 Hz, 1H), 7.39 (dd, J = 8.8, 1.6 Hz, 1H), 6.79 (d, J = 3.7 Hz, 1H). ^13^C NMR (100 MHz, DMSO-d_6_) δ 180.47, 167.18, 163.44, 161.00, 140.37, 136.50, 135.60, 133.32, 131.56, 131.48, 131.34, 130.87, 130.58, 129.29, 126.38, 125.54, 125.39, 124.14, 122.44, 117.34, 116.50, 116.28, 109.44.

#### 2.5.10. 1-(1-(3-Fluorobenzoyl)-1*H*-indol-5-yl)-3-(pyridin-3-yl)thiourea (**5j**)

White solid, yield: 54%, mp: 144.9–145.9 °C, ^1^H NMR (400 MHz, DMSO-d_6_): δ 8.32 (d, J = 7.2 Hz, 1H), 7.99–7.43 (m, 11H), 6.80 (s, 1H). ^13^C NMR (100 MHz, DMSO-d_6_) δ 180.82, 167.18, 163.44, 161.00, 136.12, 134.48, 133.10, 131.88, 130.77, 129.20, 126.19, 125.56, 123.12, 122.48, 119.55, 118.95, 117.46, 116.66, 116.13, 109.48, 108.84.

### 2.6. General Procedure of Amide Derivatives (***6a***–***e***)

To a dry 25 mL round bottom flask containing a solution of the pre-final amine **3** (0.5 mmol) and DIPEA (0.6 mmol) dissolved in 10 mL of DCM, the appropriate acyl chloride (0.5 mmol) was added. The reaction mixture was stirred overnight at room temperature to yield the desired amide derivative. The target compound was then purified using the same procedure for derivatives **4a**–**n**.

#### 2.6.1. *N*-(1-(3-Fluorobenzoyl)-1*H*-indol-5-yl)-2-nitroisonicotinamide (**6a**)

White solid, yield: 52%, mp: 278.1–279.1 °C, HPLC purity: 17.35 min, 99.12%, ^1^H NMR (400 MHz, DMSO-d_6_): δ 10.94 (s, 1H), 8.90 (d, J = 4.7 Hz, 1H), 8.84 (s, 1H), 8.41 (d, J = 4.4 Hz, 1H), 8.31 (d, J = 8.8 Hz, 1H), 8.24 (s, 1H), 7.71 (d, J = 8.9 Hz, 1H), 7.67–7.61 (m, 3H), 7.56 (t, J = 6.8 Hz, 1H), 7.43 (d, J = 3.5 Hz, 1H), 6.83 (d, J = 3.4 Hz, 1H). ^13^C NMR (100 MHz, DMSO-d_6_) δ 167.18, 163.43, 162.22, 160.99, 157.46, 150.27, 146.74, 136.53, 134.99, 132.80, 131.54, 131.39, 129.44, 128.37, 125.56, 119.27, 118.78, 116.74, 116.26, 113.43, 109.54.

#### 2.6.2. Ethyl (1-(3-fluorobenzoyl)-1*H*-indol-5-yl)carbamate (**6b**)

Yellow solid, yield: 54%, mp: 136.5–137.5 °C, ^1^H NMR (400 MHz, CDCl_3_): δ 8.30 (d, J = 8.8 Hz, 1H), 7.84 (s, 1H), 7.50 (s, 2H), 7.43 (d, J = 8.0 Hz, 1H), 7.32–7.29 (m, 1H), 7.23 (d, J = 3.6 Hz, 1H), 7.18 (dd, J = 8.8, 1.7 Hz, 1H), 4.22 (q, J = 7.2 Hz, 2H), 1.33 (t, J = 7.2 Hz, 3H). ^13^C NMR (100 MHz, CDCl_3_) δ 166.91, 163.69, 161.21, 153.87, 136.49, 134.50, 132.32, 131.52, 130.38, 127.97, 124.77, 119.06, 116.77, 116.15, 110.83, 109.27, 61.26, 14.61.

#### 2.6.3. Ethyl 2-((1-(3-fluorobenzoyl)-1*H*-indol-5-yl)amino)-2-oxoacetate (**6c**)

Off-white solid, yield: 61%, mp: 122.7–123.7 °C, ^1^H NMR (400 MHz, CDCl_3_): δ 9.02 (s, 1H), 8.37 (d, J = 7.6 Hz, 1H), 8.17 (s, 1H), 7.51 (s, 2H), 7.43 (s, 2H), 7.29 (s, 2H), 6.64 (s, 1H), 4.43 (d, J = 5.6 Hz, 2H), 1.44 (s, 3H). ^13^C NMR (100 MHz, CDCl_3_) δ 167.00, 163.69, 161.12, 153.92, 136.18, 133.39, 132.81, 131.41, 130.53, 128.35, 124.86, 119.04, 117.51, 116.95, 116.44, 112.21, 109.26, 63.77, 14.04.

#### 2.6.4. 3,4-Dichloro-*N*-(1-(3-fluorobenzoyl)-1H-indol-5-yl)benzamide (**6d**)

Off-white solid, yield: 54%, mp: 237.6–238.6 °C, ^1^H NMR (400 MHz, DMSO-d_6_): δ 10.52 (s, 1H), 8.30–8.26 (m, 2H), 8.20 (s, 1H), 7.98 (dd, J = 8.4, 1.4 Hz, 2H), 7.83 (d, J = 8.4 Hz, 1H), 7.71–7.60 (m, 4H), 7.55 (t, J = 6.9 Hz, 1H), 7.41 (d, J = 3.6 Hz, 1H), 6.80 (d, J = 3.6 Hz, 1H). ^13^C NMR (100 MHz, DMSO-d_6_) δ 167.13, 163.54, 160.99, 136.59, 135.54, 134.79, 132.51, 131.78, 131.51, 131.35, 131.22, 130.07, 129.25, 128.50, 125.53, 119.44, 118.71, 116.47, 116.35, 116.24, 113.17, 109.56.

#### 2.6.5. *N*-(1-(3-fluorobenzoyl)-1*H*-indol-5-yl)-4-oxo-4*H*-chromene-3-carboxamide (**6e**)

Yellow solid, yield: 66%, mp: 226.6–227.6 °C, ^1^H NMR (400 MHz, DMSO-d_6_): δ 11.46 (s, 1H), 9.22 (s, 1H), 8.32–8.23 (m, 3H), 7.97–7.95 (m, 1H), 7.84 (d, J = 8.0 Hz, 1H), 7.69–7.60 (m, 5H), 7.58–7.53 (m, 1H), 7.43 (d, J = 3.8 Hz, 1H), 6.81 (d, J = 3.6 Hz, 1H). ^13^C NMR (100 MHz, DMSO-d_6_) δ 177.26, 167.13, 163.84, 163.43, 160.99, 60.81, 156.21, 136.64, 135.96, 134.86, 132.52, 131.64, 131.45, 129.49, 127.25, 126.07, 125.51, 123.92, 119.29, 118.02, 116.78, 116.48, 116.18, 112.44, 109.52.

### 2.7. Synthesis of Methyl 1H-indole-5-carboxylate (***10***)

The indole-5-carboxylic acid (**9**) was refluxed in methanol in presence of few drops of sulfuric acid to obtain the corresponding methyl ester **10** as a yellow solid [20]. yield: 72%, ^1^H NMR (400 MHz, DMSO-d_6_): δ 11.49 (s, 1H), 8.28 (s, 1H), 7.72 (dd, J = 8.4, 1.6 Hz, 1H), 7.47 (dd, J = 4.8, 2.0 Hz, 2H), 6.60 (s, 1H), 3.85 (s, 3H).

### 2.8. General Procedure of Methyl Ester Derivatives (***7a*** and ***7b***)

The same procedure for preparing intermediate **2** was used.

#### 2.8.1. Methyl 1-(3-fluorobenzoyl)-1*H*-indole-5-carboxylate (**7a**)

White spheres, yield: 73%, mp: 103.4–104.4 °C, ^1^H NMR (400 MHz, DMSO-d_6_): δ 8.38 (d, J = 8.4 Hz, 1H), 8.35 (d, J = 1.2 Hz, 1H), 8.00 (dd, J = 8.8, 1.6 Hz, 1H), 7.68–7.62 (m, 3H), 7.57–7.54 (m, 2H), 6.91 (d, J = 3.6 Hz, 1H), 3.90 (s, 3H). ^13^C NMR (100 MHz, DMSO-d_6_) δ 167.52, 166.84, 163.41, 160.97, 138.46, 136.25, 131.50, 131.13, 130.24, 126.05, 125.72, 123.36, 119.77, 116.66, 116.29, 109.53, 52.59.

#### 2.8.2. Methyl 1-(3,4-dichlorobenzoyl)-1*H*-indole-5-carboxylate (**7b**)

White solid, yield: 70%, mp: 157.4–158.4 °C, ^1^H NMR (400 MHz, DMSO-d_6_): δ 8.38 (d, J = 8.56 Hz, 1H), 8.35 (s, 1H), 8.08 (s, 1H), 8.00 (d, J = 8.4 Hz, 1H), 7.88 (d, J = 8.1 Hz, 1H), 7.77 (d, J = 7.8 Hz, 1H), 7.59 (d, J = 3.0 Hz, 1H), 6.91 (d, J = 2.8 Hz, 1H) 3.91 (s, 3H). ^13^C NMR (100 MHz, DMSO-d_6_) δ 166.83, 165.91, 138.46, 135.52, 134.57, 132.17, 131.54, 131.52, 131.16, 130.31, 129.69, 126.08, 125.78, 123.34, 116.31, 109.65, 52.60.

### 2.9. General Procedure of the Free NH Indole Derivatives ***8a***–***e***

To a dry 25 mL round bottom flask containing a solution the indole-5-carboxylic acid (**9**, 1.15 mmol) in DMF (5 ml), DIPEA (2.5 mmol) was added, and the mixture stirred for 10 min. HATU (1.15 mmol) was then added, and the mixture was further stirred for 20 min. Finally, the appropriate amine (1 mmol) was added, and the reaction mixture was heated at 80 °C overnight. The reaction mixture was then purified by flash column chromatography (SiO_2_, DCM:EA, 9:1) to yield the final compounds.

#### 2.9.1. *N*-(4-iodophenyl)-1*H*-indole-5-carboxamide (**8a**)

Off-white solid, yield: 73%, mp: 214.8–215.8 °C, HPLC purity: 16.63 min, 97.34%, ^1^H NMR (400 MHz, DMSO-d_6_): δ 11.42 (s, 1H), 10.23 (s, 1H), 8.28 (s, 1H), 7.74 (d, J = 8.6 Hz, 1H), 7.69 (s, 4H), 7.52–7.48 (m, 2H), 6.61 (s, 1H). ^13^C NMR (100 MHz, DMSO-d_6_) δ 167.13, 140.11, 138.17, 137.64, 127.47, 127.44, 125.98, 122.83, 121.43, 121.01, 111.59, 102.75, 87.03.

#### 2.9.2. *N*-(4-bromophenyl)-1*H*-indole-5-carboxamide (**8b**)

Off-white solid, yield: 71%, mp: 208.6–209.6 °C, HPLC purity: 15.73 min, 95.64%, ^1^H NMR (400 MHz, DMSO-d_6_): δ 11.42 (s, 1H), 10.25 (s, 1H), 8.28 (s, 1H), 7.81 (d, J = 8.8 Hz, 2H), 7.73 (dd, J = 8.6, 1.5 Hz, 1H), 7.55 (s, 1H), 7.53–7.48 (m, 3H), 6.61 (s, 1H). ^13^C NMR (100 MHz, DMSO-d_6_) δ 167.14, 139.62, 138.17, 131.80, 127.47, 127.44, 125.94, 122.53, 121.43, 121.01, 115.19, 111.60, 102.75. HRMS (ESI) *m*/*z* calculated for C_15_H_11_BrN_2_O [M + H]^+^: 315.0133 Found: 315.0123.

#### 2.9.3. *N*-(3-chloro-4-fluorophenyl)-1*H*-indole-5-carboxamide (**8c**)

White solid, yield: 67%, mp: 213.4–214.4 °C, HPLC purity: 12.87 min, 97.28%, ^1^H NMR (400 MHz, DMSO-d_6_): δ 11.87 (s, 1H), 8.86–8.68 (m, 3H), 7.98 (s, 1H), 7.69–7.65 (m, 3H), 6.77 (s, 1H). ^13^C NMR (100 MHz, DMSO-d_6_) δ 164.01, 153.07, 140.70, 140.42, 135.00, 130.33, 128.93, 128.25, 125.53, 123.21, 122.19, 113.91, 113.07, 103.86.

#### 2.9.4. *N*-(benzo[*d*][1,3]dioxol-5-ylmethyl)-1*H*-indole-5-carboxamide (**8d**)

White solid, yield: 51%, mp: 131.6–132.6 °C, ^1^H NMR (400 MHz, DMSO-d_6_): δ 8.63 (s, 1H), 8.11 (s, 1H), 7.64 (d, J = 8.6, 1.5 Hz, 1H), 7.37 (d, J = 8.6 Hz, 1H), 7.24 (s, 1H), 6.87 (s, 1H), 6.82 (d, J = 7.9 Hz, 1H), 6.76 (d, J = 7.9 Hz, 1H), 6.59 (s, 1H), 6.44 (s, 1H), 5.94 (s, 2H), 4.57 (d, J = 5.6 Hz, 2H). ^13^C NMR (100 MHz, DMSO-d_6_) δ 168.76, 147.92, 146.93, 137.73, 132.43, 127.55, 125.94, 125.88, 121.08, 120.90, 120.14, 111.22, 108.44, 108.32, 103.34, 101.04, 43.98. HRMS (ESI) *m*/*z* calculated for C_17_H_14_N_2_O_3_ [M + H]^+^: 295.1083. Found: 295.1072.

#### 2.9.5. *N*-(benzo[*d*][1,3]dioxol-5-yl)-1*H*-indole-5-carboxamide (**8e**)

White solid, yield: 46%, mp: 198.9–199.9 °C, ^1^H NMR (400 MHz, DMSO-d_6_): δ 11.39 (s, 1H), 10.03 (s, 1H), 8.26 (s, 1H), 7.72 (d, J = 8.3 Hz, 1H), 7.51–7.48 (m, 3H), 7.24 (d, J = 7.8 Hz, 1H), 6.89 (d, 1H), 6.60 (s, 1H), 6.02 (s, 2H). ^13^C NMR (100 MHz, DMSO-d_6_) δ 166.77, 147.38, 143.32, 138.04, 134.58, 127.47, 127.33, 126.25, 121.35, 120.78, 113.55, 111.52, 108.33, 102.89, 102.69, 101.35.

### 2.10. MAO Assays

The biological evaluation of the tested compounds over both MAO-A and MAO-B was performed following the previously reported method [21]. The MAO activity was measured in relative fluorescence units (RFU) evoked by peroxidase-catalyzed oxidation of Amplex Red^®^ to resorufin, in which H_2_O_2_ generated by MAO reaction was used as the electron donor. Human recombinant MAO-A (*h*MAO-A) and MAO-B (*h*MAO-B) enzymes expressed in insect cells were obtained from Sigma-Aldrich. The 2 μL of test compound in DMSO (final concentration: 1–10 μM) was treated with 98 μL of *h*MAO enzyme solution in 50 mM sodium phosphate buffer (pH 7.4, final protein amounts: ~1.25 μg protein/well for MAO-A and ~2.5 μg protein/well for MAO-B) on 96-well black plate and incubated for 15 min at 37 °C. Then, 100 μL of reaction working solution which is a mixed solution of 400 μM Amplex Red^®^ (Cayman, final concentration: 200 μM), 2 U/mL horseradish peroxidase (Sigma-Aldrich, final concentration: 1 U/mL) and 2 mM substrate (p-tyramine for MAO-A, benzylamine for MAO-B, Sigma-Aldrich, final concentration: 1 mM) in 50 mM sodium phosphate buffer (pH 7.4) were added and incubated for 20 min at 37 °C in the dark. The fluorescent intensity was quantified using a microplate reader (SpectraMax^®^i3, Molecular Device) with an excitation at 545 nm and an emission at 590 nm. The 50% inhibitory concentrations (IC_50_) of compounds were determined as the mean ± S.E.M. in triplicate from the dose-response inhibition curves using SigmaPlot^®^ 13.0 (Systat Software Inc., San Jose, CA, USA).

### 2.11. Kinetic Study of MAO-B Inhibition Mode

The type of MAO-B inhibition of the tested compounds was determined by Michaelis-Menten kinetic study following our recently reported study [18]. The type of MAO-B inhibition of the tested compound(s) was determined by Michaelis–Menten kinetic study. The catalytic rates of the *h*MAO-B enzyme in the absence or in the presence of three different concentrations (10, 30, and 100 nM) of the tested compound were measured at seven different concentrations of the benzylamine (0.065, 0.125, 0.25, 0.5, 1, 2, and 4 mM). The corresponding progression curves and the Lineweaver-Burk plots were generated using GraphPad Prism 7 (GraphPad Software, Inc., La Jolla, CA, USA). The maximal velocity (V_max_), the Michaelis constant (K_m_), and the inhibition constant (K_i_) were calculated using SigmaPlot^®^ 13.0.

### 2.12. Molecular Modeling Study

The protein X-ray crystal structures of human MAO-B bound to safinamide (PDB ID: 2V5Z) [22] and MAO-A bound to harmine as a selective MAO-A inhibitor with an IC_50_ of 0.006 μM [23] (PDB ID: 2Z5X) [24] were first obtained from the RCSB protein data bank (www.rcsb.org, accessed on 28 August 2021) and imported into the Schrodinger Maestro software 2017 suite (Maestro, Schrödinger, LLC, New York, NY). The protein preparation was done under default settings and at pH = 7.4 in the Maestro protein preparation wizard. In the MAO-B crystal structure, crucial water molecules 1192, 1193, 1247, 1360, and 1364 were preserved. All compounds were sketched in ChemDraw Professional 16.0 and imported as 2D structure data files into the Maestro LigPrep module. The ligand preparation protocol generated the energy-minimized 3D ligand conformations, as well as determined the ligand ionization states, tautomeric states, and generated all possible isomers. Maestro Glide’s extra precision docking mode was used to dock the compounds into the ligand-centered grid and outputted up to five poses for each ligand. The setup docking protocols were considered appropriate and accurate since they reproduced the binding modes of the native bound inhibitors with high accuracy: RMSD equal to 0.74 and 0.27 Å, respectively, for safinamide and harmine.

### 2.13. In Vitro Cellular and Cell-Free Bio-Assay

#### 2.13.1. Materials

Rat adrenal pheochromocytoma cell line (PC12) was purchased from American Type Culture Collection (CRL-1721). RPMI-1640 medium, fetal bovine serum (FBS), horse serum (HS), and antibiotic–antimycotic agents were obtained from Gibco BRL (Grand Island, NY, USA). 6-Hydroxydopamine (6-OHDA) was supplied from Tocris Bioscience (Bristol, UK), and dimethyl sulfoxide (DMSO), 2,2-diphenyl-1-picrylhydrazyl (DPPH), laminin, 3-(4,5-dimethyldiazol-2-yl)-2,5-diphenyltetrazolium bromide (MTT). and rasagiline mesylate were from Sigma-Aldrich (St. Louis, MO, USA).

#### 2.13.2. PC12 Cell Culture

PC12 cells were routinely maintained in RPMI-1640 media containing 100 IU/mL penicillin, and 100 μg/mL streptomycin supplemented by 10% heat-inactivated HS, 5% FBS in a humidified incubator containing 5% CO_2_ and 95% air at 37 °C [25]. The cell culture media was changed every two days. To perform experiments, cells were plated on poly-D-lysine/laminin-coated 24-well plates at a density of 3 × 10^5^ cells/well and incubated for at least 24 h to allow cell adhesion.

#### 2.13.3. Drug Treatment

To examine the effects of the test compounds on cell survival, cells were incubated in low serum media (1% HS, 1% FBS, 100 IU/mL penicillin, and 100 μg/mL streptomycin) with test compounds at the concentrations of 10 and 30 μM or an equivalent volume of vehicle control (DMSO at the final concentration of 0.3%) for 24 h. The media containing test compounds was sonicated at room temperature for 30 min to assure adequate dissolution in the media. To assess the protective effects of the test compounds on 6-OHDA- or rotenone-induced cell death in PC12 cells, cells were pre-treated with the compounds or rasagiline for 4 h. The pre-treated cells were then exposed to 6-OHDA (50 μM) or rotenone (1 μM) for 24 h [26,27].

#### 2.13.4. Measurements of Cell Viability

After the desired treatment, cell viability was determined by MTT assay as described previously [28], with slight modification. Briefly, MTT was added to the cells at the final concentration of 0.5 mg/mL, followed by incubation for 3 h at 37 °C. The supernatants were carefully removed, and then 300 μL of DMSO was added to each well to dissolve formazan precipitate. The absorbance of the dissolved solution was measured at 550 nm using a microplate reader (SpectraMax M2e, Molecular Devices, Sunnyvale, CA, USA). The cell viability was presented as the percentage of the absorbance measured in the vehicle-treated control cells. Percent inhibition was calculated using the following formula and analyzed by non-linear regression using GraphPad Prism 5 (GraphPad Software, Inc., La Jolla, CA, USA) to determine the concentrations exhibiting 50% inhibition (IC_50_).

Inhibition (%) = 100 × (Abs_sample_ − Abs_6-OHDA or rotenone_)/(Abs_control_ − Abs_6-OHDA or rotenone_)

#### 2.13.5. Measurement of ROS Production Using H_2_-DCFDA

The involvement of oxidative stress in the observed reduction in cell viability was investigated using the fluorescent dye H_2_-DCFDA as described previously [29]. Briefly, cells were loaded with 10 μM H_2_-DCFDA for 30 min at 37 °C. Subsequently, cells were exposed for 4 h to 10 μM of test compounds followed by the addition of 50 μM 6-OHDA or 1 μM rotenone for 20 h, and fluorescence was measured spectrophotometrically at 488/520 nm in a microplate reader (SpectraMax M2e). N-acetylcysteine (NAC, 5 mM) was included as a negative control for oxidative stress.

#### 2.13.6. Assessment of DPPH Radical Scavenging Activity

DPPH radical scavenging activities of test compounds were assessed as previously described [30]. Briefly, the reaction mixture containing desired concentrations of test compounds and DPPH radical solution (150 µM) prepared in 95% methanol was incubated for 30 min at 37 °C. The absorbance of the mixture was measured at 520 nm in a microplate reader (SpectraMax M2e). The radical scavenging activity was calculated using the following equation:

DPPH radical scavenging activity (%) = 100 × (Abs_control_ − Abs_sample_)/Abs_control_

where Abs_control_ and Abs_sample_ represent the absorbances in the absence and in the presence of test compounds, respectively.

#### 2.13.7. Statistical Analysis

All data are presented as the mean ± SEM of at least three independent experiments. Statistical significances were analyzed by one-way analysis of variance (ANOVA) followed by Tukey’s post hoc test with SigmaPlot 12.5 software. A *p* < 0.05 was considered statistically significant.

## 3. Results and Discussion

### 3.1. Chemical Synthesis

**Scheme 1:**
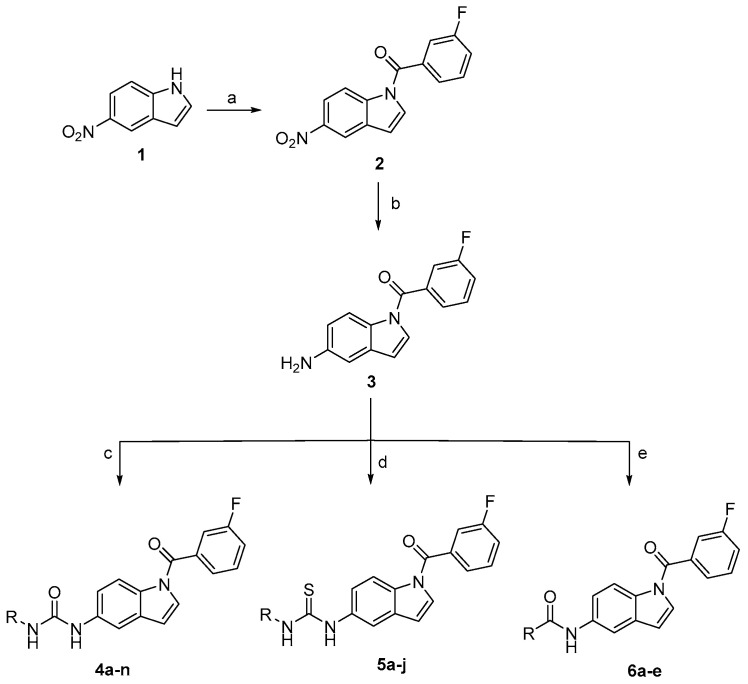
Reagents and conditions: (**a**) 3-fluorobenzoyl chloride, NaOH, TBAHS, DCM, rt, 2 h; (**b**) H_2_, 10% Pt/C, EtOAc, rt, 75 min; (**c**) appropriate isocyanate reagent, DIPEA, DCM, rt, overnight; (**d**) appropriate isothiocyanate reagent, acetonitrile, 80 °C, 6 h; (**e**) appropriate acid chloride, DIPEA, DCM, rt, overnight.

**Scheme 2:**
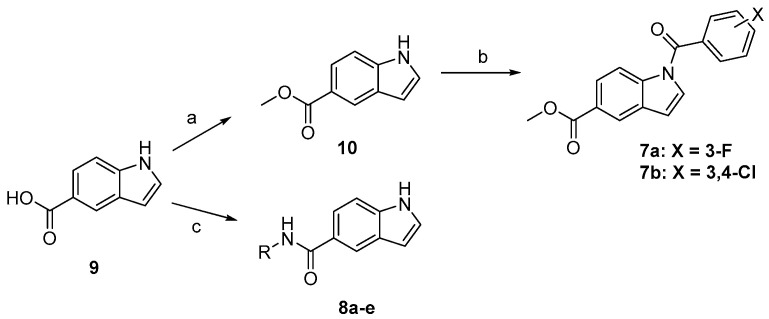
Reagents and conditions: (**a**) H_2_SO_4_, methanol, reflux, overnight; (**b**) 3-fluorobenzoyl chloride, NaOH, TBAHS, DCM, rt, 2 h; (**c**) appropriate amine, HATU, DIPEA, DMF, 80 °C, overnight.

The newly synthesized target compounds **4a**–**n**, **5a**–**j**, and **6a**–**e** were prepared as outlined in Scheme 1 and fully described in Table 1. Starting from the commercially available 5-nitroindole (**1**), (3-fluorophenyl)(5-nitro-1*H*-indol-1-yl)methanone (**2**) was prepared by substitution on the N1 position of the indole core using 3-fluorobenzoyl chloride in dichloromethane solvent (DCM) and in the presence of NaOH as a base, in addition to tetrabutylammonium hydrogensulfate (TBAHS) as a phase transfer reagent at room temperature. Preparation of the intermediate **3** was performed by reducing the 5-nitro group of compound **2** under an atmosphere of hydrogen using platinum as a metal catalyst and ethyl acetate as the solvent. The target final compounds were obtained either by stirring the pre-final amine (**3**) with the appropriate isocyanate in the presence of *N,N*-Diisopropylethylamine (DIPEA) for **4a**–**n**, or by refluxing the pre-final (**3**) with the corresponding isothiocyanate in acetonitrile for 6 h to get the final compounds **5a**–**j**. The amide compounds **6a**–**e** were obtained by stirring compound **3** with the suitable acid chloride in DCM in the presence of DIPEA. The final ester derivatives **7a** and **7b** were prepared as depicted in Scheme 2 by forming the methyl ester of the starting material 5-carboxyindole (**9**), followed by introducing the appropriate halobenzoyl moiety on the indole N1 position. For the amide derivatives **8a**–**e**, compound **9** was coupled with the appropriate amine using HATU as a coupling agent, DIPEA as a base, and DMF as the solvent. The final compounds were confirmed through ^1^HNMR spectroscopy by the appearance of peaks specific to the alkyl or aryl moieties coupled with the amine group at the C5 position of the indole. This was further supported by the appearance of an extra ^13^CNMR peak between 160–180 ppm, characteristic of the C=O or C=S carbons of the newly formed linker.

### 3.2. Primary Screening at Single Dose of 10 µM over MAO-B

As shown in Table 1, all target small molecules **4a**–**n**, **5a**–**j**, **6a**–**e**, **7a**, 7**b**, and **8a**–**e** were biologically assessed over human MAO-B recombinant enzyme at a single dose of 10 µM. When the fourteen urea analogs (**4a**–**n**) were evaluated, compound **4a** with an ethyl group showed modest inhibition of 46.2%. For derivatives **4b** and **4c**, bearing the aliphatic moieties ethyl acetate and 2-chloroethyl, they showed lower inhibitory activity of 9.9% and 26.6%, respectively. It was found that the introduction of a cyclohexyl ring in **4d** slightly restored the inhibition to the level of 44%. For the urea compounds possessing aromatic moieties, compound **4e** having p-nitro group showed inhibition of 47.9%, which was lowered in **4f** with a methoxy replacement to reach 32.5%. Interestingly, the inhibition was nearly doubled to 55.7% in **4g** with 3,5-dimethoxy substitution. For derivatives **4h**–**l** with halogen substituents, compound **4j** with the preferred 3,4-dichloro substitution pattern showed the highest activity with 53.5%. For compounds **4m** and **4n** possessing 4-phenoxyphenyl and 4-morpholinophenyl motifs, activities were lowered to 38.1% and 32.9%, respectively. Regarding the thiourea derivatives, while compound **5a** with an ethyl formate substitution showed diminished activity of 15.5%, compounds **5b** and **5c** with 2-chloroethyl and 4-morpholinophenyl moieties, respectively, showed higher inhibitory activities of 45.6 and 43.9%, respectively. Replacing the phenyl spacer in **5c** with an ethyl linker in **5d** showed only a minor increase in activity to reach 46.8%. Changing the morpholine ring in **5d** to a piperidine ring in **5e** while keeping the same ethyl linker showed almost the same activity 46.8 and 44.7%, respectively. Compounds **5f**–**h** with furanyl-2-methyl, 4-nitrophenyl, and 3,5-dimethoxyphenyl substitutions exhibited modest activities of 34.1%, 30.6%, and 32.5%, respectively. Compound **5i** confirmed the superiority of the 3,4-dichlorophenyl moiety, showing an improved inhibition performance of 59.8%. Compound **5j** with a 3-pyridinyl moiety exhibited moderate inhibition of 51.1%.

For the amide derivatives **6a**–**e**, compound **6a** with a 2-nitroisonicotinamide showed low inhibition of 26.8%. Analogs **6b** and **6c** with ethyl formate and ethyl 2-oxoacetate showed a similar activity of 33.8% and 35.5%, respectively. Compound **6d** with 3,4-dichlorophenyl moiety further supported the preference of the moiety with a 53% inhibition activity. In compound **6e**, a chromone (1,4-benzopyrone) preferred moiety with proved activity towards MAO-B inhibition was introduced, giving a satisfactory inhibition rate of 59.6%. Finally, we tried to explore the effect of changing the linker to an ester linker while using a short alkyl substituent. Compound **7a**, with a methyl ester substitution, exhibited a modest inhibition activity of 49.8%. Changing the 3-fluorobenzoyl moiety to 3,4-dichlorobenzoyl in **7b** showed a distinguished inhibition percent of 84.1%. For the free (NH) indole derivatives, they showed much better overall inhibition values, where compound **8e** elicited 89.6% inhibition, while compounds **8a** and **8b** showed complete inhibition (>99%) for the enzyme at the tested concentration.

### 3.3. Dose-Dependent Assay over MAO-B

**Figure 3:**
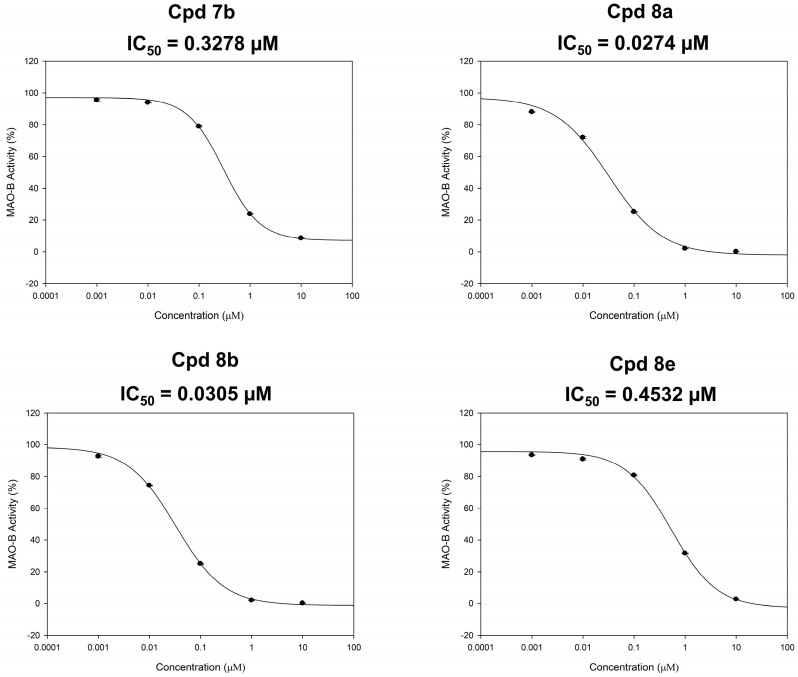
Dose-dependent assays of compounds **7b**, **8a**, **8b**, and **8e** over MAO-B.

The inhibitory potencies (IC_50_ values) of the most active indole-based derivatives **7b**, **8a**, **8b**, and **8e**, producing inhibition of MAO-B activity > 70%, were measured in triplicate from the dose–response inhibition curves using Sigma-Plot software version 13.0 (Figure 3) using five doses assay over MAO-B with 10, 1, 0.1, 0.01, and 0.001 μM concentrations of the tested compounds. While compounds **7b** and **8e** exerted submicromolar IC_50_ values of 0.3278 and 0.4532 µM (Figure 3), a higher potency in the nanomolar range was exhibited by derivatives **8a** and **8b** (0.0274 and 0.0305 µM, respectively).

### 3.4. Selectivity Assay of Compounds ***7b***, ***8a***, ***8b***, and ***8e*** over Both Isoforms of MAO

Non-selective MAO inhibitors are reported to play a major role in various complications when taken with tyramine-containing nutrients since the inhibition of MAO-A may cause hypertensive symptoms due to a risky boost of serum tyramine levels. Thus, selective MAO-B inhibitors can eliminate this risk by preferentially inhibiting MAO-B. Accordingly, to check the selectivity of the most potent MAO-B inhibitors in this series and to calculate their selectivity index (SI), the IC_50_ values of compounds **7b**, **8a**, **8b**, and **8e** over MAO-A have been identified and found to be more than 100 µM (Table 2). As a result, the selectivity indices were further calculated through the ratio of IC_50_ (MAO-A)/IC_50_ (MAO-B) as demonstrated in Table 2. Compared to the modest selectivity index of rasagiline (**II**, calculated SI > 50), it was found that all the tested compounds were more selective for the MAO-B isoform. While compounds **7b** and **8e** showed good selectivity indices (>305 and 220, respectively), compounds **8a** and **8b** exhibited outstanding SI values (>3649 and 3278, respectively). Accordingly, compounds **8a** and **8b** were selected for further evaluation to define their kinetic mode of interaction with the human MAO-B enzyme.

### 3.5. Kinetic Study to Define the Interaction Mode of Compounds ***8a*** and ***8b*** with MAO-B

**Figure 4:**
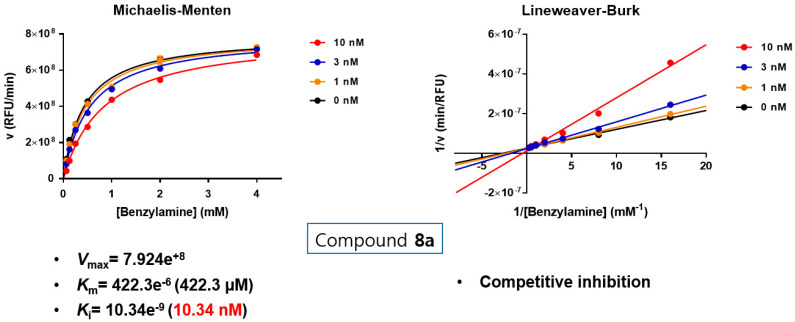
Type of inhibition of MAO-B by compound **8a**. The catalytic rates were measured at different concentrations of benzylamine (0.065, 0.125, 0.25, 0.5, 1, 2, and 4 mM) in the absence and in the presence of different concentrations (10, 30, and 100 nM) of compound **8a**. The V_max_, K_m_ and K_i_ value were calculated using SigmaPlot^®^.

**Figure 5:**
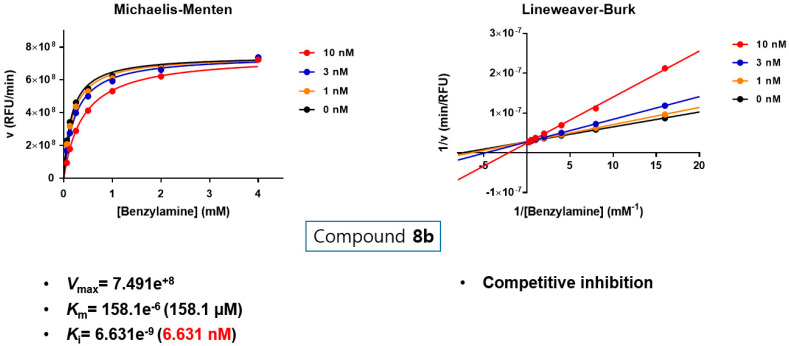
Type of inhibition of MAO-B by compound **8b**. The catalytic rates were measured at different concentrations of benzylamine (0.065, 0.125, 0.25, 0.5, 1, 2, and 4 mM) in the absence and in the presence of different concentrations (10, 30, and 100 nM) of compound **8b**. The V_max_, K_m_, and K_i_ value were calculated using SigmaPlot^®^.

Substrate-dependent kinetic tests were performed to evaluate the mode of MAO-B inhibition of the most potent two compounds **8a** and **8b**. The initial rates of MAO-B inhibition in the absence and presence of the tested compound were measured at various concentrations (0.065, 0.125, 0.25, 0.5, 1, 2, and 4 mM) of benzylamine (a selective substrate for MAO-B). The corresponding progression curves and the Lineweaver-Burk plots were generated using GraphPad Prism 7. Both Michaelis–Menten and the Lineweaver-Burk plots were depicted in Figure 4 and Figure 5. The Lineweaver–Burk plots for three different concentrations of the tested compounds (10, 30, and 100 nM) were found to be linear and intersected at the y-axis. The maximal velocity (V_max_), the Michaelis constant (K_m_), and the inhibition constant (K_i_) were calculated using SigmaPlot^®^. While compound **8a** showed V_max_ = 7.924e^+8^ mol/sec, K_m_ = 422.3e^−6^ (422.3 µM) and K_i_ = 10.34e^−9^ (10.34 nM), compound **8b** exhibited V_max_ = 7.491e^+8^ mol/sec, K_m_ = 158.1e^−6^ (158.1 µM) and K_i_ = 6.631e^−9^ (6.631 nM). Hence, it can be stated that both compounds are competitive, and accordingly, reversible MAO-B inhibitors.

### 3.6. Molecular Modeling Study

To fully understand the structure-activity relationship of the N-substituted-indol-5-yl)benzamide derivatives and their selectivity, the novel compounds were docked into the MAO-B and MAO-A binding pockets. Crystal structures of safinamide bound to MAO-B and harmine bound to MAO-A (PDB ID: 2V5Z [22] and 2Z5X [24], respectively) were used for the molecular docking studies. Validation of the docking protocol was based on its ability to correctly reproduce the binding poses of the co-crystalized active compounds with RMSD 0.74 Å and 0.3 Å for safinamide and harmine respectively (Figure S1A,B). Unlike rasagiline, which is an irreversible covalent inhibitor (Figure S1C), the novel indole-based compounds are reversible non-covalent inhibitors.

The obtained in silico binding modes in MAO-B clarify the following observed SAR trends:

- The active N-substituted indole compound **7b** has similar activity to the free (NH) indole derivatives.
- The 3,4-dichlorophenyl substituent is superior to the alkyl substitutions.
- Among the 3,4-dichlorophenyl compounds, the thiourea linker is the most active, whereas in the alkyl substituent series it is the least active.
- The active N-substituted indole and free (NH) indole compounds are highly selective towards MAO-B.

**Figure 6:**
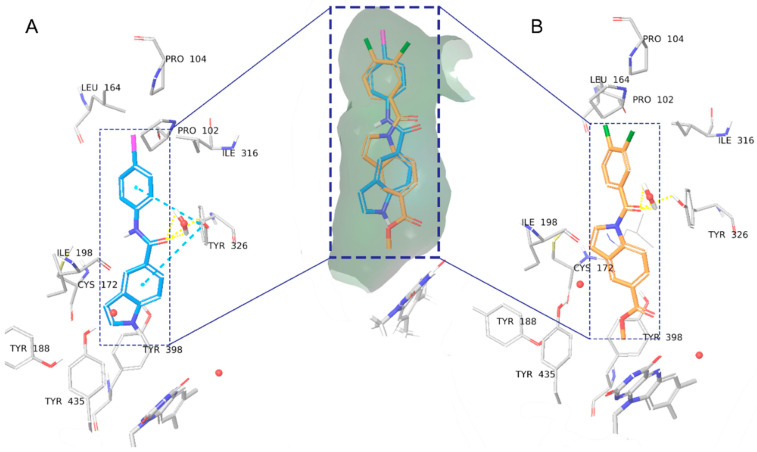
Binding modes of compounds **8a** (**A**) and **7b** (**B**) in the binding pocket of MAO-B. Water molecules are shown as red spheres, with explicit hydrogens shown only when involved in hydrogen bonds. H-bonds are shown with yellow dashed lines, π-π interactions are shown as sky blue dashed lines.

Although the binding modes and crucial interactions of free (NH) indoles have been discussed in detail [19], compounds **8a**, **8b**, and **8e** were found to bind slightly deeper into the binding pocket, closer to FAD, than previously investigated compounds due to the large size of the benzamide halogen substituents. Overall, in compound **8a** (Figure 6A, glide score −10.013), the amide linker makes H-bond interactions with the HOH1247 water “anchor” and crucial residue Tyr326, whereas the indole moiety is involved in π-π interactions with Tyr398. The bromine, iodine, or benzodioxole moieties form hydrophobic π-sigma, π-alkyl, or alkyl-alkyl interactions with Pro104, Trp119, Leu164, Leu167, and Ile316. The N-substituted compound **7b** (Figure 6B, glide score −11.159) shares the same hydrophobic interactions. The H-bonds with HOH1247 and Tyr326 are formed through the carbonyl linker between the indole and the dichlorobenzene moiety, thus having a different position of the indole ring. In the case of **7b**, the indole group is further away from Tyr398 and is centered by π-sulfur interactions with Cys172 and π-π T-shaped interactions with Tyr326. The weaker interactions could explain the higher activity of **8a** over **7b**.

**Figure 7:**
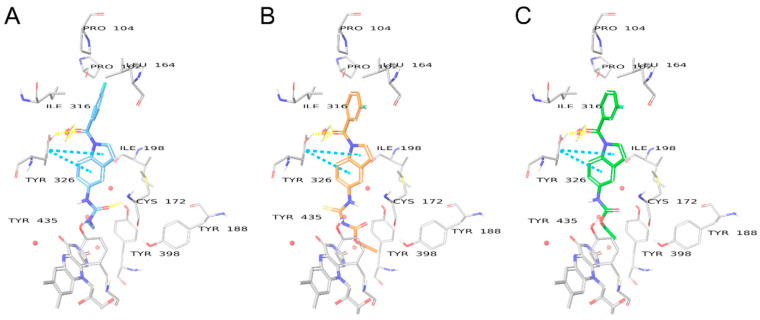
Binding modes of compounds **4a** (**A**), **5a** (**B**), and **6b** (**C**) in the binding pocket of MAO-B. Water molecules are shown as red spheres, with explicit hydrogens shown only when involved in hydrogen bonds. H-bonds are shown with yellow dashed lines, π-π interactions are shown as sky blue dashed lines.

The described binding mode for compound **7b** is common for all N-substituted-indol-5-yl)benzamide derivatives. However, the compounds from the urea, thiourea, and amide linker series all have lower docking scores than **7b**, which implies lower binding affinity. In the case of the alkyl-type C5 substituents, the most active compound **4a** has a docking score of −9.348 and forms a H-bond with Tyr435 through the oxygen atom of the urea linker (Figure 7). The same H-bond is formed by the amide linker of compound **6b** but has a lower docking score equal to −7.437, which is consistent with its lower MAO-B activity. Compounds of the thiourea series fail to form such a H-bond, which could explain why the activity diminishes for compound **5a** (docking score −7.881).

**Figure 8:**
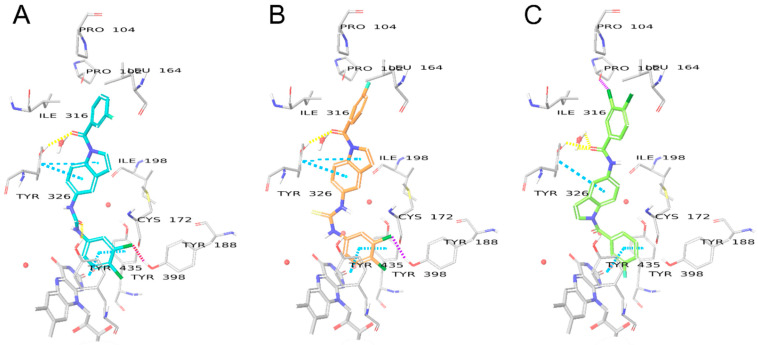
Binding modes of compounds **4j** (**A**), **5i** (**B**), and **6d** (**C**) in the binding pocket of MAO-B. Water molecules are shown as red spheres, with explicit hydrogens shown only when involved in hydrogen bonds. H-bonds are shown with yellow dashed lines, π-π interactions are shown as sky blue dashed lines, halogen interactions are shown as purple dashed lines.

The higher activity of the 3,4-dichlorophenyl derivatives (as compared to the alkyl substituents) occurs because of favorable π-π stacking interactions with Tyr398 and Tyr435, which are sometimes referred to as the “aromatic cage” [31] (Figure 8). The role of the linkers, in this case, is to correctly position the benzene ring for optimal interactions. The thiourea compound **5i** (docking score −11.266) in this case is the most active, compared to **4j** (docking score −10.616) and **6d** (docking score −10.679), since it forms additional π-sulfur interactions with Phe343 and Tyr326. However, it seems that the pyrazinyl heterocyclic ring of compound **VIII** may be more favorable, since it forms not only π-π stacking interactions but also H-bonds with the nearby tyrosine residues [18].

**Figure 9:**
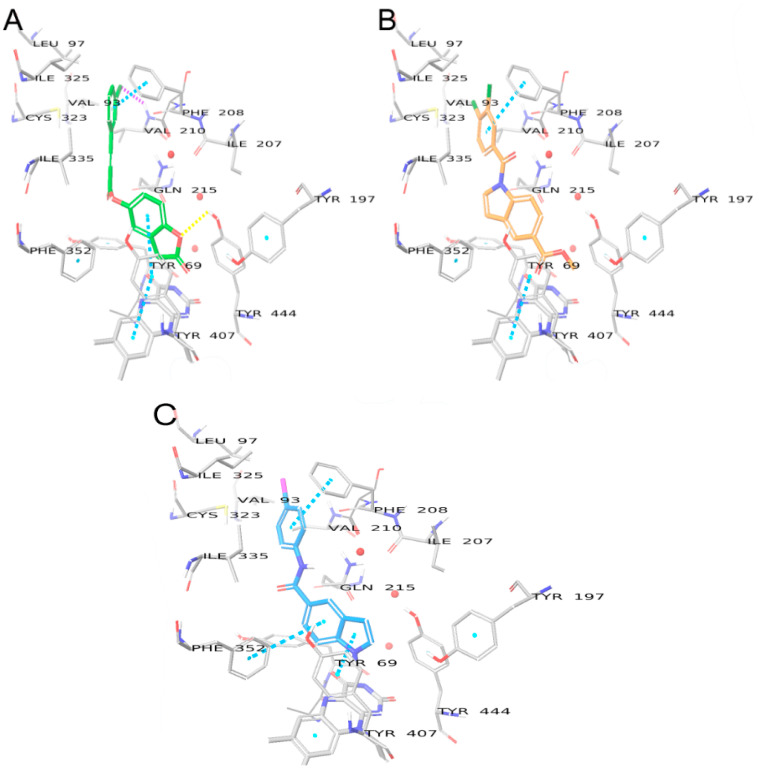
Binding modes of reference benzofuran compound [32] (**A**), **8a** (**B**), and **7b** (**C**) in the binding pocket of MAO-A. Water molecules are shown as red spheres. H-bonds are shown with yellow dashed lines, π-π interactions are shown as sky blue dashed lines, halogen interactions are shown as purple dashed lines.

Free (NH) indole compounds have previously been shown to be highly selective towards MAO-B over MAO-A. It has been rationalized that the main cause of the selectivity is due to the differences in the amino acid sequence of MAO-A and MAO-B. Specifically, the crucial gate residue Tyr326, which is involved in H-bonding with the indole core compounds in MAO-B corresponds to Ile335 in MAO-A. As well as the residue Cys172 in MAO-B, which is also involved in the positioning of the indole ring is replaced by Asn181 in MAO-A [18]. In the absence of these crucial interactions, the binding modes of the compounds are not stable in MAO-A. Prins et. al. demonstrated that benzofuran compounds can show micro-molar activity for MAO-A, as their binding modes are stabilized by interactions with Tyr444, Tyr407 and water molecules close to FAD in MAO-A [32]. The overall shape of the binding mode of compounds **8a** and **7b** to MAO-A resembles the benzofuran reference (Figure 9), however, unlike the MAO-A active reference (docking score −7.470), **8a** and **7b** fail to form favorable H-bonds via their indole core, which does not include as many acceptor atoms as benzofuran. The amide linker in **8a** and carbonyl linker in **7b** also do not form any favorable bonds, as opposed to their binding mode in MAO-B. As a result, the compounds have significantly lower docking scores (−5.694 and −0.265 for **8a** and **7b**, respectively). Therefore, their activity in MAO-A is much lower than in MAO-B.

### 3.7. Biological Evaluation Using PC12 Cells and Cell-Free Bio-Assay

#### 3.7.1. Assessment of the Cytotoxicity over PC12 Cells

**Figure 10:**
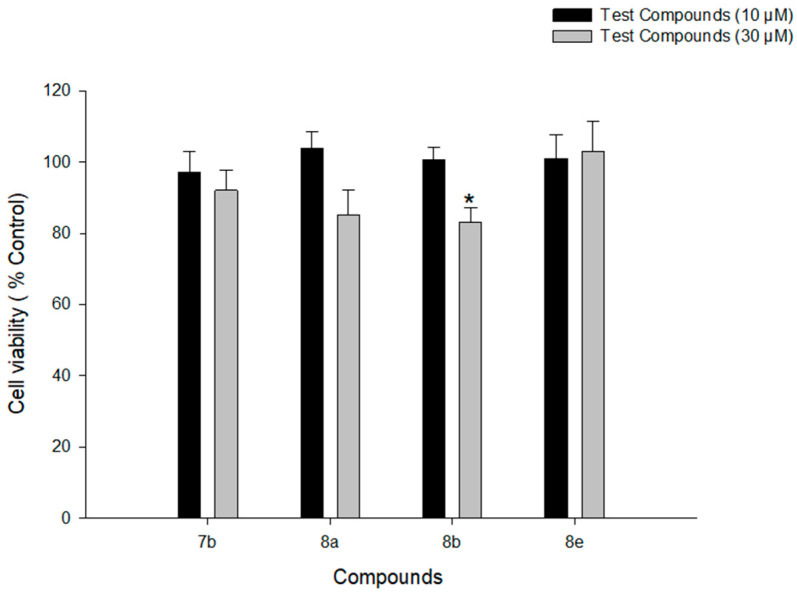
Effects of the test compounds on the viability of PC12 cells. PC12 cells were treated with test compounds at the concentrations of 10 and 30 μM for 24 h and MTT assays were performed as described in the Materials and methods. Data are expressed as the mean ± SEM of at least three independent experiments. * *p* < 0.05 vs. vehicle-treated control cells.

As previously stated, PD primarily damages dopaminergic neurons in the substantia nigra pars compacta. Because primary cells are difficult to obtain and maintain, most of the ongoing PD research is done with well-established cell models such as PC12 cells. Furthermore, because low nonspecific cytotoxicity is an important property of any novel therapeutic candidate, we started the cell-based biological assay with a cytotoxicity test in PC12 cells for the most active compounds (**7b**, **8a**, **8b**, and **8e**) to evaluate their cytotoxic profiles. These cells have been shown to produce dopamine neurotransmitters and have a high concentration of dopamine transporters. The cell line is generated from rat pheochromocytoma and is often used as an in vitro model to research drug neurotoxicity on central dopaminergic neurons and to explore neurotherapeutic studies for PD [33,34]. Accordingly, PC12 cells were incubated with the test compounds at concentrations of 10 and 30 μM for 24 h, and the cell viability was assessed using MTT assay. All four compounds did not show any cytotoxic effects at 10 μM concentration (Figure 10). However, compound **8b** showed a significant reduction in cell viability at 30 μM (*p*-value = 0.003). Therefore, the protective effects of **8b** were evaluated on 6-OHDA- and rotenone-induced toxicity in PC12 cells up to the concentration of 10 μM. Meanwhile compounds **7b**, **8a**, and **8e** were tested up to the concentration of 30 μM.

#### 3.7.2. Protective Effect against 6-OHDA-Induced Cytotoxicity in PC12 Cells

**Figure 11:**
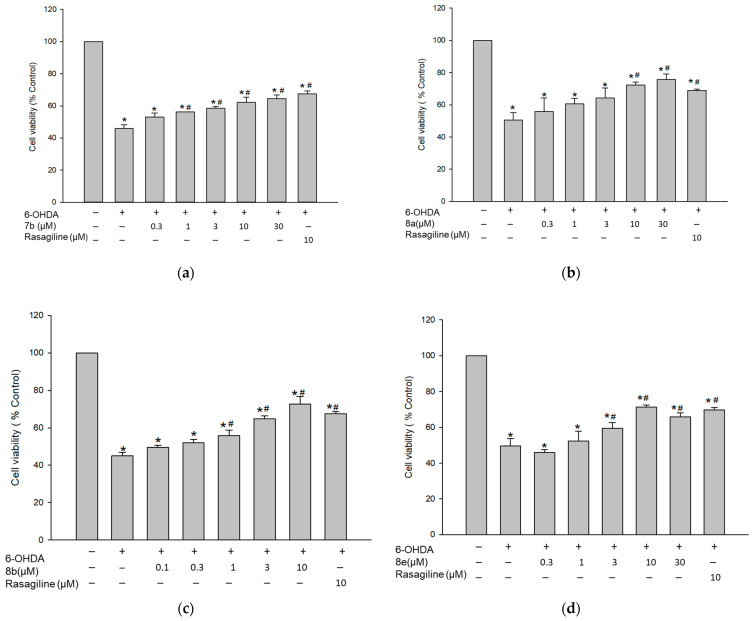
Inhibition of 6-OHDA-induced cytotoxicity by the test compounds in PC12 cells. PC12 cells were pre-treated for 4 h with compound **7b** (**a**), **8a** (**b**), **8b** (**c**), and **8e** (**d**) or rasagiline at the indicated concentrations and subsequently exposed to 6-OHDA (50 μM) for an additional 24 h. Cell viability was evaluated by MTT assay as described in the Materials and methods. Data are expressed as the mean ± SEM of at least three independent experiments. * *p* < 0.05 and # *p* < 0.05 vs. the vehicle-treated control and 6-OHDA-treated cells, respectively. 6-OHDA, 6-hydroxydopamine.

6-OHDA-induced cell-based model is a widely used model for evaluating the neuroprotective potential of substances [35,36,37,38,39]. After cellular absorption, 6-OHDA causes dopaminergic neurotoxicity by generating reactive oxygen species (ROS), which contributes to cell death. As a result, the protective effects of the test compounds on the 6-OHDA-induced cytotoxicity in PC12 cells were assessed using the MTT assay. The viability of cells incubated with 50 μM of 6-OHDA was reduced to 47.5 ± 1.2%, compared to that of control cells (Figure 11). Pretreatment with the test compounds at various concentrations for 4 h significantly attenuated the cytotoxicity induced by 6-OHDA (Figure 11). The decreased cell viability by 6-OHDA was maximally increased by compounds **7b** and **8a** to 64.5 ± 2.1 and 75.8 ± 3.4%, respectively, at 30 μM concentration. Meanwhile, **8b** and **8e** exerted their maximal protective effects at 10 μM concentration, raising the cell viability up to 72.6 ± 4.1 and 71.3 ± 1.2%, respectively. Rasagiline also increased the cell viability up to 67.9 ± 2.8% at the same concentration. These findings indicate that compounds **7b**, **8a**, **8b**, and **8e** exhibit protective effects against 6-OHDA-induced cytotoxicity in PC12 cells. Their maximal protective effects were comparable or slightly better than that of rasagiline (Figure 11).

#### 3.7.3. Protective Effect against Rotenone-Induced Cytotoxicity in PC12 Cells

**Figure 12:**
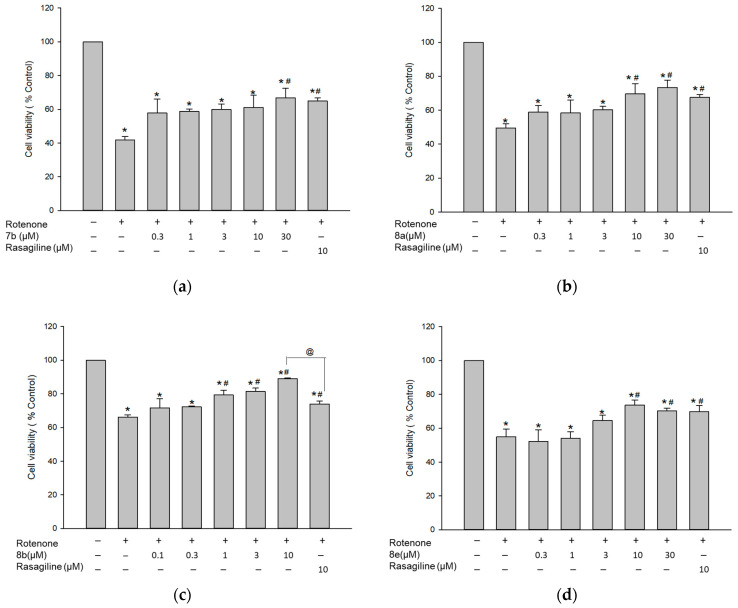
Inhibition of rotenone-induced cytotoxicity by the test compounds in PC12 cells. PC12 cells were pre-treated for 4 h with compound **7b** (**a**), **8a** (**b**), **8b** (**c**), and **8e** (**d**) or rasagiline at the indicated concentrations and subsequently exposed to rotenone (1 μM) for an additional 24 h. Cell viability was evaluated by MTT assay as described in the Materials and methods. Data are expressed as the mean ± SEM of at least three independent experiments. * *p* < 0.05, @ *p* < 0.05, and # *p* < 0.05 vs. the vehicle-treated control, rasagiline-treated, and rotenone-treated cells, respectively.

To verify the above-mentioned findings, we next evaluated the effects of compounds **7b**, **8a**, **8b**, and **8e** on the toxicity induced by rotenone, a well-known mitochondrial complex I inhibitor. Rotenone was shown to simulate the pathophysiology of PD [35,40,41,42,43]. As shown in Figure 12, exposure of PC12 cells to 1 μM of rotenone for 24 h induced 53.1 ± 2.9% cell death, compared to the vehicle-treated control cells. Pre-treatment with **7b** and **8a** markedly rescued cells from the rotenone-mediated damage, showing maximal cell viability of 66.8 ± 5.5 and 73.4 ± 4.2%, respectively, at 30 μM concentration. Furthermore, compounds **8b** and **8e** elevated the cell viability up to 89.0 ± 0.3 and 73.6 ± 3.1% at a concentration of 10 μM. Rasagiline also increased the cell viability to 69.8 ± 3.5% at 10 μM. These results demonstrate that compounds **7b**, **8a**, **8b**, and **8e** exhibit protective effects against rotenone-induced cytotoxicity in PC12 cells. While the maximal protective effects of compounds **7b**, **8a**, and **8e** were comparable to that of rasagiline, **8b** exhibited a significantly superior maximal effect to rasagiline (Figure 12c). Based on our findings, these compounds may be promising candidates for the development of new drugs to treat PD.

#### 3.7.4. Effect on 6-OHDA- or Rotenone-Induced ROS Generation in PC12 Cells

**Figure 13:**
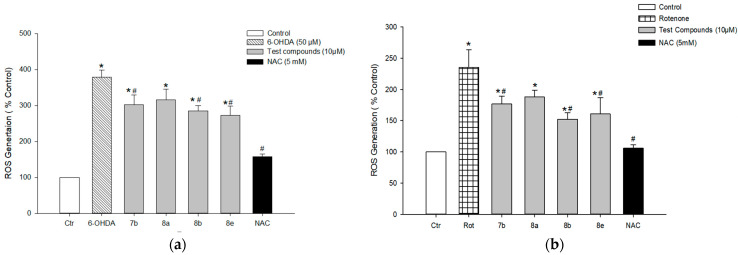
Effects of the test compounds on 6-OHDA- and rotenone-induced ROS generation in PC12 cells. PC12 cells were pre-incubated with 10 μM H_2_-DCFDA for 30 min at 37 °C in the dark and then treated with test compounds (10 μM) or NAC (5 mM) for 4 h, followed by exposure to 50 μM of 6-OHDA (**a**) or 1 μM of rotenone (**b**) for additional 20 h. The intracellular ROS levels were measured by the fluorescence detection of 2′,7′-dichlorofluorescein, as described in the Materials and methods. The ROS levels are expressed as percentages of those in the vehicle-treated control cells. Each data point represents the mean ± S.E.M. from three independent experiments conducted in triplicate. * *p* < 0.05 and # *p* < 0.05 vs. the vehicle-treated control and 6-OHDA- or rotenone-treated cells, respectively. 6-OHDA, 6-hydroxydopamine; Rot, rotenone; NAC, N-acetyl cysteine.

Oxidative stress is believed to be an important precursor of dopaminergic cell damage in PD. Thus, we next assessed the effects of the test compounds on the production of ROS using the cumulative fluorescent dye H_2_-DCFDA in PC12 cells exposed to 6-OHDA (50 μM) or rotenone (1 μM). Exposure of cells to 6-OHDA for 20 h markedly increased ROS generation, showing approximately 4-fold of control cells. Compounds **7b**, **8b**, and **8e** significantly decreased 6-OHDA-induced ROS generation at the concentration of 10 μM (Figure 13a). At the same concentration, compounds **7b**, **8b**, and **8e** attenuated rotenone-induced intracellular ROS production (Figure 13b). Meanwhile, compound **8a** was not effective to inhibit ROS generation in both 6-OHDA- and rotenone-treated cells. NAC was tested as a positive reference drug, which exerted dramatic inhibition of ROS at the concentration tested (5 mM).

#### 3.7.5. DPPH Radical Scavenging Activity

**Figure 14:**
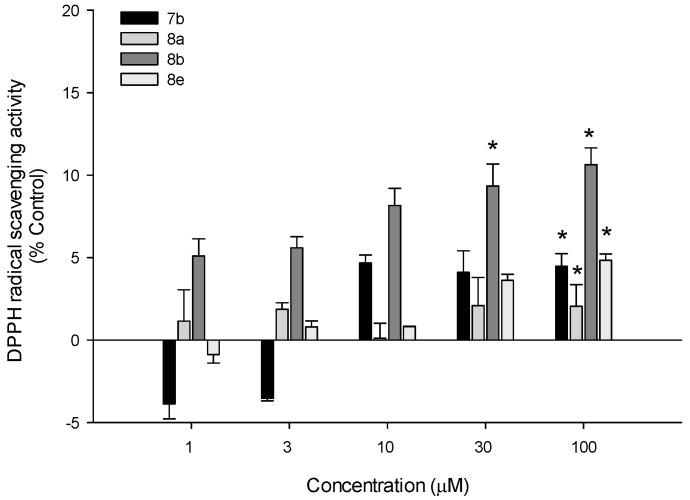
DPPH radical scavenging activities of the test compounds. A cell-free bioassay was performed as described in the Materials and methods to assess DPPH radical scavenging activities of compounds **7b**, **8a**, **8b**, and **8e** at the indicated concentrations. Data are expressed as the mean ± SEM of three independent experiments. * *p* < 0.05 vs. vehicle-treated control.

The antioxidant potentials of test compounds were further explored by assessing their abilities to scavenge DPPH radicals in a cell-free assay. We found that all four compounds exhibited mild DPPH radical scavenging activities, with the maximal scavenging effect of approximately 10% by compound **8b** at the concentrations of 30 and 100 µM (Figure 14). Compound **8b** with the most active radical scavenging activity was also shown to be more active against ROS in PC12 cells (Figure 13). Based on these findings, radical scavenging activities of the test compounds may contribute, at least in part, to inhibiting intracellular ROS induced by 6-OHDA or rotenone in PC12 cells. Further studies are required to clarify whether the test compounds can activate the antioxidant systems of PC12 cells.

Based on our data, compound **8b** appeared to exert the most potent protective effects against 6-OHDA- and rotenone-induced oxidative cytotoxicity in PC12 cells. The protective effect of **8b** against rotenone toxicity was superior to that of rasagiline (*p* < 0.001) at the same concentration (10 μM). While in 6-OHDA-induced cell damage, protective effects of **8b** and rasagiline were not statically different (*p* = 0.534). Using the concentration-dependent effects of **8b** against 6-OHDA- and rotenone-induced cytotoxicity, the calculated IC_50_ values were 2.8 and 3.3 μM, respectively. The IC_50_ values of the remaining compounds could not be determined since the maximal effects of these compounds were close to or below 50%. In addition, compound **8b** was shown to attenuate 6-OHDA- or rotenone-induced ROS generation in PC12 cells with mild DPPH radical scavenging activity. Although compound **8b** appeared to be the most potent, its protective effect was markedly decreased as we increased the concentration to 30 μM. Therefore, the safety profile of compound **8b** as well as its neuroprotective property need to be improved by further chemical modifications. Moreover, it would be valuable to further confirm our findings in primary cultures of dopaminergic neurons using various biological assays specifically measuring cell proliferation or cell death.

## 4. Conclusions

In this study, a new series of indole-based compounds was identified as potential MAO-B inhibitors. Compounds **7b**, **8a**, **8b**, and **8e** exhibited MAO-B IC_50_ values of 0.33, 0.02, 0.03, and 0.45 µM with remarkable selectivity indices compared to their activity over MAO-A (SI > 305, 3649, 3278, and 220, respectively). Compounds **8a** and **8b** showed a competitive and reversible mode of action with Ki values of 10.34 and 6.63 nM over MAO-B. The docking simulation revealed that **7b** and **8a** form strong H-bonding interactions with the water molecule HOH1247, as well as H-bonds and π-π interactions with Tyr326 and numerous hydrophobic interactions, which favor the compounds’ activity. 3,4-dichlorophenyl derivatives are more active than alkyl substituents due to favorable interactions with the “aromatic cage” of MAO-B. The remarkable selectivity of the compounds occurs because of the inability of the indole moiety to form direct or water-mediated H-bonds or π-π interactions with the residues close to FAD in MAO-A. Furthermore, compounds **7b**, **8a**, **8b**, and **8e** were found to possess safe neurotoxicity profiles in PC12 cells and significantly attenuated 6-OHDA- and rotenone-induced oxidative damage. Compounds **7b**, **8b**, and **8e** showed mild DPPH radical scavenging activities and significantly attenuated 6-OHDA- and rotenone-induced intracellular ROS production in PC12 cells. Accordingly, the findings of our study could be a milestone for further development of the indole-based small molecules as multi-targeted promising leads in the fight against oxidative stress-related PD.

## Figures and Tables

**Table 1:** Inhibitory effects of the prepared compounds against MAO-B enzyme.

Comp.	C5 Linker	R (C5 Substitution)	N1 Indole Substitution	% Inhibition of MAO-B at 10 µM
**4a**	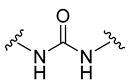	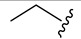	3-Fluorobenzoyl	46.2 ± 1.1
**4b**		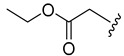		9.9 ± 1.5
**4c**		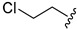		26.6 ± 0.6
**4d**		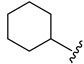		44.0 ± 0.8
**4e**		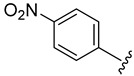		47.9 ± 1.6
**4f**		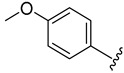		32.5 ± 1.4
**4g**		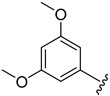		55.7 ± 0.2
**4h**		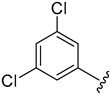		38.6 ± 1.6
**4i**		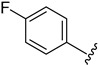		37.0 ± 0.9
**4j**		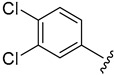		53.5 ± 0.3
**4k**		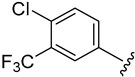		42.1 ± 1.7
**4l**		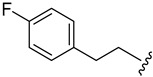		42.0 ± 0.6
**4m**		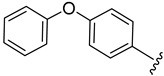		38.1 ± 0.5
**4n**		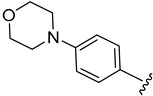		32.9 ± 0.3
**5a**	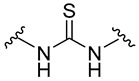	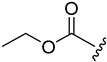	3-Fluorobenzoyl	15.5 ± 1.1
**5b**		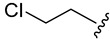		45.6 ± 1.2
**5c**		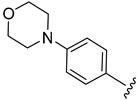		43.9 ± 1.1
**5d**		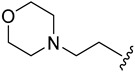		46.8 ± 0.2
**5e**		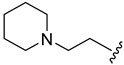		44.7 ± 0.5
**5f**		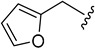		34.1 ± 0.7
**5g**		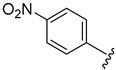		30.6 ± 0.9
**5h**		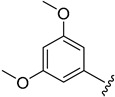		32.5 ± 0.5
**5i**		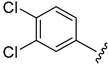		59.8 ± 0.4
**5j**		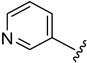		51.1 ± 0.4
**6a**	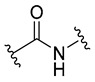	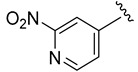	3-Fluorobenzoyl	26.8 ± 1.3
**6b**		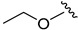		33.8 ± 2.3
**6c**		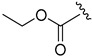		35.5 ± 1.5
**6d**		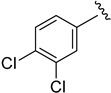		53.0 ± 0.3
**6e**		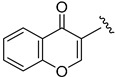		59.6 ± 0.3
**7a**	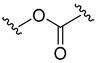	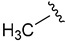	3-Fluorobenzoyl	49.8 ± 0.3
**7b**		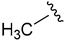	3,4-Dichlorobenzoyl	84.1 ± 0.0
**8a**	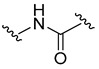	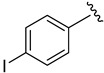	Free NH	99.3 ± 0.0
**8b**		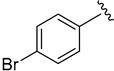		99.4 ± 0.0
**8c**		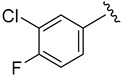		34.2 ± 0.2
**8d**		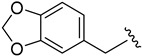		15.3 ± 0.8
**8e**		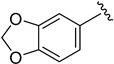		89.6 ± 0.4

**Table 2:** Inhibitory effects against MAO-A and the selectivity indices.

	MAO-A								
Cpd	Inhibition% (100 µM)	S.E.M	Inhibition% (30 µM)	S.E.M	Inhibition% (10 µM)	S.E.M	MAO-A IC_50_ (µM)	MAO-B IC_50_ (µM)	Selectivity Index (SI) ^a^
**7b**	<10	2.789	<10	1.449	<10	0.334	>100	0.3278	>305
**8a**	23.48	21.66	21.66	0.920	17.16	0.687	>100	0.0274	>3649
**8b**	30.07	1.474	27.88	0.965	15.07	0.764	>100	0.0305	>3278
**8e**	<10	1.959	<10	1.009	<10	0.689	>100	0.4532	>220

^a^ SI = selectivity index, the selectivity for the MAO-B isoform and is given as the ratio of IC_50_ (MAO-A)/IC_50_ (MAO-B).

## Data Availability

All of the data are contained within the article and the Supplementary Materials.

## Supplementary Materials

The following are available online at https://www.mdpi.com/article/10.3390/antiox10101641/s1, Figure S1. ^1^HNMR, ^13^CNMR, HPLC and HRMS data of the compounds reported in this study, in addition to binding modes of reference reversible inhibitors.
