# Supplementary material for: Highly Potent, Selective, and Competitive Indole-Based MAO-B Inhibitors Protect PC12 Cells against 6-Hydroxydopamine- and Rotenone-Induced Oxidative Stress
Source: Antioxidants (Basel). 2021 Oct 19;10(10):1641. doi: 10.3390/antiox10101641 (PMC8533206; doi:10.3390/antiox10101641)
Supplement: Supplementary file 1 [file antioxidants-10-01641-s001.zip › antioxidants-1378980-supplementary.pdf]

Supplementary material

Highly Potent, Selective, and Competitive Indole-Based MAO-B Inhibitors Protect PC12 Cells against 6-Hydroxydopamine- and Rotenone-Induced Oxidative Stress

Contents

1. ^{1}HNMR and ^{13}CNMR data of compounds **4a**−**n**, **5a**−**j**, **6a**−**e**, **7a**, **7b**, and **8a**−**e**
2. HPLC purity data
3. HRMS data
4. Molecular modelling supplementary material

1. **^{1}HNMR and ^{13}CNMR data of compounds 4a−n, 5a−j, 6a−e, 7a, 7b,** and **8a−e**

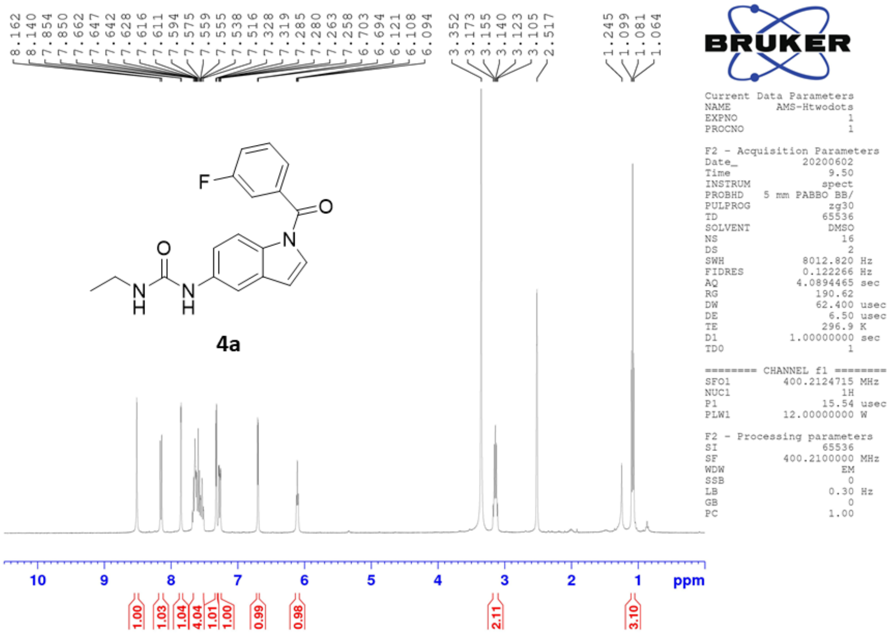

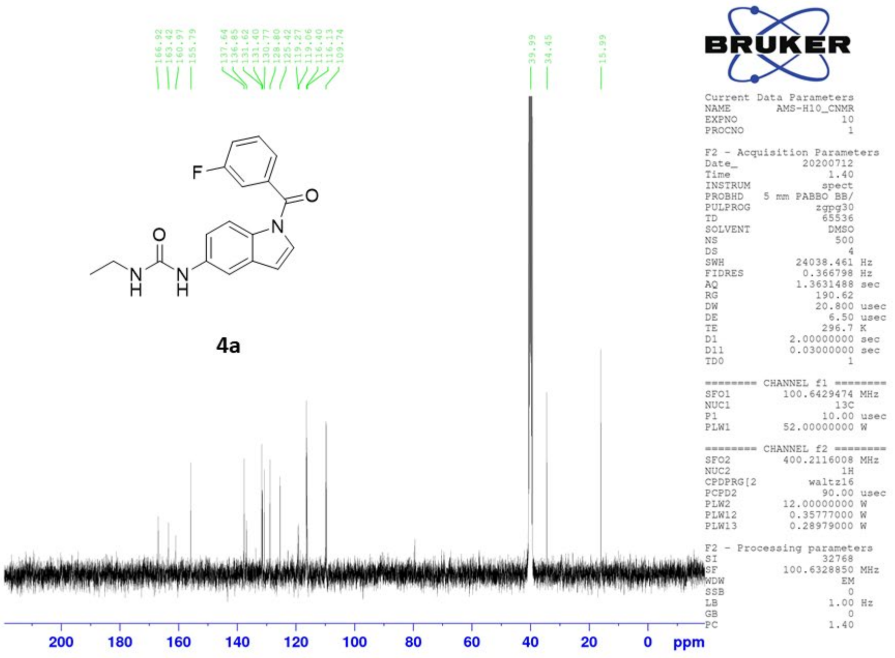

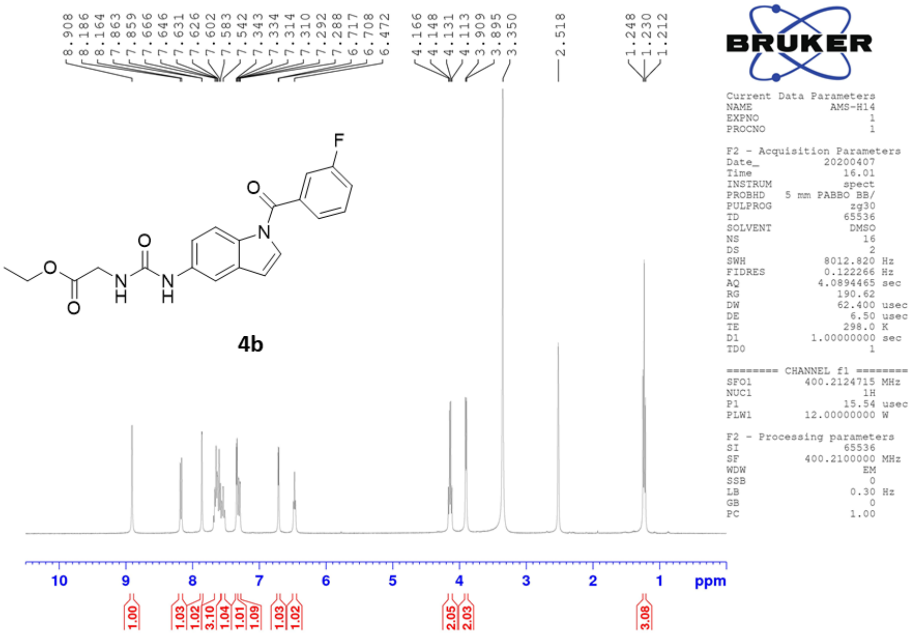
8.908
8.186
8.164
7.863
7.859
7.666
7.646
7.631
7.626
7.602
7.583
7.542
7.343
7.334
7.314
7.310
7.292
7.288
6.717
6.708
6.472
4.166
4.148
4.131
4.113
3.909
3.895
3.350
2.518
1.248
1.230
1.212
F
O
N
O
O
N
H
N
H
O
BRUKER
Current Data Parameters
NAME AMS-H14
EXPNO 1
PROCNO 1
F2 - Acquisition Parameters
Date_ 20200407
Time 16.01
INSTRUM spect
PROBHD 5 mm PABBO BB/
PULPROG zg30
TD 65536
SOLVENT DMSO
NS 16
DS 2
SWH 8012.820 Hz
FIDRES 0.122266 Hz
AQ 4.0894465 sec
RG 190.62
DW 62.400 usec
DE 6.50 usec
TE 298.0 K
D1 1.00000000 sec
TD0 1
======== CHANNEL f1 ========
SFO1 400.2124715 MHz
NUC1 1H
P1 15.54 usec
PLW1 12.00000000 W
F2 - Processing parameters
SI 65536
SF 400.2100000 MHz
WDW EM
SSB 0
LB 0.30 Hz
GB 0
PC 1.00
10
9
8
7
6
5
4
3
2
1
ppm
1.00
1.03
1.02
3.10
1.04
1.01
1.09
1.03
1.02
2.05
2.03
3.08

4b

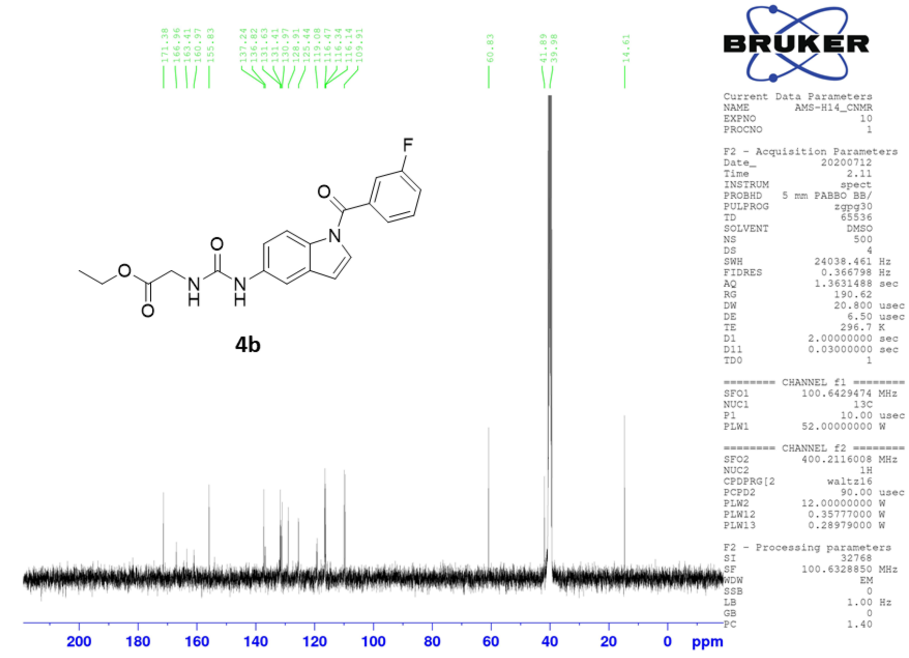
171.38
166.96
163.41
160.97
155.83
137.24
136.82
131.63
131.41
130.97
128.91
125.44
119.68
116.47
116.34
116.14
109.91
60.83
41.89
39.98
14.61
F
O
N
O
O
N
H
N
H
O
BRUKER
Current Data Parameters
NAME AMS-H14_CNMR
EXPNO 10
PROCNO 1
F2 - Acquisition Parameters
Date_ 20200712
Time 2.11
INSTRUM spect
PROBHD 5 mm PABBO BB/
PULPROG zgpg30
TD 65536
SOLVENT DMSO
NS 500
DS 4
SWH 24038.461 Hz
FIDRES 0.366798 Hz
AQ 1.3631488 sec
RG 190.62
DW 20.800 usec
DE 6.50 usec
TE 296.7 K
D1 2.00000000 sec
D11 0.03000000 sec
TD0 1
======== CHANNEL f1 ========
SFO1 100.6429474 MHz
NUC1 13C
P1 10.00 usec
PLW1 52.00000000 W
======== CHANNEL f2 ========
SFO2 400.2116008 MHz
NUC2 1H
CPDPRG[2 waltz16
PCPD2 90.00 usec
PLW2 12.00000000 W
PLW12 0.35777000 W
PLW13 0.28979000 W
F2 - Processing parameters
SI 32768
SF 100.6328850 MHz
WDW EM
SSB 0
LB 1.00 Hz
GB 0
PC 1.40
200
180
160
140
120
100
80
60
40
20
0
ppm

4b

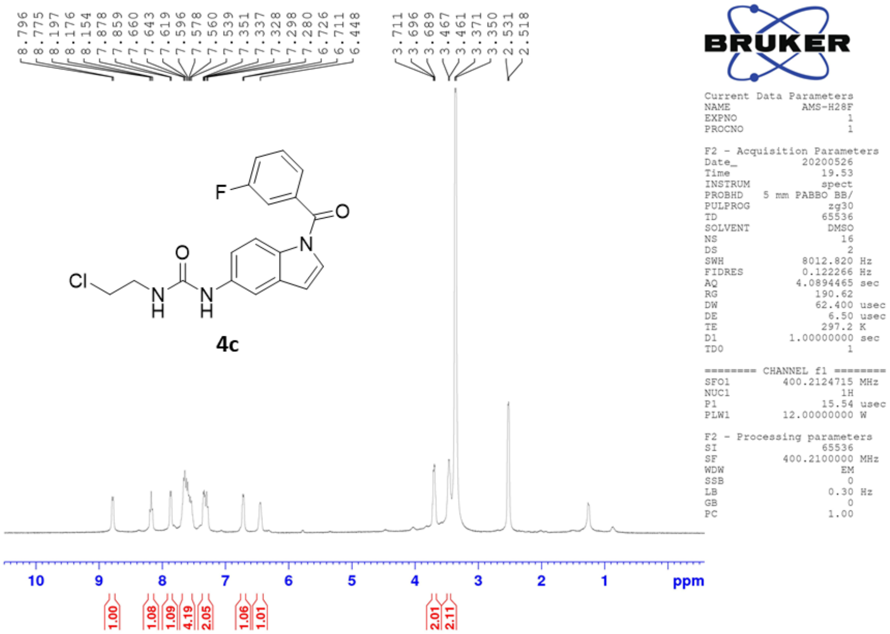
8.796 8.775 8.197 8.176 8.154 7.878 7.859 7.660 7.643 7.619 7.596 7.578 7.560 7.539 7.351 7.337 7.328 7.298 7.280 6.726 6.711 6.448
3.711 3.696 3.689 3.467 3.461 3.371 3.350 2.531 2.518
F
O
Cl
N
H
4c
BRUKER
Current Data Parameters
NAME AMS-H28F
EXPNO 1
PROCNO 1
F2 - Acquisition Parameters
Date_ 20200526
Time 19.53
INSTRUM spect
PROBHD 5 mm PABBO BB/
PULPROG zg30
TD 65536
SOLVENT DMSO
NS 16
DS 2
SWH 8012.820 Hz
FIDRES 0.122266 Hz
AQ 4.0894465 sec
RG 190.62
DW 62.400 usec
DE 6.50 usec
TE 297.2 K
D1 1.00000000 sec
TD0 1
======== CHANNEL f1 ========
SFO1 400.2124715 MHz
NUC1 1H
P1 15.54 usec
PLW1 12.00000000 W
F2 - Processing parameters
SI 65536
SF 400.2100000 MHz
WDW EM
SSB 0
LB 0.30 Hz
GB 0
PC 1.00
10 9 8 7 6 5 4 3 2 1 ppm
1.00 1.08 1.09 4.19 2.05 1.06 1.01 2.01 2.11

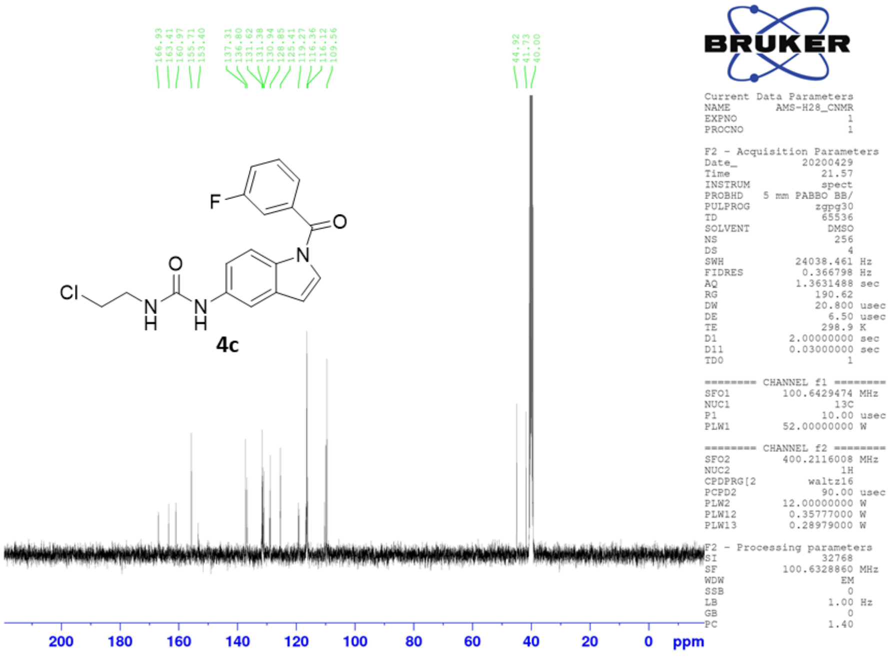
166.93 163.41 160.97 155.71 153.40 137.31 136.80 131.62 131.38 130.94 128.85 125.41 119.27 116.36 116.12 109.56
44.92 41.73 40.00
F
O
Cl
N
H
4c
BRUKER
Current Data Parameters
NAME AMS-H28_CNMR
EXPNO 1
PROCNO 1
F2 - Acquisition Parameters
Date_ 20200429
Time 21.57
INSTRUM spect
PROBHD 5 mm PABBO BB/
PULPROG zgpg30
TD 65536
SOLVENT DMSO
NS 256
DS 4
SWH 24038.461 Hz
FIDRES 0.366798 Hz
AQ 1.3631488 sec
RG 190.62
DW 20.800 usec
DE 6.50 usec
TE 298.9 K
D1 2.00000000 sec
D11 0.03000000 sec
TD0 1
======== CHANNEL f1 ========
SFO1 100.6429474 MHz
NUC1 13C
P1 10.00 usec
PLW1 52.00000000 W
======== CHANNEL f2 ========
SFO2 400.2116008 MHz
NUC2 1H
CPDPRG[2 waltz16
PCPD2 90.00 usec
PLW2 12.00000000 W
PLW12 0.35777000 W
PLW13 0.28979000 W
F2 - Processing parameters
SI 32768
SF 100.6328860 MHz
WDW EM
SSB 0
LB 1.00 Hz
GB 0
PC 1.40
200 180 160 140 120 100 80 60 40 20 0 ppm

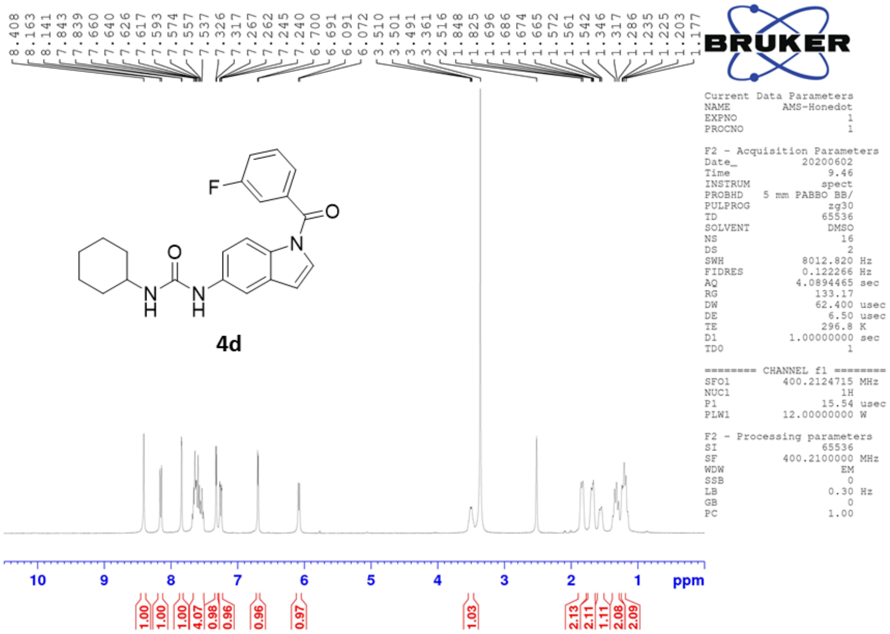
8.408
8.163
8.141
7.843
7.839
7.660
7.640
7.626
7.617
7.593
7.574
7.557
7.537
7.326
7.317
7.267
7.262
7.245
7.240
6.700
6.691
6.091
6.072
3.510
3.501
3.491
3.361
2.516
1.848
1.825
1.696
1.686
1.674
1.665
1.572
1.561
1.542
1.346
1.317
1.286
1.235
1.225
1.203
1.177
F
O
N
O
N
H
N
H
ppm
1.00
1.00
1.00
4.07
0.98
0.96
0.96
0.97
1.03
2.13
2.11
1.11
2.08
2.09
Current Data Parameters
NAME AMS-Honedot
EXPNO 1
PROCNO 1
F2 - Acquisition Parameters
Date_ 20200602
Time 9.46
INSTRUM spect
PROBHD 5 mm PABBO BB/
PULPROG zg30
TD 65536
SOLVENT DMSO
NS 16
DS 2
SWH 8012.820 Hz
FIDRES 0.122266 Hz
AQ 4.0894465 sec
RG 133.17
DW 62.400 usec
DE 6.50 usec
TE 296.8 K
D1 1.00000000 sec
TD0 1
======== CHANNEL f1 ========
SFO1 400.2124715 MHz
NUC1 1H
P1 15.54 usec
PLW1 12.00000000 W
F2 - Processing parameters
SI 65536
SF 400.2100000 MHz
WDW EM
SSB 0
LB 0.30 Hz
GB 0
PC 1.00

4d

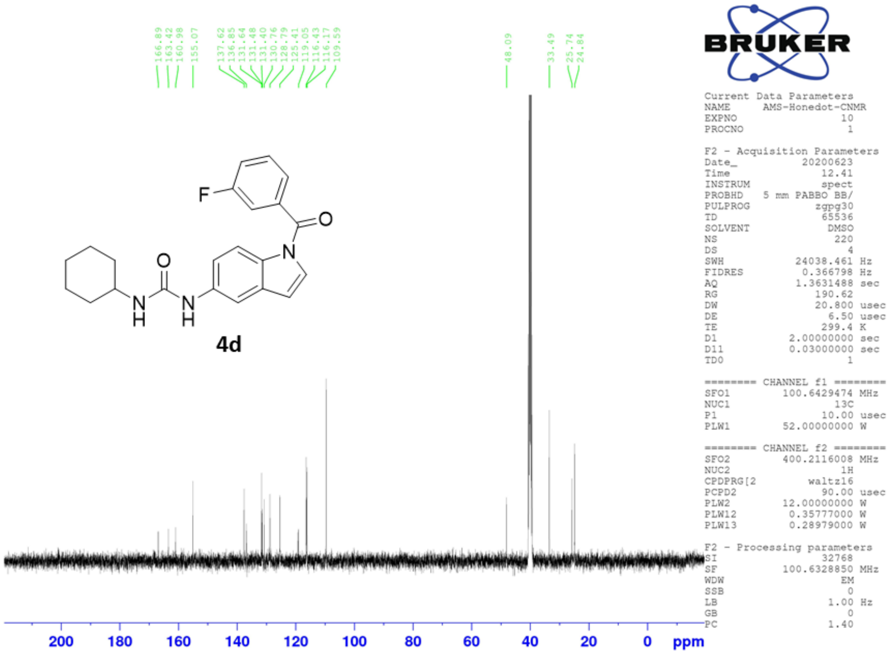
166.89
163.42
160.98
155.07
137.62
136.85
131.64
131.48
131.40
130.76
128.79
125.41
119.05
116.43
116.17
109.59
48.09
33.49
25.74
24.84
F
O
N
O
N
H
N
H
ppm
Current Data Parameters
NAME AMS-Honedot-CNMR
EXPNO 10
PROCNO 1
F2 - Acquisition Parameters
Date_ 20200623
Time 12.41
INSTRUM spect
PROBHD 5 mm PABBO BB/
PULPROG zgpg30
TD 65536
SOLVENT DMSO
NS 220
DS 4
SWH 24038.461 Hz
FIDRES 0.366798 Hz
AQ 1.3631488 sec
RG 190.62
DW 20.800 usec
DE 6.50 usec
TE 299.4 K
D1 2.00000000 sec
D11 0.03000000 sec
TD0 1
======== CHANNEL f1 ========
SFO1 100.6429474 MHz
NUC1 13C
P1 10.00 usec
PLW1 52.00000000 W
======== CHANNEL f2 ========
SFO2 400.2116008 MHz
NUC2 1H
CPDPRG[2 waltz16
PCPD2 90.00 usec
PLW2 12.00000000 W
PLW12 0.35777000 W
PLW13 0.28979000 W
F2 - Processing parameters
SI 32768
SF 100.6328850 MHz
WDW EM
SSB 0
LB 1.00 Hz
GB 0
PC 1.40

4d

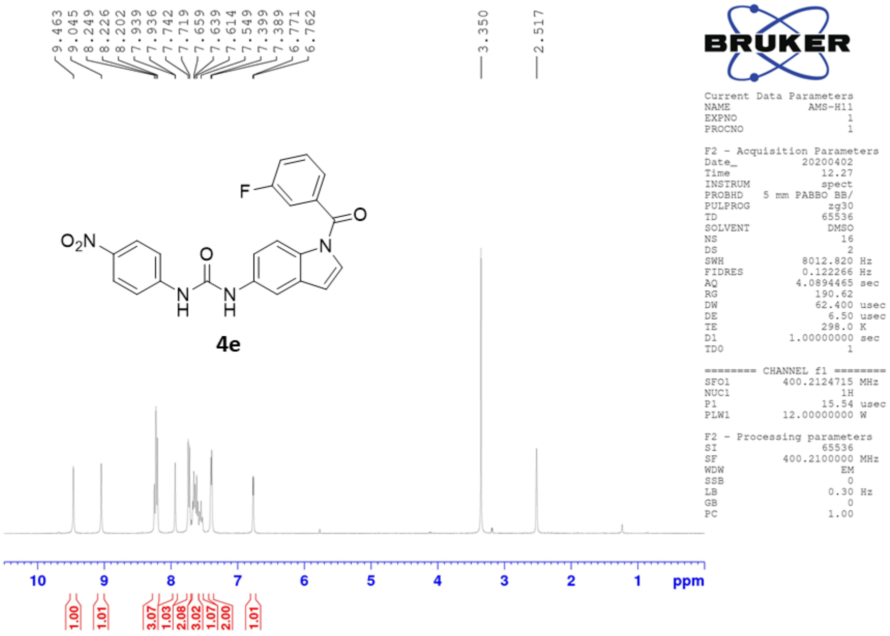
9.463
9.045
8.249
8.226
8.202
7.939
7.936
7.742
7.719
7.659
7.639
7.614
7.549
7.399
7.389
6.771
6.762
3.350
2.517
F
O
O_2N
N
H
4e
BRUKER
Current Data Parameters
NAME AMS-H11
EXPNO 1
PROCNO 1
F2 - Acquisition Parameters
Date_ 20200402
Time 12.27
INSTRUM spect
PROBHD 5 mm PABBO BB/
PULPROG zg30
TD 65536
SOLVENT DMSO
NS 16
DS 2
SWH 8012.820 Hz
FIDRES 0.122266 Hz
AQ 4.0894465 sec
RG 190.62
DW 62.400 usec
DE 6.50 usec
TE 298.0 K
D1 1.00000000 sec
TD0 1
======== CHANNEL f1 ========
SFO1 400.2124715 MHz
NUC1 1H
P1 15.54 usec
PLW1 12.00000000 W
F2 - Processing parameters
SI 65536
SF 400.2100000 MHz
WDW EM
SSB 0
LB 0.30 Hz
GB 0
PC 1.00
10
9
8
7
6
5
4
3
2
1
ppm
1.00
1.01
3.07
1.03
2.08
3.02
1.07
2.00
1.01

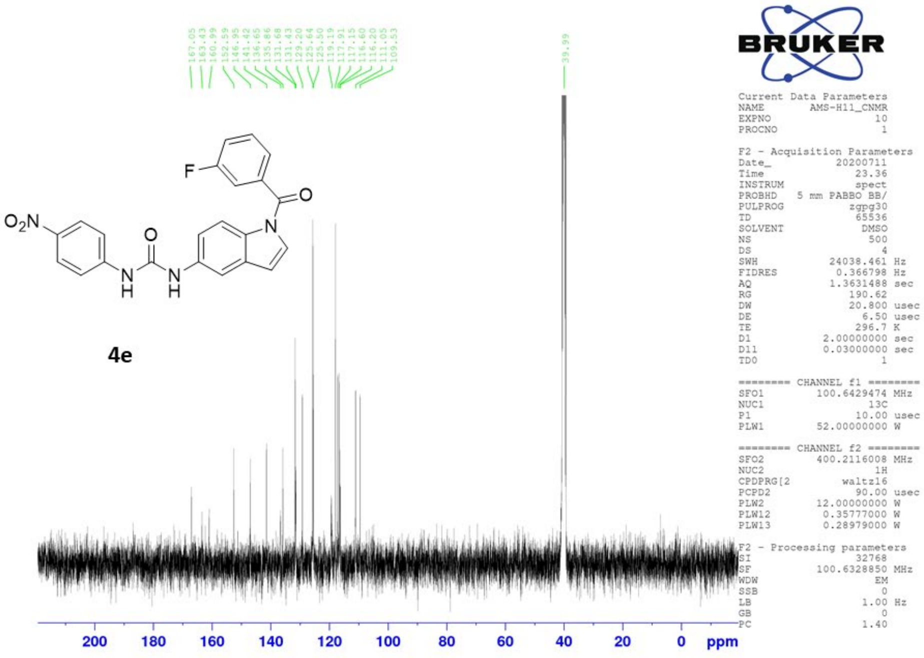
167.05
163.43
160.99
152.59
146.95
141.42
136.65
135.86
131.68
131.43
129.20
125.64
125.50
119.19
117.91
117.15
116.60
116.20
111.05
109.53
39.99
F
O
O_2N
N
H
4e
BRUKER
Current Data Parameters
NAME AMS-H11_CNMR
EXPNO 10
PROCNO 1
F2 - Acquisition Parameters
Date_ 20200711
Time 23.36
INSTRUM spect
PROBHD 5 mm PABBO BB/
PULPROG zgpg30
TD 65536
SOLVENT DMSO
NS 500
DS 4
SWH 24038.461 Hz
FIDRES 0.366798 Hz
AQ 1.3631488 sec
RG 190.62
DW 20.800 usec
DE 6.50 usec
TE 296.7 K
D1 2.00000000 sec
D11 0.03000000 sec
TD0 1
======== CHANNEL f1 ========
SFO1 100.6429474 MHz
NUC1 13C
P1 10.00 usec
PLW1 52.00000000 W
======== CHANNEL f2 ========
SFO2 400.2116008 MHz
NUC2 1H
CPDPRG[2 waltz16
PCPD2 90.00 usec
PLW2 12.00000000 W
PLW12 0.35777000 W
PLW13 0.28979000 W
F2 - Processing parameters
SI 32768
SF 100.6328850 MHz
WDW EM
SSB 0
LB 1.00 Hz
GB 0
PC 1.40
200
180
160
140
120
100
80
60
40
20
0
ppm

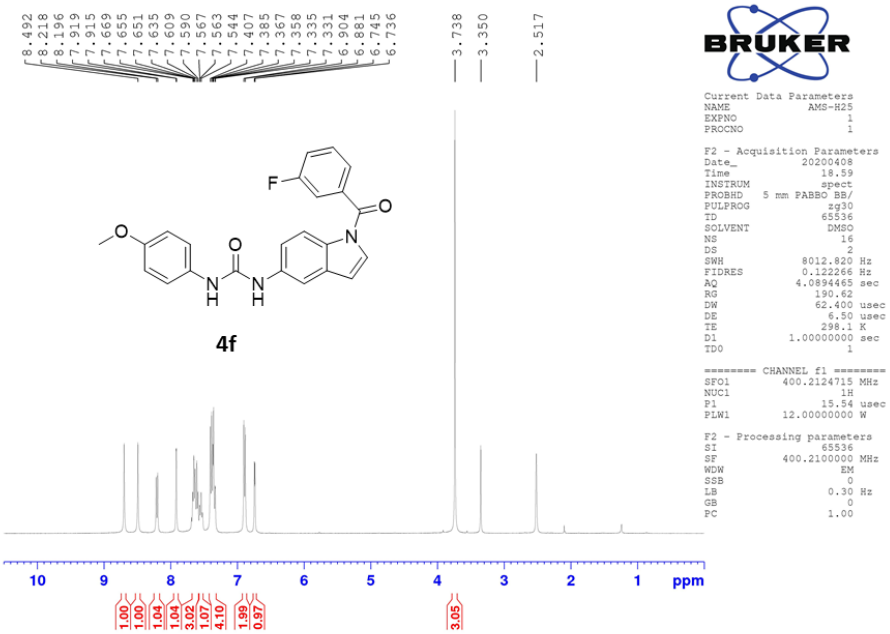
8.492
8.218
8.196
7.919
7.915
7.669
7.655
7.651
7.635
7.609
7.590
7.567
7.563
7.544
7.407
7.385
7.367
7.358
7.335
7.331
6.904
6.881
6.745
6.736
3.738
3.350
2.517
BRUKER
4f
1.00
1.00
1.04
1.04
3.02
1.07
4.10
1.99
0.97
3.05
ppm
Current Data Parameters
NAME AMS-H25
EXPNO 1
PROCNO 1
F2 - Acquisition Parameters
Date_ 20200408
Time 18.59
INSTRUM spect
PROBHD 5 mm PABBO BB/
PULPROG zg30
TD 65536
SOLVENT DMSO
NS 16
DS 2
SWH 8012.820 Hz
FIDRES 0.122266 Hz
AQ 4.0894465 sec
RG 190.62
DW 62.400 usec
DE 6.50 usec
TE 298.1 K
D1 1.00000000 sec
TD0 1
======== CHANNEL f1 ========
SFO1 400.2124715 MHz
NUC1 1H
P1 15.54 usec
PLW1 12.00000000 W
F2 - Processing parameters
SI 65536
SF 400.2100000 MHz
WDW EM
SSB 0
LB 0.30 Hz
GB 0
PC 1.00

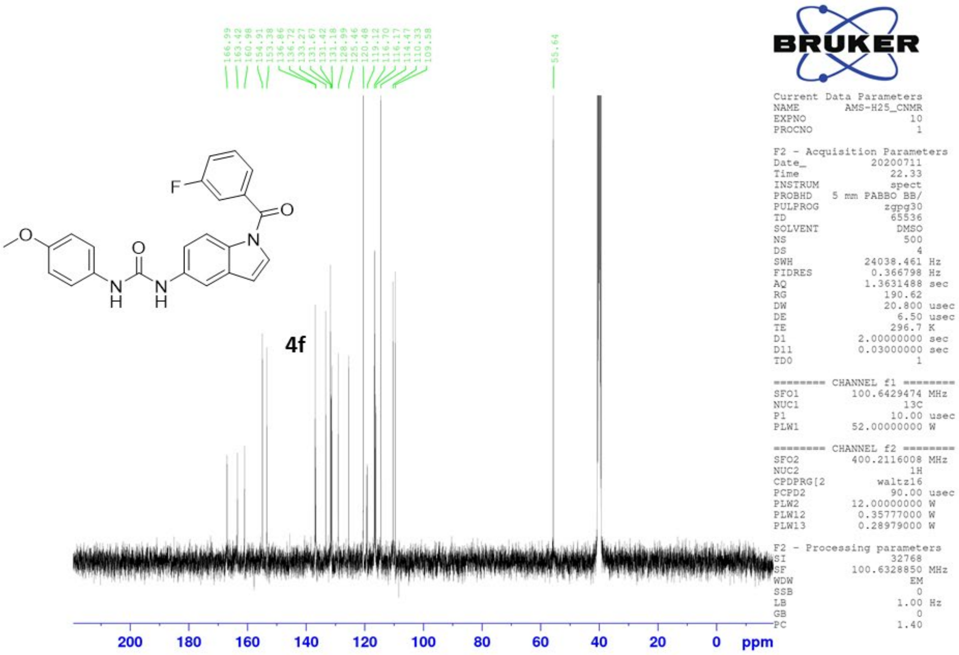
166.99
163.42
160.98
154.91
153.38
136.86
136.72
133.27
131.67
131.42
131.18
128.99
125.46
120.48
119.12
116.70
116.17
114.47
110.33
109.58
55.64
BRUKER
4f
ppm
Current Data Parameters
NAME AMS-H25_CNMR
EXPNO 10
PROCNO 1
F2 - Acquisition Parameters
Date_ 20200711
Time 22.33
INSTRUM spect
PROBHD 5 mm PABBO BB/
PULPROG zgpg30
TD 65536
SOLVENT DMSO
NS 500
DS 4
SWH 24038.461 Hz
FIDRES 0.366798 Hz
AQ 1.3631488 sec
RG 190.62
DW 20.800 usec
DE 6.50 usec
TE 296.7 K
D1 2.00000000 sec
D11 0.03000000 sec
TD0 1
======== CHANNEL f1 ========
SFO1 100.6429474 MHz
NUC1 13C
P1 10.00 usec
PLW1 52.00000000 W
======== CHANNEL f2 ========
SFO2 400.2116008 MHz
NUC2 1H
CPDPRG[2 waltz16
PCPD2 90.00 usec
PLW2 12.00000000 W
PLW12 0.35777000 W
PLW13 0.28979000 W
F2 - Processing parameters
SI 32768
SF 100.6328850 MHz
WDW EM
SSB 0
LB 1.00 Hz
GB 0
PC 1.40

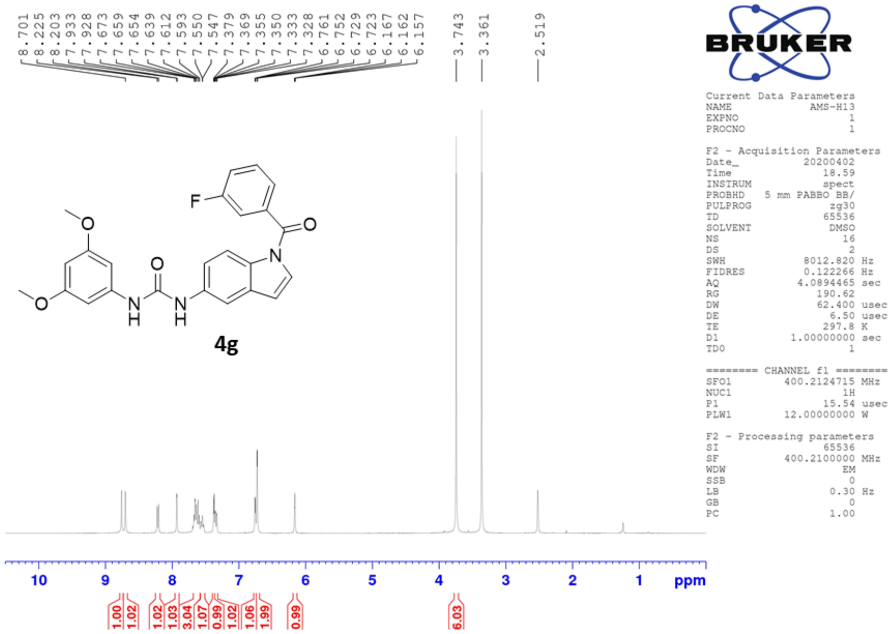
8.701 8.225 8.203 7.933 7.928 7.673 7.659 7.654 7.639 7.612 7.593 7.550 7.547 7.379 7.369 7.355 7.350 7.333 7.328 6.761 6.752 6.729 6.729 6.723 6.167 6.162 6.157
3.743
3.361
2.519
F
O
N
O
O
H
N
H
N
O
4g
BRUKER
Current Data Parameters
NAME AMS-H13
EXPNO 1
PROCNO 1
F2 - Acquisition Parameters
Date_ 20200402
Time 18.59
INSTRUM spect
PROBHD 5 mm PABBO BB/
PULPROG zg30
TD 65536
SOLVENT DMSO
NS 16
DS 2
SWH 8012.820 Hz
FIDRES 0.122266 Hz
AQ 4.0894465 sec
RG 190.62
DW 62.400 usec
DE 6.50 usec
TE 297.8 K
D1 1.00000000 sec
TD0 1
======== CHANNEL f1 ========
SFO1 400.2124715 MHz
NUC1 1H
P1 15.54 usec
PLW1 12.00000000 W
F2 - Processing parameters
SI 65536
SF 400.2100000 MHz
WDW EM
SSB 0
LB 0.30 Hz
GB 0
PC 1.00
10 9 8 7 6 5 4 3 2 1 ppm
1.00 1.02 1.02 1.03 3.04 1.07 0.99 1.02 1.06 1.99 0.99 6.03

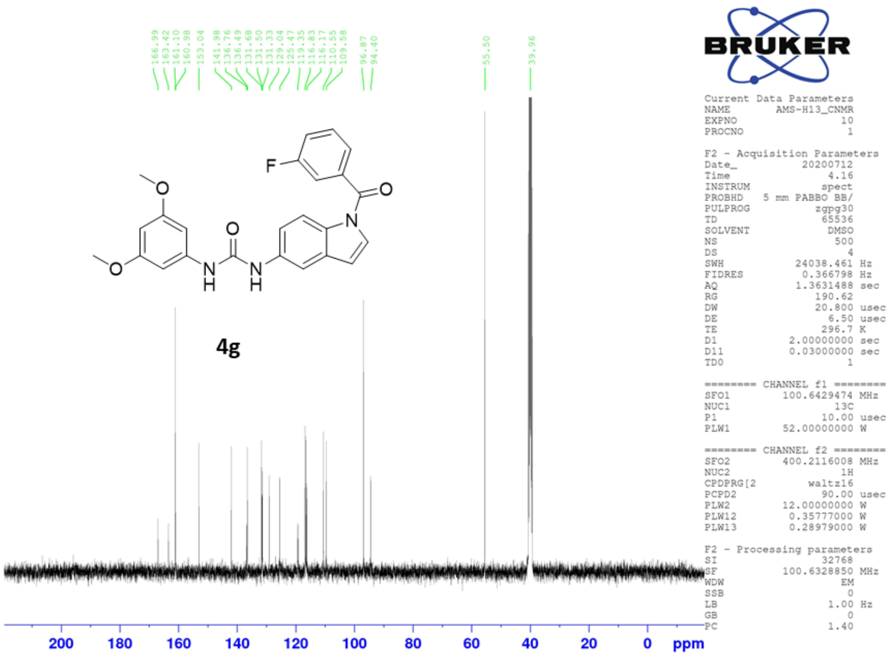
166.99 163.42 161.10 160.98 153.04 141.98 136.76 136.49 131.68 131.50 131.33 129.04 125.47 119.35 116.83 116.17 110.55 109.58 96.87 94.40
55.50
39.96
F
O
N
O
O
H
N
H
N
O
4g
BRUKER
Current Data Parameters
NAME AMS-H13_CNMR
EXPNO 10
PROCNO 1
F2 - Acquisition Parameters
Date_ 20200712
Time 4.16
INSTRUM spect
PROBHD 5 mm PABBO BB/
PULPROG zgpg30
TD 65536
SOLVENT DMSO
NS 500
DS 4
SWH 24038.461 Hz
FIDRES 0.366798 Hz
AQ 1.3631488 sec
RG 190.62
DW 20.800 usec
DE 6.50 usec
TE 296.7 K
D1 2.00000000 sec
D11 0.03000000 sec
TD0 1
======== CHANNEL f1 ========
SFO1 100.6429474 MHz
NUC1 13C
P1 10.00 usec
PLW1 52.00000000 W
======== CHANNEL f2 ========
SFO2 400.2116008 MHz
NUC2 1H
CPDPRG[2 waltz16
PCPD2 90.00 usec
PLW2 12.00000000 W
PLW12 0.35777000 W
PLW13 0.28979000 W
F2 - Processing parameters
SI 32768
SF 100.6328850 MHz
WDW EM
SSB 0
LB 1.00 Hz
GB 0
PC 1.40
200 180 160 140 120 100 80 60 40 20 0 ppm

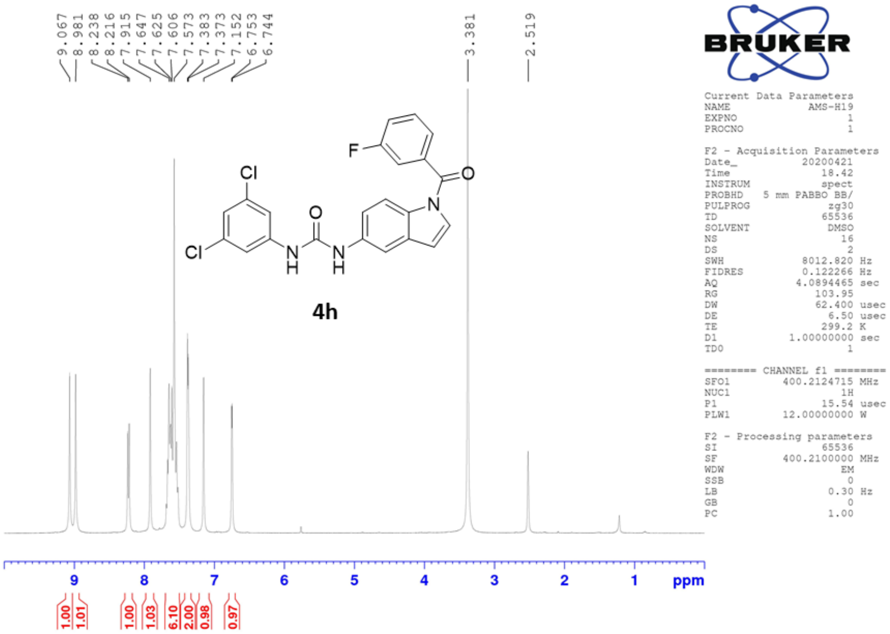
9.067
8.981
8.238
8.216
7.915
7.647
7.625
7.606
7.573
7.383
7.373
7.152
6.753
6.744
3.381
2.519
BRUKER
Current Data Parameters
NAME AMS-H19
EXPNO 1
PROCNO 1
F2 - Acquisition Parameters
Date_ 20200421
Time 18.42
INSTRUM spect
PROBHD 5 mm PABBO BB/
PULPROG zg30
TD 65536
SOLVENT DMSO
NS 16
DS 2
SWH 8012.820 Hz
FIDRES 0.122266 Hz
AQ 4.0894465 sec
RG 103.95
DW 62.400 usec
DE 6.50 usec
TE 299.2 K
D1 1.00000000 sec
TD0 1
======== CHANNEL f1 ========
SFO1 400.2124715 MHz
NUC1 1H
P1 15.54 usec
PLW1 12.00000000 W
F2 - Processing parameters
SI 65536
SF 400.2100000 MHz
WDW EM
SSB 0
LB 0.30 Hz
GB 0
PC 1.00
9 8 7 6 5 4 3 2 1 ppm
1.00
1.01
1.00
1.03
6.10
2.00
0.98
0.97

4h

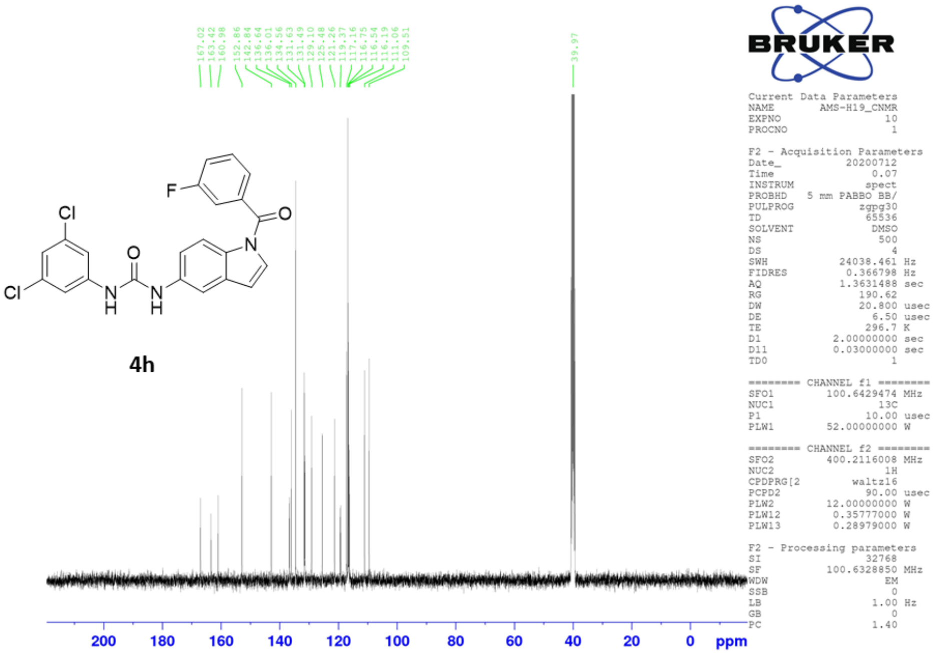
167.02
163.42
160.98
152.86
142.84
136.64
136.01
134.56
131.63
131.49
129.10
125.48
121.26
119.37
117.16
116.75
116.54
116.19
111.66
109.51
39.97
BRUKER
Current Data Parameters
NAME AMS-H19_CNMR
EXPNO 10
PROCNO 1
F2 - Acquisition Parameters
Date_ 20200712
Time 0.07
INSTRUM spect
PROBHD 5 mm PABBO BB/
PULPROG zgpg30
TD 65536
SOLVENT DMSO
NS 500
DS 4
SWH 24038.461 Hz
FIDRES 0.366798 Hz
AQ 1.3631488 sec
RG 190.62
DW 20.800 usec
DE 6.50 usec
TE 296.7 K
D1 2.00000000 sec
D11 0.03000000 sec
TD0 1
======== CHANNEL f1 ========
SFO1 100.6429474 MHz
NUC1 13C
P1 10.00 usec
PLW1 52.00000000 W
======== CHANNEL f2 ========
SFO2 400.2116008 MHz
NUC2 1H
CPDPRG[2 waltz16
PCPD2 90.00 usec
PLW2 12.00000000 W
PLW12 0.35777000 W
PLW13 0.28979000 W
F2 - Processing parameters
SI 32768
SF 100.6328850 MHz
WDW EM
SSB 0
LB 1.00 Hz
GB 0
PC 1.40
200 180 160 140 120 100 80 60 40 20 0 ppm

4h

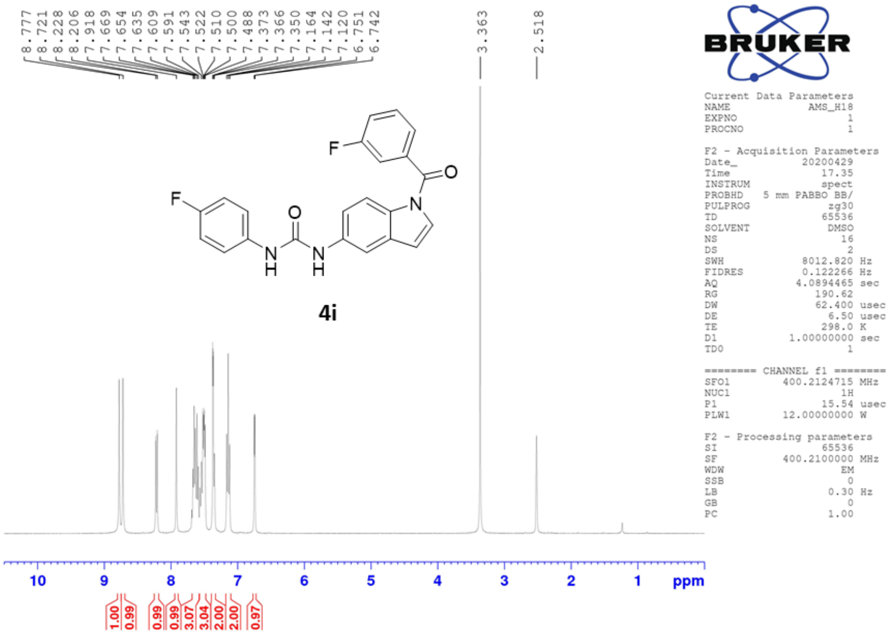

4i
BRUKER
Current Data Parameters
NAME AMS_H18
EXPNO 1
PROCNO 1
F2 - Acquisition Parameters
Date_ 20200429
Time 17.35
INSTRUM spect
PROBHD 5 mm PABBO BB/
PULPROG zg30
TD 65536
SOLVENT DMSO
NS 16
DS 2
SWH 8012.820 Hz
FIDRES 0.122266 Hz
AQ 4.0894465 sec
RG 190.62
DW 62.400 usec
DE 6.50 usec
TE 298.0 K
D1 1.00000000 sec
TD0 1
======== CHANNEL f1 ========
SFO1 400.2124715 MHz
NUC1 1H
P1 15.54 usec
PLW1 12.00000000 W
F2 - Processing parameters
SI 65536
SF 400.2100000 MHz
WDW EM
SSB 0
LB 0.30 Hz
GB 0
PC 1.00
ppm

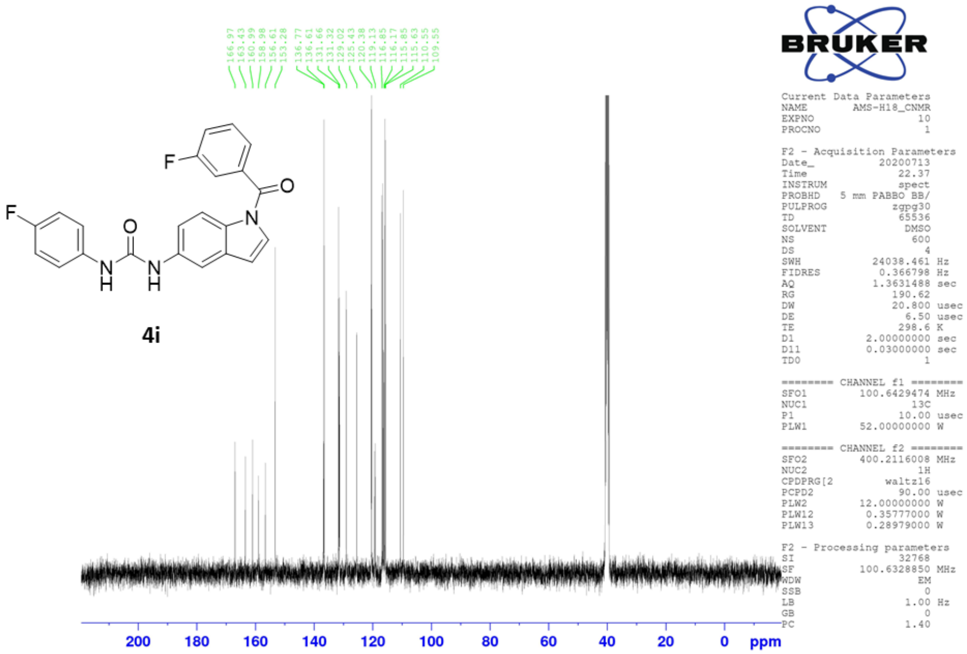

4i
BRUKER
Current Data Parameters
NAME AMS-H18_CNMR
EXPNO 10
PROCNO 1
F2 - Acquisition Parameters
Date_ 20200713
Time 22.37
INSTRUM spect
PROBHD 5 mm PABBO BB/
PULPROG zgpg30
TD 65536
SOLVENT DMSO
NS 600
DS 4
SWH 24038.461 Hz
FIDRES 0.366798 Hz
AQ 1.3631488 sec
RG 190.62
DW 20.800 usec
DE 6.50 usec
TE 298.6 K
D1 2.00000000 sec
D11 0.03000000 sec
TD0 1
======== CHANNEL f1 ========
SFO1 100.6429474 MHz
NUC1 13C
P1 10.00 usec
PLW1 52.00000000 W
======== CHANNEL f2 ========
SFO2 400.2116008 MHz
NUC2 1H
CPDPRG[2 waltz16
PCPD2 90.00 usec
PLW2 12.00000000 W
PLW12 0.35777000 W
PLW13 0.28979000 W
F2 - Processing parameters
SI 32768
SF 100.6328850 MHz
WDW EM
SSB 0
LB 1.00 Hz
GB 0
PC 1.40
ppm

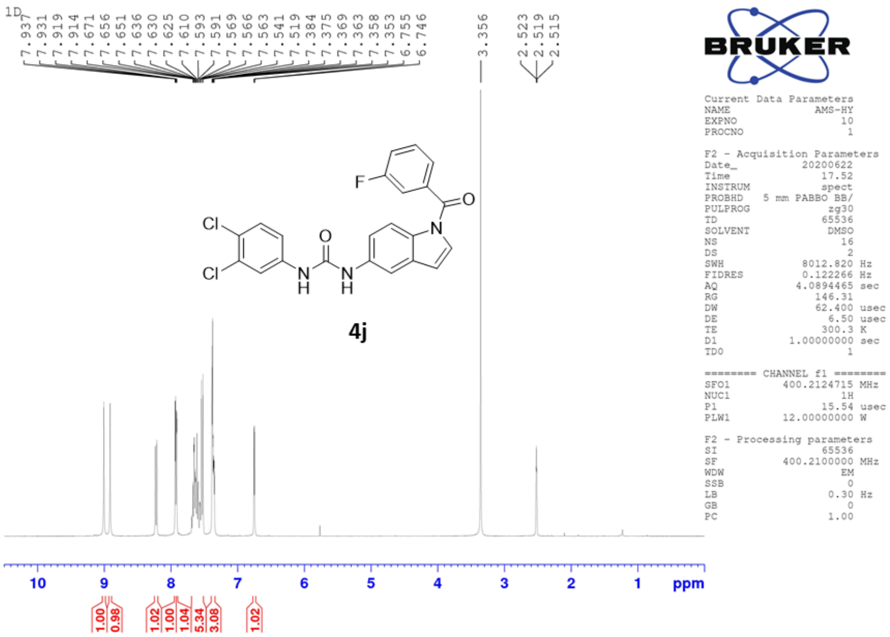
1D
7.937
7.931
7.919
7.914
7.671
7.656
7.651
7.636
7.630
7.625
7.610
7.593
7.591
7.569
7.566
7.563
7.541
7.519
7.384
7.375
7.369
7.363
7.358
7.353
6.755
6.746
3.356
2.523
2.519
2.515
F
O
Cl
Cl
O
N
N
H
N
H
4j
10 9 8 7 6 5 4 3 2 1 ppm
1.00
0.98
1.02
1.00
1.04
5.34
3.08
1.02
BRUKER
Current Data Parameters
NAME AMS-HY
EXPNO 10
PROCNO 1
F2 - Acquisition Parameters
Date_ 20200622
Time 17.52
INSTRUM spect
PROBHD 5 mm PABBO BB/
PULPROG zg30
TD 65536
SOLVENT DMSO
NS 16
DS 2
SWH 8012.820 Hz
FIDRES 0.122266 Hz
AQ 4.0894465 sec
RG 146.31
DW 62.400 usec
DE 6.50 usec
TE 300.3 K
D1 1.00000000 sec
TD0 1
======== CHANNEL f1 ========
SFO1 400.2124715 MHz
NUC1 1H
P1 15.54 usec
PLW1 12.00000000 W
F2 - Processing parameters
SI 65536
SF 400.2100000 MHz
WDW EM
SSB 0
LB 0.30 Hz
GB 0
PC 1.00

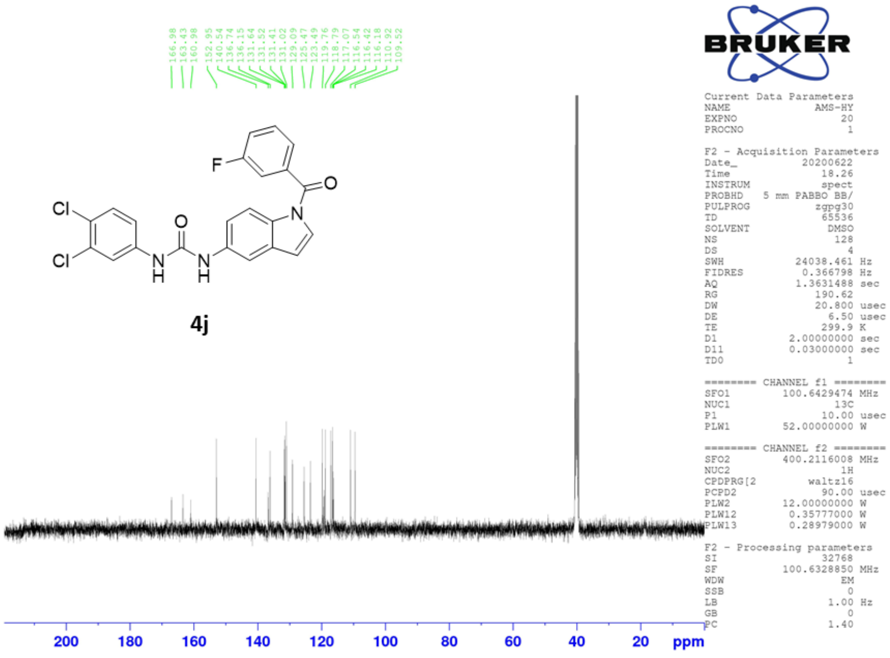
166.98
163.43
160.98
152.95
140.54
136.74
136.15
131.64
131.52
131.41
131.02
129.09
125.47
123.49
119.76
118.79
117.07
116.54
116.42
116.18
110.92
109.52
F
O
Cl
Cl
O
N
N
H
N
H
4j
200 180 160 140 120 100 80 60 40 20 ppm
BRUKER
Current Data Parameters
NAME AMS-HY
EXPNO 20
PROCNO 1
F2 - Acquisition Parameters
Date_ 20200622
Time 18.26
INSTRUM spect
PROBHD 5 mm PABBO BB/
PULPROG zgpg30
TD 65536
SOLVENT DMSO
NS 128
DS 4
SWH 24038.461 Hz
FIDRES 0.366798 Hz
AQ 1.3631488 sec
RG 190.62
DW 20.800 usec
DE 6.50 usec
TE 299.9 K
D1 2.00000000 sec
D11 0.03000000 sec
TD0 1
======== CHANNEL f1 ========
SFO1 100.6429474 MHz
NUC1 13C
P1 10.00 usec
PLW1 52.00000000 W
======== CHANNEL f2 ========
SFO2 400.2116008 MHz
NUC2 1H
CPDPRG[2 waltz16
PCPD2 90.00 usec
PLW2 12.00000000 W
PLW12 0.35777000 W
PLW13 0.28979000 W
F2 - Processing parameters
SI 32768
SF 100.6328850 MHz
WDW EM
SSB 0
LB 1.00 Hz
GB 0
PC 1.40

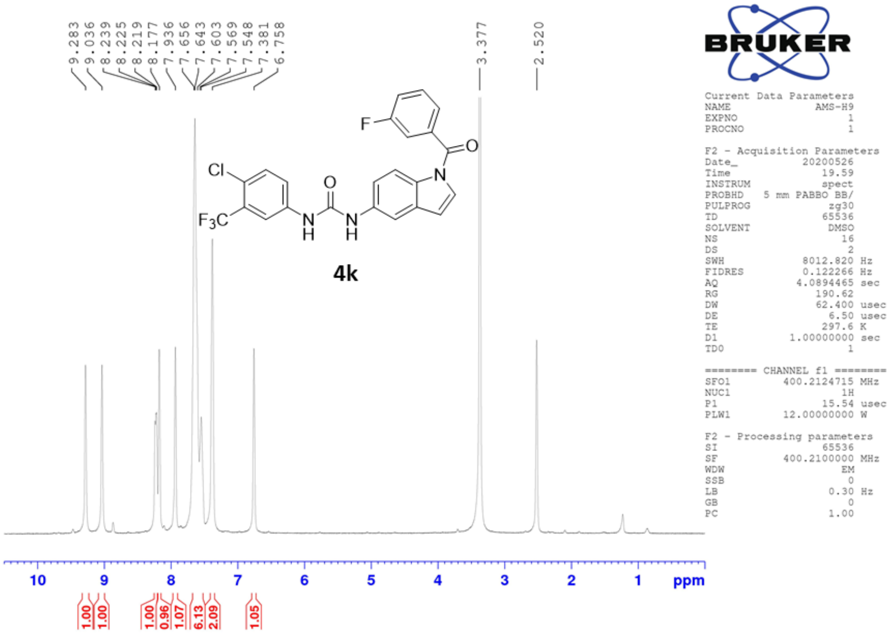
9.283
9.036
8.239
8.225
8.219
8.177
7.936
7.656
7.643
7.603
7.569
7.548
7.381
6.758
3.377
2.520
F
O
Cl
O
N
F3C
N H
N H
4k
BRUKER
Current Data Parameters
NAME AMS-H9
EXPNO 1
PROCNO 1
F2 - Acquisition Parameters
Date_ 20200526
Time 19.59
INSTRUM spect
PROBHD 5 mm PABBO BB/
PULPROG zg30
TD 65536
SOLVENT DMSO
NS 16
DS 2
SWH 8012.820 Hz
FIDRES 0.122266 Hz
AQ 4.0894465 sec
RG 190.62
DW 62.400 usec
DE 6.50 usec
TE 297.6 K
D1 1.00000000 sec
TD0 1
======== CHANNEL f1 ========
SFO1 400.2124715 MHz
NUC1 1H
P1 15.54 usec
PLW1 12.00000000 W
F2 - Processing parameters
SI 65536
SF 400.2100000 MHz
WDW EM
SSB 0
LB 0.30 Hz
GB 0
PC 1.00
10 9 8 7 6 5 4 3 2 1 ppm
1.00 1.00 1.00 0.96 1.07 6.13 2.09 1.05

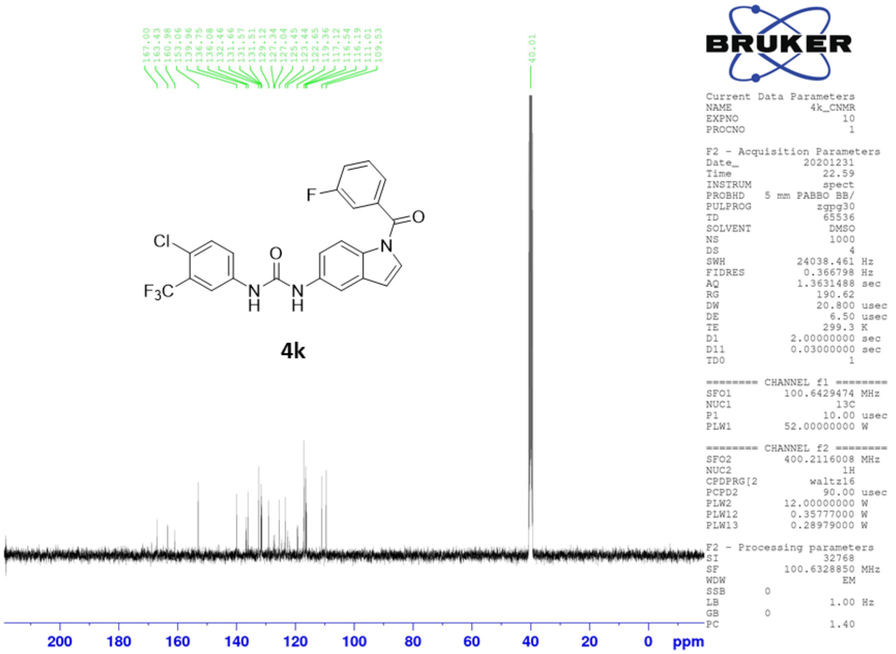
167.00
163.43
160.98
153.06
139.95
136.75
136.08
132.46
131.57
131.51
129.12
127.34
125.45
123.44
122.65
119.56
117.12
116.54
116.19
111.01
109.53
40.01
F
O
Cl
O
N
F3C
N H
N H
4k
BRUKER
Current Data Parameters
NAME 4k_CNMR
EXPNO 10
PROCNO 1
F2 - Acquisition Parameters
Date_ 20201231
Time 22.59
INSTRUM spect
PROBHD 5 mm PABBO BB/
PULPROG zgpg30
TD 65536
SOLVENT DMSO
NS 1000
DS 4
SWH 24038.461 Hz
FIDRES 0.366798 Hz
AQ 1.3631488 sec
RG 190.62
DW 20.800 usec
DE 6.50 usec
TE 299.3 K
D1 2.00000000 sec
D11 0.03000000 sec
TD0 1
======== CHANNEL f1 ========
SFO1 100.6429474 MHz
NUC1 13C
P1 10.00 usec
PLW1 52.00000000 W
======== CHANNEL f2 ========
SFO2 400.2116008 MHz
NUC2 1H
CPDPRG[2 waltz16
PCPD2 90.00 usec
PLW2 12.00000000 W
PLW12 0.35777000 W
PLW13 0.28979000 W
F2 - Processing parameters
SI 32768
SF 100.6328850 MHz
WDW EM
SSB 0
LB 1.00 Hz
GB 0
PC 1.40
200 180 160 140 120 100 80 60 40 20 0 ppm

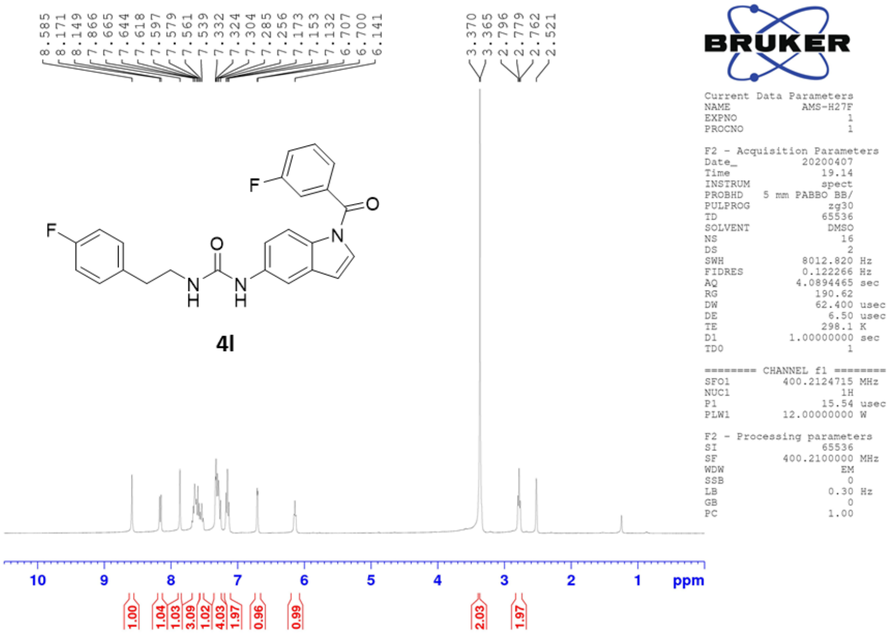
8.585 8.171 8.149 7.866 7.665 7.644 7.618 7.597 7.579 7.561 7.539 7.332 7.324 7.304 7.285 7.256 7.173 7.153 7.132 6.707 6.700 6.141
3.370 3.365 2.796 2.779 2.762 2.521
4l
1.00 1.04 1.03 3.09 1.02 4.03 1.97 0.96 0.99 2.03 1.97
10 9 8 7 6 5 4 3 2 1 ppm
BRUKER
Current Data Parameters
NAME AMS-H27F
EXPNO 1
PROCNO 1
F2 - Acquisition Parameters
Date_ 20200407
Time 19.14
INSTRUM spect
PROBHD 5 mm PABBO BB/
PULPROG zg30
TD 65536
SOLVENT DMSO
NS 16
DS 2
SWH 8012.820 Hz
FIDRES 0.122266 Hz
AQ 4.0894465 sec
RG 190.62
DW 62.400 usec
DE 6.50 usec
TE 298.1 K
D1 1.00000000 sec
TD0 1
======== CHANNEL f1 ========
SFO1 400.2124715 MHz
NUC1 1H
P1 15.54 usec
PLW1 12.00000000 W
F2 - Processing parameters
SI 65536
SF 400.2100000 MHz
WDW EM
SSB 0
LB 0.30 Hz
GB 0
PC 1.00

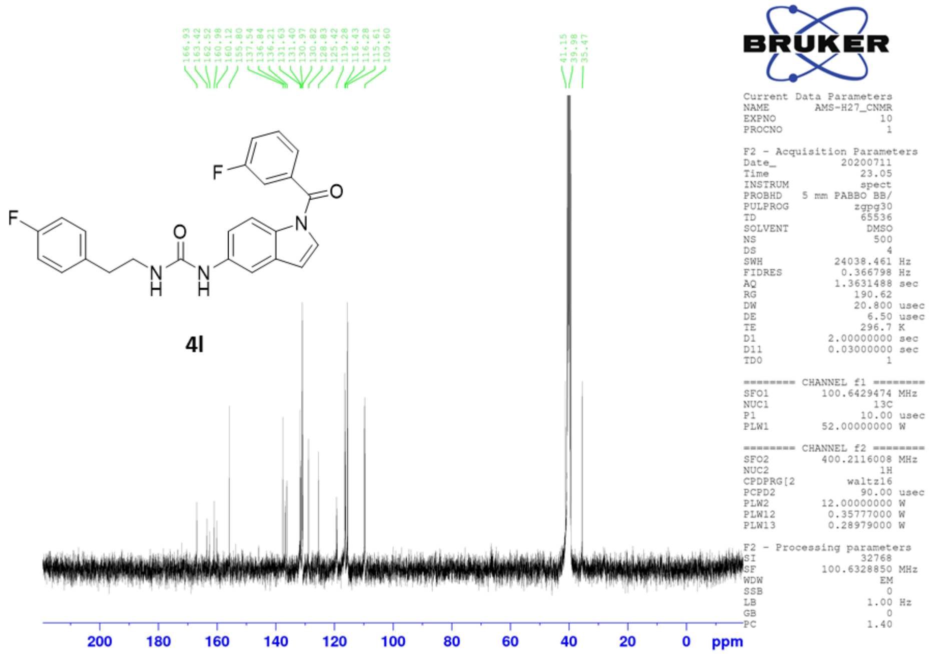
166.93 163.42 162.52 160.98 160.12 155.80 137.54 136.84 136.21 131.63 131.40 130.97 130.82 128.83 125.42 119.28 116.43 116.28 115.61 109.60
41.15 39.98 35.47
4l
200 180 160 140 120 100 80 60 40 20 0 ppm
BRUKER
Current Data Parameters
NAME AMS-H27_CNMR
EXPNO 10
PROCNO 1
F2 - Acquisition Parameters
Date_ 20200711
Time 23.05
INSTRUM spect
PROBHD 5 mm PABBO BB/
PULPROG zgpg30
TD 65536
SOLVENT DMSO
NS 500
DS 4
SWH 24038.461 Hz
FIDRES 0.366798 Hz
AQ 1.3631488 sec
RG 190.62
DW 20.800 usec
DE 6.50 usec
TE 296.7 K
D1 2.00000000 sec
D11 0.03000000 sec
TD0 1
======== CHANNEL f1 ========
SFO1 100.6429474 MHz
NUC1 13C
P1 10.00 usec
PLW1 52.00000000 W
======== CHANNEL f2 ========
SFO2 400.2116008 MHz
NUC2 1H
CPDPRG[2 waltz16
PCPD2 90.00 usec
PLW2 12.00000000 W
PLW12 0.35777000 W
PLW13 0.28979000 W
F2 - Processing parameters
SI 32768
SF 100.6328850 MHz
WDW EM
SSB 0
LB 1.00 Hz
GB 0
PC 1.40

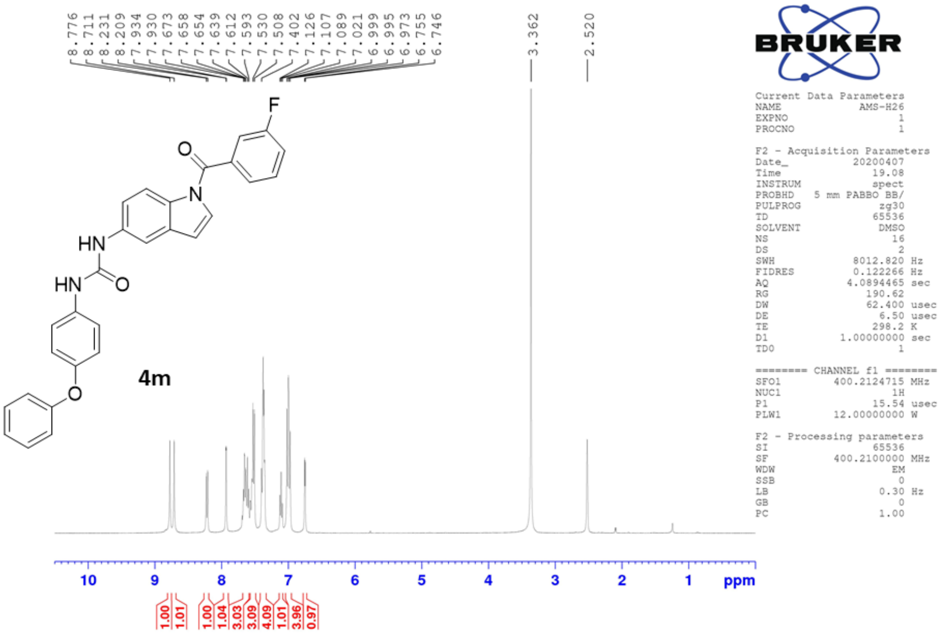
8.776
8.711
8.231
8.209
7.934
7.930
7.673
7.658
7.654
7.639
7.612
7.593
7.530
7.508
7.402
7.126
7.107
7.089
7.021
6.999
6.995
6.973
6.755
6.746
3.362
2.520
F
O
N
HN
HN
O
O
4m
1.00
1.01
1.00
1.04
3.03
3.09
4.09
1.01
3.96
0.97
10
9
8
7
6
5
4
3
2
1
ppm
BRUKER
Current Data Parameters
NAME AMS-H26
EXPNO 1
PROCNO 1
F2 - Acquisition Parameters
Date_ 20200407
Time 19.08
INSTRUM spect
PROBHD 5 mm PABBO BB/
PULPROG zg30
TD 65536
SOLVENT DMSO
NS 16
DS 2
SWH 8012.820 Hz
FIDRES 0.122266 Hz
AQ 4.0894465 sec
RG 190.62
DW 62.400 usec
DE 6.50 usec
TE 298.2 K
D1 1.00000000 sec
TD0 1
======== CHANNEL f1 ========
SFO1 400.2124715 MHz
NUC1 1H
P1 15.54 usec
PLW1 12.00000000 W
F2 - Processing parameters
SI 65536
SF 400.2100000 MHz
WDW EM
SSB 0
LB 0.30 Hz
GB 0
PC 1.00

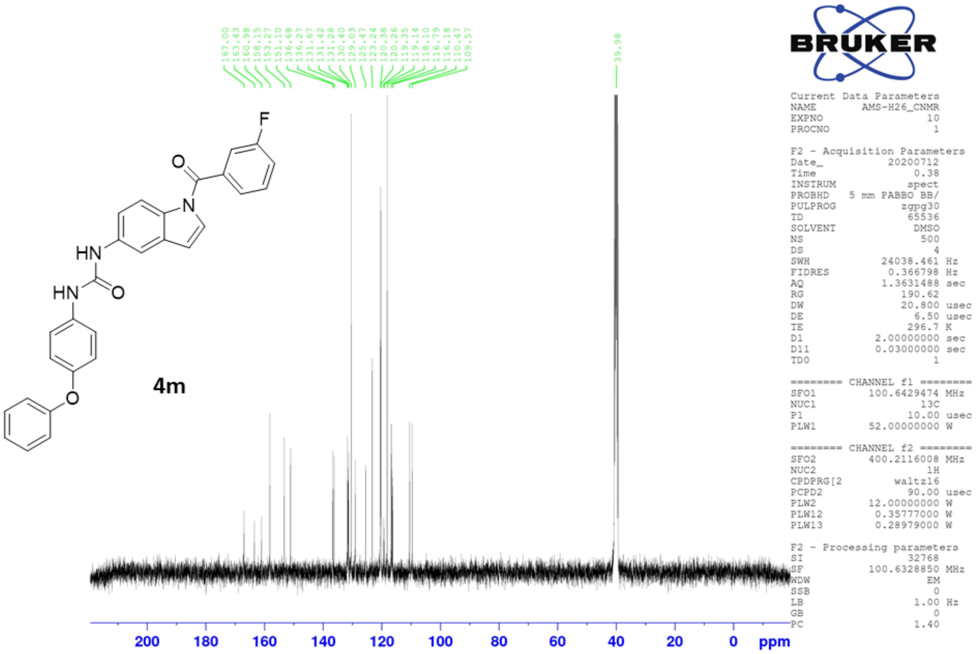
167.00
163.43
160.98
158.15
153.27
151.10
136.68
136.27
131.67
131.42
131.28
130.40
129.03
125.47
123.24
120.38
120.26
119.35
119.14
118.10
116.79
116.18
110.47
109.57
39.98
F
O
N
HN
HN
O
O
4m
200
180
160
140
120
100
80
60
40
20
0
ppm
BRUKER
Current Data Parameters
NAME AMS-H26_CNMR
EXPNO 10
PROCNO 1
F2 - Acquisition Parameters
Date_ 20200712
Time 0.38
INSTRUM spect
PROBHD 5 mm PABBO BB/
PULPROG zgpg30
TD 65536
SOLVENT DMSO
NS 500
DS 4
SWH 24038.461 Hz
FIDRES 0.366798 Hz
AQ 1.3631488 sec
RG 190.62
DW 20.800 usec
DE 6.50 usec
TE 296.7 K
D1 2.00000000 sec
D11 0.03000000 sec
TD0 1
======== CHANNEL f1 ========
SFO1 100.6429474 MHz
NUC1 13C
P1 10.00 usec
PLW1 52.00000000 W
======== CHANNEL f2 ========
SFO2 400.2116008 MHz
NUC2 1H
CPDPRG[2 waltz16
PCPD2 90.00 usec
PLW2 12.00000000 W
PLW12 0.35777000 W
PLW13 0.28979000 W
F2 - Processing parameters
SI 32768
SF 100.6328850 MHz
WDW EM
SSB 0
LB 1.00 Hz
GB 0
PC 1.40

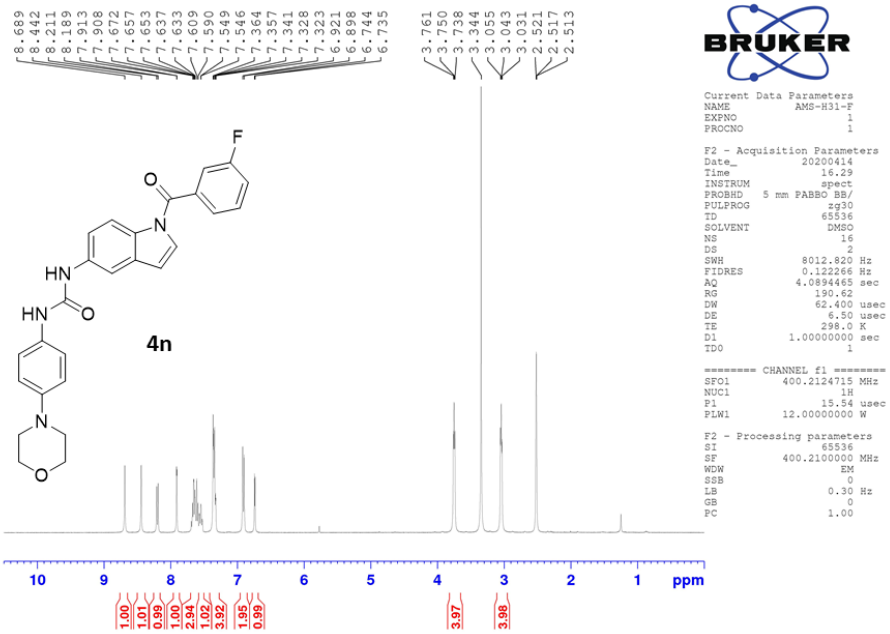
8.689 8.442 8.211 8.189 7.913 7.908 7.672 7.657 7.653 7.637 7.633 7.609 7.590 7.549 7.546 7.364 7.357 7.341 7.328 7.323 6.921 6.898 6.744 6.735
3.761 3.750 3.738 3.344 3.055 3.043 3.031 2.521 2.517 2.513
F
O
N
HN
HN
O
N
O
4n
10 9 8 7 6 5 4 3 2 1 ppm
1.00 1.01 0.99 1.00 2.94 1.02 3.92 1.95 0.99 3.97 3.98
BRUKER
Current Data Parameters
NAME AMS-H31-F
EXPNO 1
PROCNO 1
F2 - Acquisition Parameters
Date_ 20200414
Time 16.29
INSTRUM spect
PROBHD 5 mm PABBO BB/
PULPROG zg30
TD 65536
SOLVENT DMSO
NS 16
DS 2
SWH 8012.820 Hz
FIDRES 0.122266 Hz
AQ 4.0894465 sec
RG 190.62
DW 62.400 usec
DE 6.50 usec
TE 298.0 K
D1 1.00000000 sec
TD0 1
======== CHANNEL f1 ========
SFO1 400.2124715 MHz
NUC1 1H
P1 15.54 usec
PLW1 12.00000000 W
F2 - Processing parameters
SI 65536
SF 400.2100000 MHz
WDW EM
SSB 0
LB 0.30 Hz
GB 0
PC 1.00

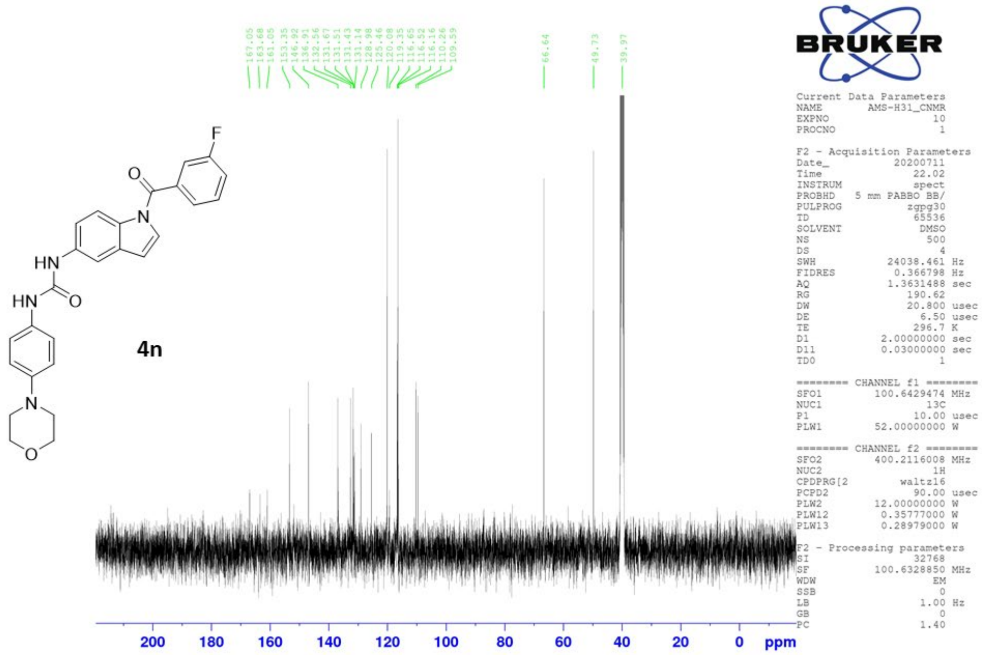
167.05 163.68 161.05 153.35 146.92 136.91 132.56 131.67 131.51 131.43 131.14 128.98 125.46 120.08 119.35 116.65 116.52 110.16 110.08 109.59
66.64 49.73 39.97
F
O
N
HN
HN
O
N
O
4n
200 180 160 140 120 100 80 60 40 20 0 ppm
BRUKER
Current Data Parameters
NAME AMS-H31_CNMR
EXPNO 10
PROCNO 1
F2 - Acquisition Parameters
Date_ 20200711
Time 22.02
INSTRUM spect
PROBHD 5 mm PABBO BB/
PULPROG zgpg30
TD 65536
SOLVENT DMSO
NS 500
DS 4
SWH 24038.461 Hz
FIDRES 0.366798 Hz
AQ 1.3631488 sec
RG 190.62
DW 20.800 usec
DE 6.50 usec
TE 296.7 K
D1 2.00000000 sec
D11 0.03000000 sec
TD0 1
======== CHANNEL f1 ========
SFO1 100.6429474 MHz
NUC1 13C
P1 10.00 usec
PLW1 52.00000000 W
======== CHANNEL f2 ========
SFO2 400.2116008 MHz
NUC2 1H
CPDPRG[2 waltz16
PCPD2 90.00 usec
PLW2 12.00000000 W
PLW12 0.35777000 W
PLW13 0.28979000 W
F2 - Processing parameters
SI 32768
SF 100.6328850 MHz
WDW EM
SSB 0
LB 1.00 Hz
GB 0
PC 1.40

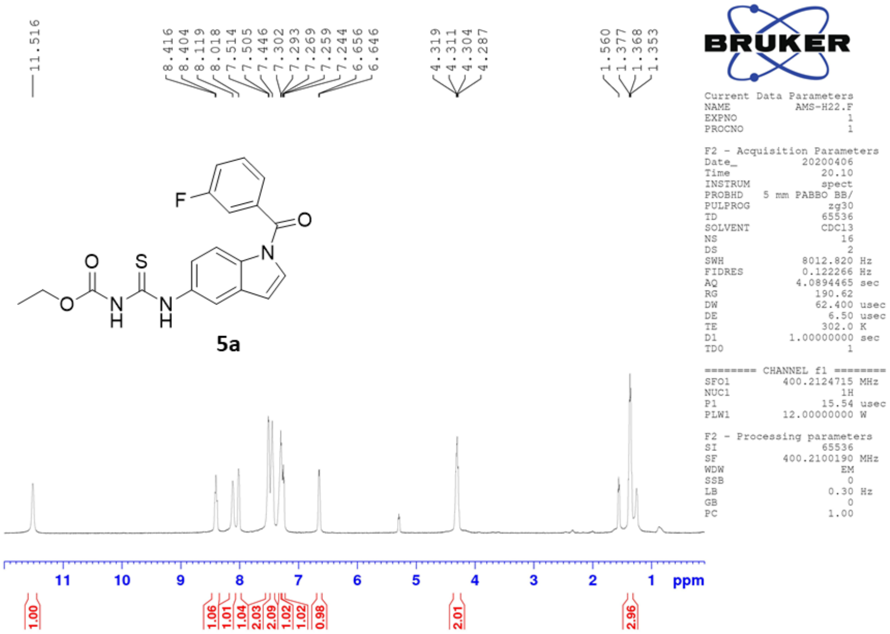
5a
Current Data Parameters
NAME AMS-H22.F
EXPNO 1
PROCNO 1
F2 - Acquisition Parameters
Date_ 20200406
INSTRUM spect
PULPROG zg30
SOLVENT CDCl3
NS 16
SFO1 400.2124715 MHz
NUC1 1H
ppm

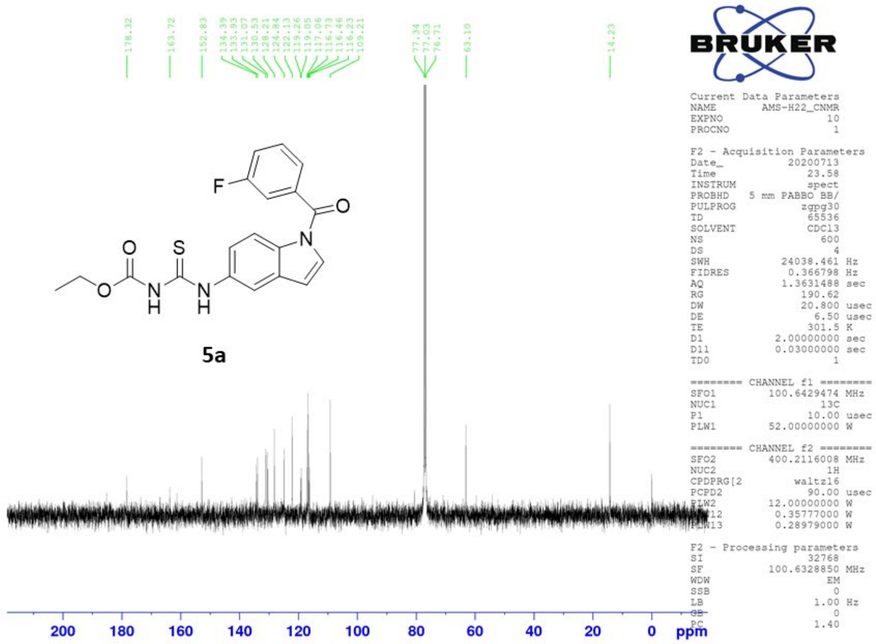
5a
Current Data Parameters
NAME AMS-H22_CNMR
EXPNO 10
PROCNO 1
F2 - Acquisition Parameters
Date_ 20200713
INSTRUM spect
PULPROG zgpg30
SOLVENT CDCl3
NS 600
SFO1 100.6429474 MHz
NUC1 13C
ppm

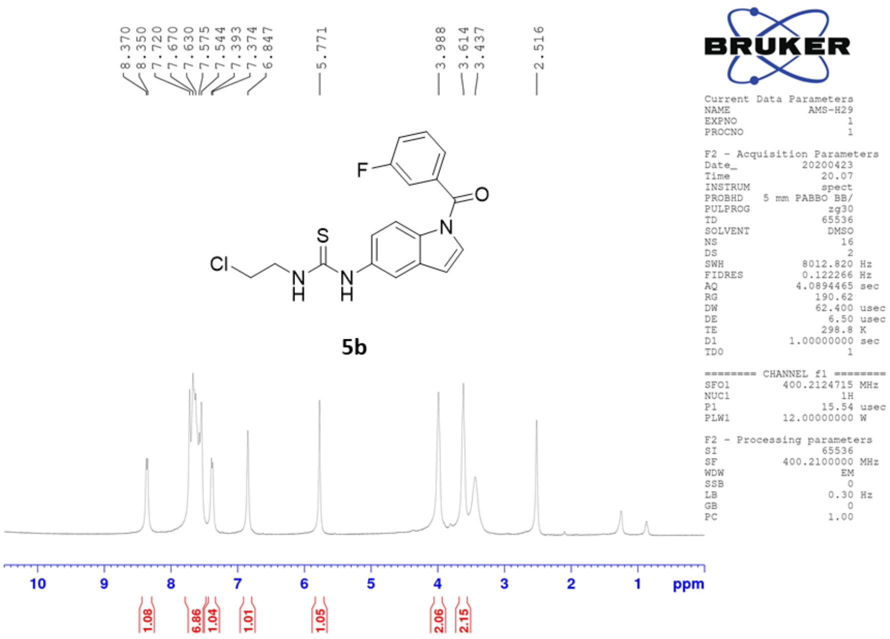
8.370
8.350
7.720
7.670
7.630
7.575
7.544
7.393
7.374
6.847
5.771
3.988
3.614
3.437
2.516
F
O
N
S
Cl
N
H
N
H
5b
10
9
8
7
6
5
4
3
2
1
ppm
1.08
6.86
1.04
1.01
1.05
2.06
2.15
BRUKER
Current Data Parameters
NAME AMS-H29
EXPNO 1
PROCNO 1
F2 - Acquisition Parameters
Date_ 20200423
Time 20.07
INSTRUM spect
PROBHD 5 mm PABBO BB/
PULPROG zg30
TD 65536
SOLVENT DMSO
NS 16
DS 2
SWH 8012.820 Hz
FIDRES 0.122266 Hz
AQ 4.0894465 sec
RG 190.62
DW 62.400 usec
DE 6.50 usec
TE 298.8 K
D1 1.00000000 sec
TD0 1
======== CHANNEL f1 ========
SFO1 400.2124715 MHz
NUC1 1H
P1 15.54 usec
PLW1 12.00000000 W
F2 - Processing parameters
SI 65536
SF 400.2100000 MHz
WDW EM
SSB 0
LB 0.30 Hz
GB 0
PC 1.00

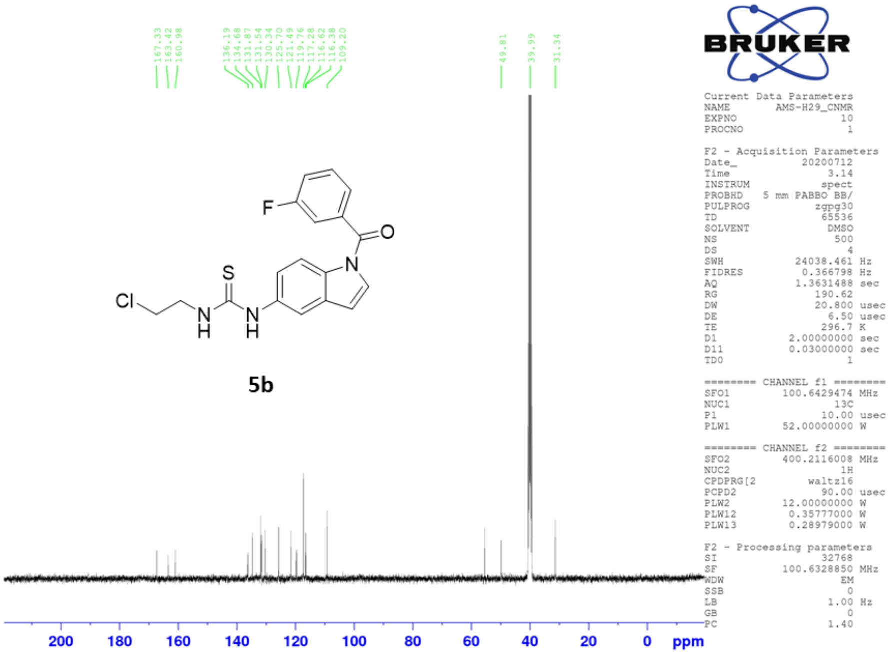
167.33
163.42
160.98
136.19
134.68
131.87
131.54
130.34
125.70
121.49
119.76
117.62
116.38
109.20
49.81
39.99
31.34
F
O
N
S
Cl
N
H
N
H
5b
200
180
160
140
120
100
80
60
40
20
0
ppm
BRUKER
Current Data Parameters
NAME AMS-H29_CNMR
EXPNO 10
PROCNO 1
F2 - Acquisition Parameters
Date_ 20200712
Time 3.14
INSTRUM spect
PROBHD 5 mm PABBO BB/
PULPROG zgpg30
TD 65536
SOLVENT DMSO
NS 500
DS 4
SWH 24038.461 Hz
FIDRES 0.366798 Hz
AQ 1.3631488 sec
RG 190.62
DW 20.800 usec
DE 6.50 usec
TE 296.7 K
D1 2.00000000 sec
D11 0.03000000 sec
TD0 1
======== CHANNEL f1 ========
SFO1 100.6429474 MHz
NUC1 13C
P1 10.00 usec
PLW1 52.00000000 W
======== CHANNEL f2 ========
SFO2 400.2116008 MHz
NUC2 1H
CPDPRG[2 waltz16
PCPD2 90.00 usec
PLW2 12.00000000 W
PLW12 0.35777000 W
PLW13 0.28979000 W
F2 - Processing parameters
SI 32768
SF 100.6328850 MHz
WDW EM
SSB 0
LB 1.00 Hz
GB 0
PC 1.40

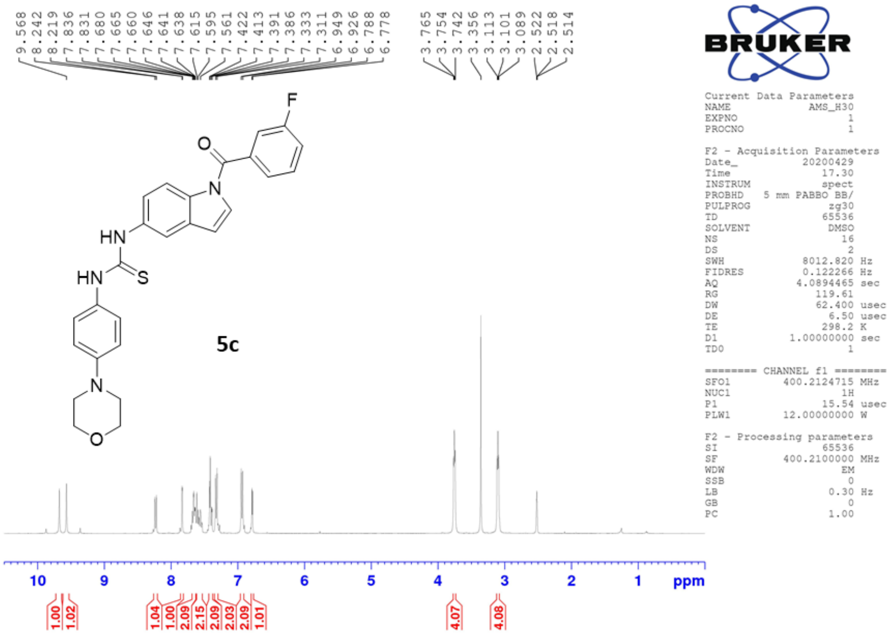
9.568
8.242
8.219
7.836
7.831
7.680
7.665
7.660
7.646
7.641
7.638
7.615
7.595
7.561
7.422
7.413
7.391
7.386
7.333
7.311
6.949
6.926
6.788
6.778
3.765
3.754
3.742
3.356
3.113
3.101
3.089
2.522
2.518
2.514
F
O
N
HN
HN
S
N
O
5c
10 9 8 7 6 5 4 3 2 1 ppm
1.00 1.02 1.04 1.00 2.09 2.15 2.09 2.03 2.09 1.01 4.07 4.08
BRUKER
Current Data Parameters
NAME AMS_H30
EXPNO 1
PROCNO 1
F2 - Acquisition Parameters
Date_ 20200429
Time 17.30
INSTRUM spect
PROBHD 5 mm PABBO BB/
PULPROG zg30
TD 65536
SOLVENT DMSO
NS 16
DS 2
SWH 8012.820 Hz
FIDRES 0.122266 Hz
AQ 4.0894465 sec
RG 119.61
DW 62.400 usec
DE 6.50 usec
TE 298.2 K
D1 1.00000000 sec
TD0 1
======== CHANNEL f1 ========
SFO1 400.2124715 MHz
NUC1 1H
P1 15.54 usec
PLW1 12.00000000 W
F2 - Processing parameters
SI 65536
SF 400.2100000 MHz
WDW EM
SSB 0
LB 0.30 Hz
GB 0
PC 1.00

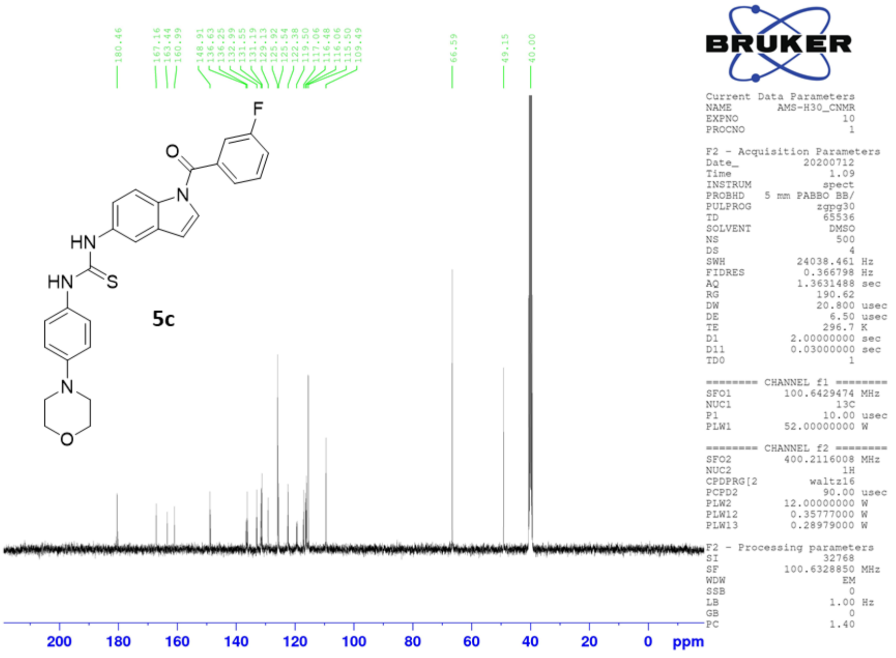
180.46
167.16
163.44
160.99
148.91
136.63
136.25
132.99
131.55
131.19
129.13
125.92
125.54
122.38
119.50
117.06
116.48
116.06
115.50
109.49
66.59
49.15
40.00
F
O
N
HN
HN
S
N
O
5c
200 180 160 140 120 100 80 60 40 20 0 ppm
BRUKER
Current Data Parameters
NAME AMS-H30_CNMR
EXPNO 10
PROCNO 1
F2 - Acquisition Parameters
Date_ 20200712
Time 1.09
INSTRUM spect
PROBHD 5 mm PABBO BB/
PULPROG zgpg30
TD 65536
SOLVENT DMSO
NS 500
DS 4
SWH 24038.461 Hz
FIDRES 0.366798 Hz
AQ 1.3631488 sec
RG 190.62
DW 20.800 usec
DE 6.50 usec
TE 296.7 K
D1 2.00000000 sec
D11 0.03000000 sec
TD0 1
======== CHANNEL f1 ========
SFO1 100.6429474 MHz
NUC1 13C
P1 10.00 usec
PLW1 52.00000000 W
======== CHANNEL f2 ========
SFO2 400.2116008 MHz
NUC2 1H
CPDPRG[2 waltz16
PCPD2 90.00 usec
PLW2 12.00000000 W
PLW12 0.35777000 W
PLW13 0.28979000 W
F2 - Processing parameters
SI 32768
SF 100.6328850 MHz
WDW EM
SSB 0
LB 1.00 Hz
GB 0
PC 1.40

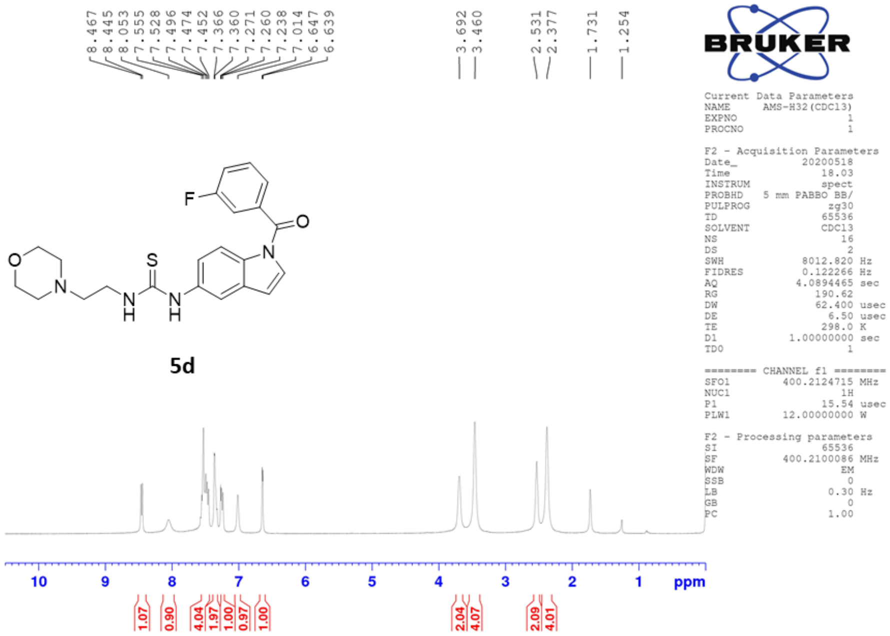
8.467
8.445
8.053
7.555
7.528
7.496
7.474
7.452
7.366
7.360
7.271
7.260
7.238
7.014
6.647
6.639
3.692
3.460
2.531
2.377
1.731
1.254
F
O
S
O
N
N
H
N
H
N
5d
10 9 8 7 6 5 4 3 2 1 ppm
1.07
0.90
4.04
1.97
1.00
0.97
1.00
2.04
4.07
2.09
4.01
BRUKER
Current Data Parameters
NAME AMS-H32(CDCl3)
EXPNO 1
PROCNO 1
F2 - Acquisition Parameters
Date_ 20200518
Time 18.03
INSTRUM spect
PROBHD 5 mm PABBO BB/
PULPROG zg30
TD 65536
SOLVENT CDCl3
NS 16
DS 2
SWH 8012.820 Hz
FIDRES 0.122266 Hz
AQ 4.0894465 sec
RG 190.62
DW 62.400 usec
DE 6.50 usec
TE 298.0 K
D1 1.00000000 sec
TD0 1
======== CHANNEL f1 ========
SFO1 400.2124715 MHz
NUC1 1H
P1 15.54 usec
PLW1 12.00000000 W
F2 - Processing parameters
SI 65536
SF 400.2100086 MHz
WDW EM
SSB 0
LB 0.30 Hz
GB 0
PC 1.00

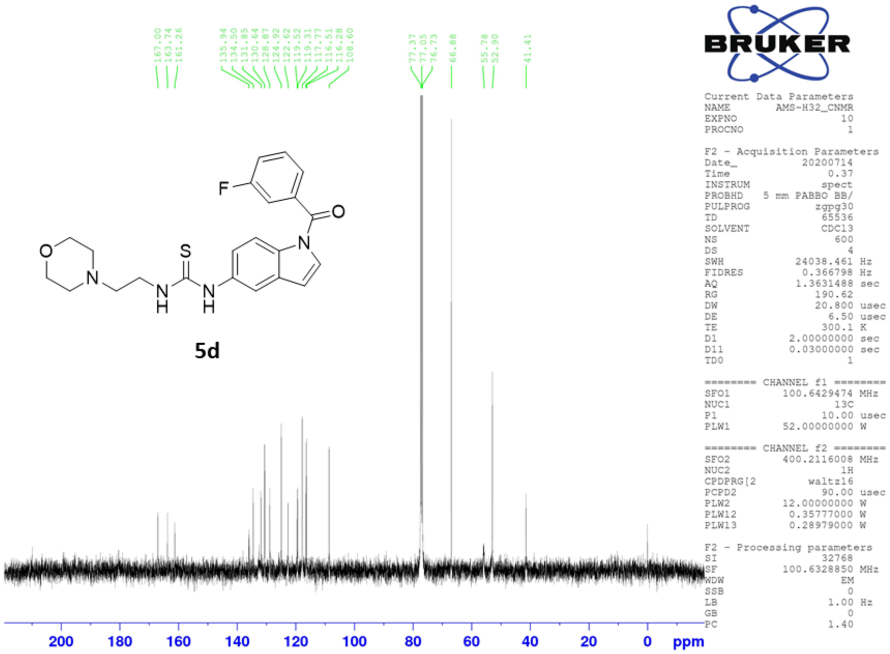
167.00
163.74
161.26
135.94
134.50
131.65
130.64
128.87
124.92
122.62
119.52
119.31
117.77
116.51
116.28
108.60
77.37
77.05
76.73
66.88
55.78
52.90
41.41
F
O
S
O
N
N
H
N
H
N
5d
200 180 160 140 120 100 80 60 40 20 0 ppm
BRUKER
Current Data Parameters
NAME AMS-H32_CNMR
EXPNO 10
PROCNO 1
F2 - Acquisition Parameters
Date_ 20200714
Time 0.37
INSTRUM spect
PROBHD 5 mm PABBO BB/
PULPROG zgpg30
TD 65536
SOLVENT CDCl3
NS 600
DS 4
SWH 24038.461 Hz
FIDRES 0.366798 Hz
AQ 1.3631488 sec
RG 190.62
DW 20.800 usec
DE 6.50 usec
TE 300.1 K
D1 2.00000000 sec
D11 0.03000000 sec
TD0 1
======== CHANNEL f1 ========
SFO1 100.6429474 MHz
NUC1 13C
P1 10.00 usec
PLW1 52.00000000 W
======== CHANNEL f2 ========
SFO2 400.2116008 MHz
NUC2 1H
CPDPRG[2 waltz16
PCPD2 90.00 usec
PLW2 12.00000000 W
PLW12 0.35777000 W
PLW13 0.28979000 W
F2 - Processing parameters
SI 32768
SF 100.6328850 MHz
WDW EM
SSB 0
LB 1.00 Hz
GB 0
PC 1.40

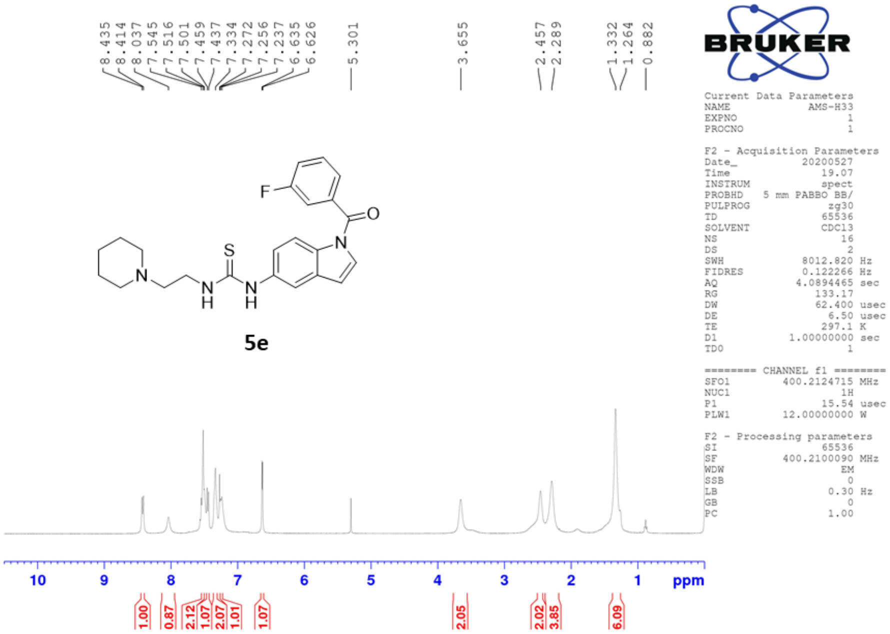
8.435
8.414
8.037
7.545
7.516
7.501
7.459
7.437
7.334
7.272
7.256
7.237
6.635
6.626
5.301
3.655
2.457
2.289
1.332
1.264
0.882
BRUKER
5e
10 9 8 7 6 5 4 3 2 1 ppm
1.00
0.87
2.12
1.07
2.07
1.01
1.07
2.05
2.02
3.85
6.09

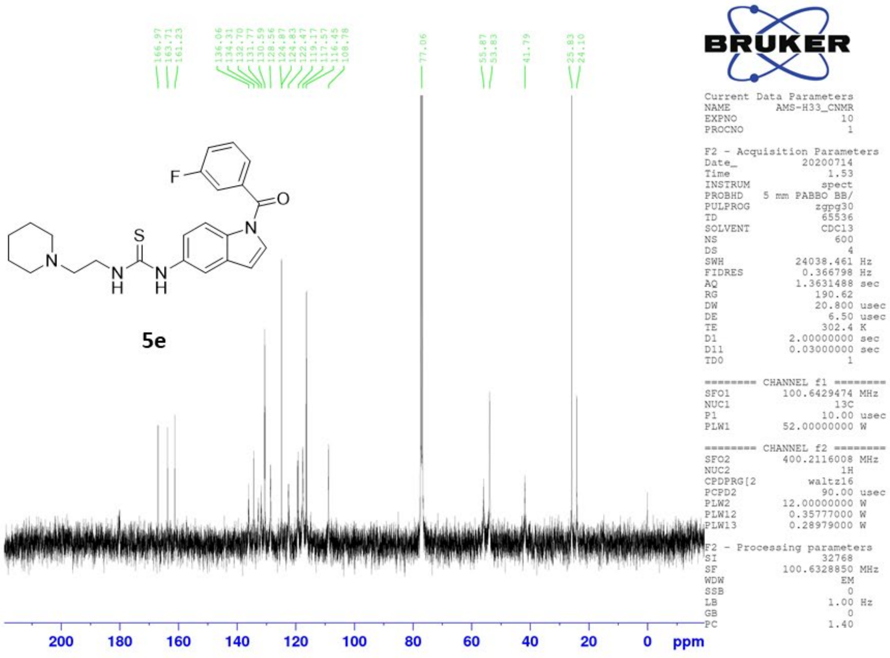
166.97
163.71
161.23
136.06
134.31
132.70
131.77
130.59
128.56
124.87
124.43
122.47
119.17
117.52
116.45
108.78
77.06
55.87
53.83
41.79
25.83
24.10
BRUKER
5e
200 180 160 140 120 100 80 60 40 20 0 ppm

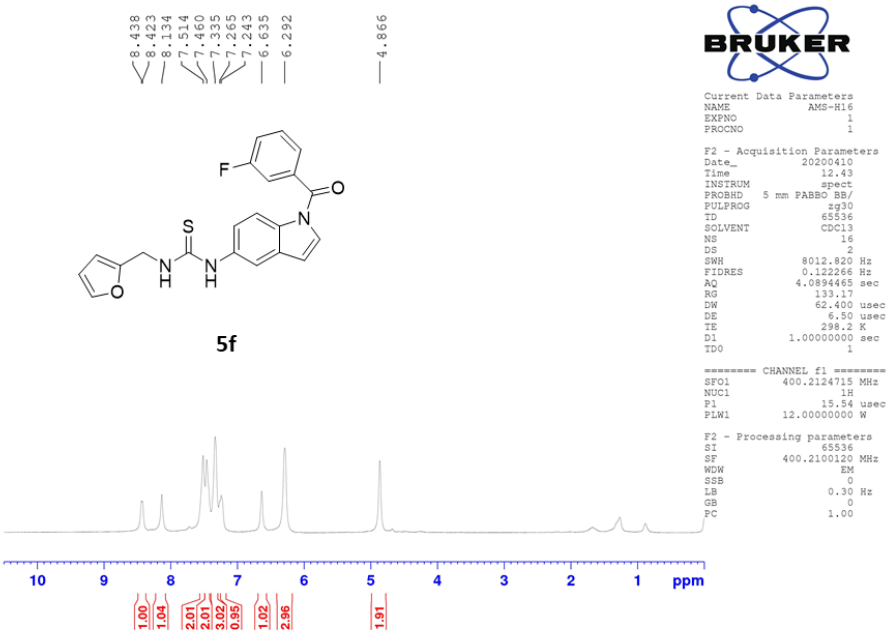
8.438
8.423
8.134
7.514
7.460
7.335
7.265
7.243
6.635
6.292
4.866
5f
10 9 8 7 6 5 4 3 2 1 ppm
1.00
1.04
2.01
2.01
3.02
0.95
1.02
2.96
1.91
BRUKER
Current Data Parameters
NAME AMS-H16
EXPNO 1
PROCNO 1
F2 - Acquisition Parameters
Date_ 20200410
Time 12.43
INSTRUM spect
PROBHD 5 mm PABBO BB/
PULPROG zg30
TD 65536
SOLVENT CDCl3
NS 16
DS 2
SWH 8012.820 Hz
FIDRES 0.122266 Hz
AQ 4.0894465 sec
RG 133.17
DW 62.400 usec
DE 6.50 usec
TE 298.2 K
D1 1.00000000 sec
TD0 1
======== CHANNEL f1 ========
SFO1 400.2124715 MHz
NUC1 1H
P1 15.54 usec
PLW1 12.00000000 W
F2 - Processing parameters
SI 65536
SF 400.2100120 MHz
WDW EM
SSB 0
LB 0.30 Hz
GB 0
PC 1.00

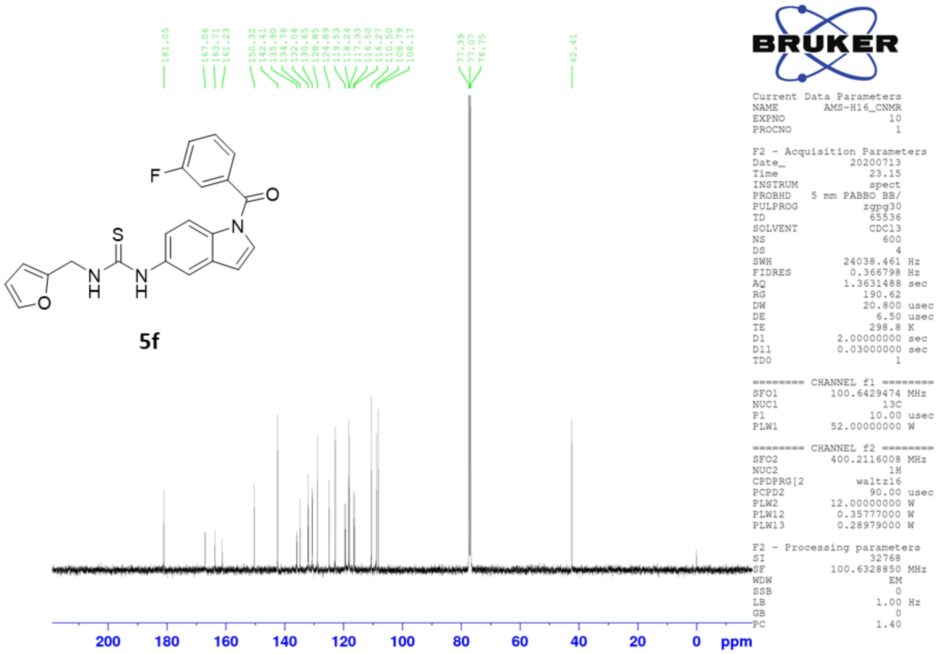
181.05
167.06
163.71
161.23
150.32
142.41
135.90
134.76
132.04
130.65
128.85
124.89
119.53
118.24
117.93
116.50
116.27
110.50
108.79
108.17
77.39
77.07
76.75
42.41
5f
200 180 160 140 120 100 80 60 40 20 0 ppm
BRUKER
Current Data Parameters
NAME AMS-H16_CNMR
EXPNO 10
PROCNO 1
F2 - Acquisition Parameters
Date_ 20200713
Time 23.15
INSTRUM spect
PROBHD 5 mm PABBO BB/
PULPROG zgpg30
TD 65536
SOLVENT CDCl3
NS 600
DS 4
SWH 24038.461 Hz
FIDRES 0.366798 Hz
AQ 1.3631488 sec
RG 190.62
DW 20.800 usec
DE 6.50 usec
TE 298.8 K
D1 2.00000000 sec
D11 0.03000000 sec
TD0 1
======== CHANNEL f1 ========
SFO1 100.6429474 MHz
NUC1 13C
P1 10.00 usec
PLW1 52.00000000 W
======== CHANNEL f2 ========
SFO2 400.2116008 MHz
NUC2 1H
CPDPRG[2 waltz16
PCPD2 90.00 usec
PLW2 12.00000000 W
PLW12 0.35777000 W
PLW13 0.28979000 W
F2 - Processing parameters
SI 32768
SF 100.6328850 MHz
WDW EM
SSB 0
LB 1.00 Hz
GB 0
PC 1.40

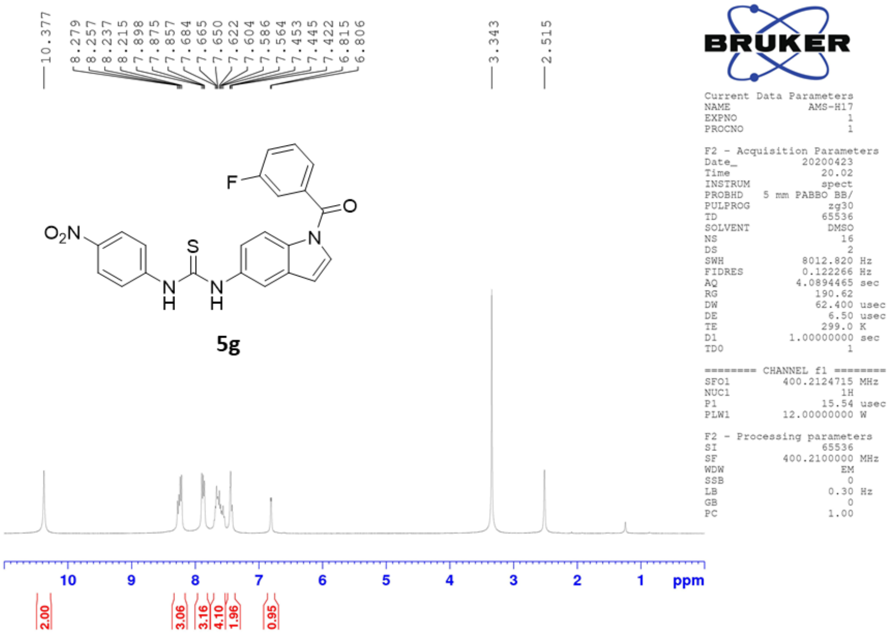
10.377
8.279
8.257
8.237
8.215
7.898
7.875
7.857
7.684
7.665
7.650
7.622
7.604
7.586
7.564
7.453
7.445
7.422
6.815
6.806
3.343
2.515
F
O
O_2N
S
N
H
5g
BRUKER
Current Data Parameters
NAME AMS-H17
EXPNO 1
PROCNO 1
F2 - Acquisition Parameters
Date_ 20200423
Time 20.02
INSTRUM spect
PROBHD 5 mm PABBO BB/
PULPROG zg30
TD 65536
SOLVENT DMSO
NS 16
DS 2
SWH 8012.820 Hz
FIDRES 0.122266 Hz
AQ 4.0894465 sec
RG 190.62
DW 62.400 usec
DE 6.50 usec
TE 299.0 K
D1 1.00000000 sec
TD0 1
======== CHANNEL f1 ========
SFO1 400.2124715 MHz
NUC1 1H
P1 15.54 usec
PLW1 12.00000000 W
F2 - Processing parameters
SI 65536
SF 400.2100000 MHz
WDW EM
SSB 0
LB 0.30 Hz
GB 0
PC 1.00
10 9 8 7 6 5 4 3 2 1 ppm
2.00
3.06
3.16
4.10
1.96
0.95

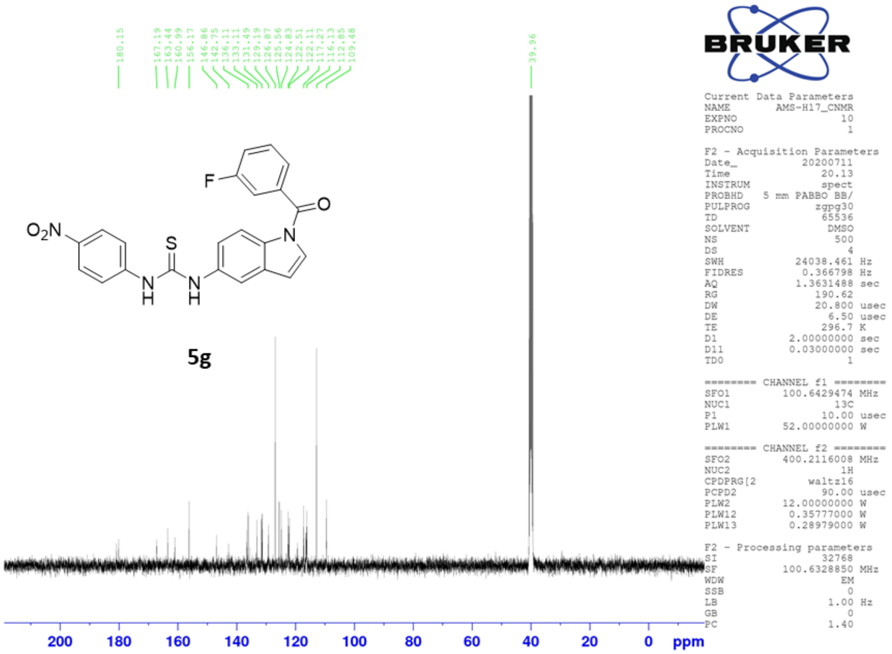
180.15
167.19
163.44
160.99
156.17
148.86
142.75
136.11
133.11
131.49
129.19
126.87
125.56
124.83
122.51
122.11
117.27
116.13
112.85
109.48
39.96
F
O
O_2N
S
N
H
5g
BRUKER
Current Data Parameters
NAME AMS-H17_CNMR
EXPNO 10
PROCNO 1
F2 - Acquisition Parameters
Date_ 20200711
Time 20.13
INSTRUM spect
PROBHD 5 mm PABBO BB/
PULPROG zgpg30
TD 65536
SOLVENT DMSO
NS 500
DS 4
SWH 24038.461 Hz
FIDRES 0.366798 Hz
AQ 1.3631488 sec
RG 190.62
DW 20.800 usec
DE 6.50 usec
TE 296.7 K
D1 2.00000000 sec
D11 0.03000000 sec
TD0 1
======== CHANNEL f1 ========
SFO1 100.6429474 MHz
NUC1 13C
P1 10.00 usec
PLW1 52.00000000 W
======== CHANNEL f2 ========
SFO2 400.2116008 MHz
NUC2 1H
CPDPRG[2 waltz16
PCPD2 90.00 usec
PLW2 12.00000000 W
PLW12 0.35777000 W
PLW13 0.28979000 W
F2 - Processing parameters
SI 32768
SF 100.6328850 MHz
WDW EM
SSB 0
LB 1.00 Hz
GB 0
PC 1.40
200 180 160 140 120 100 80 60 40 20 0 ppm

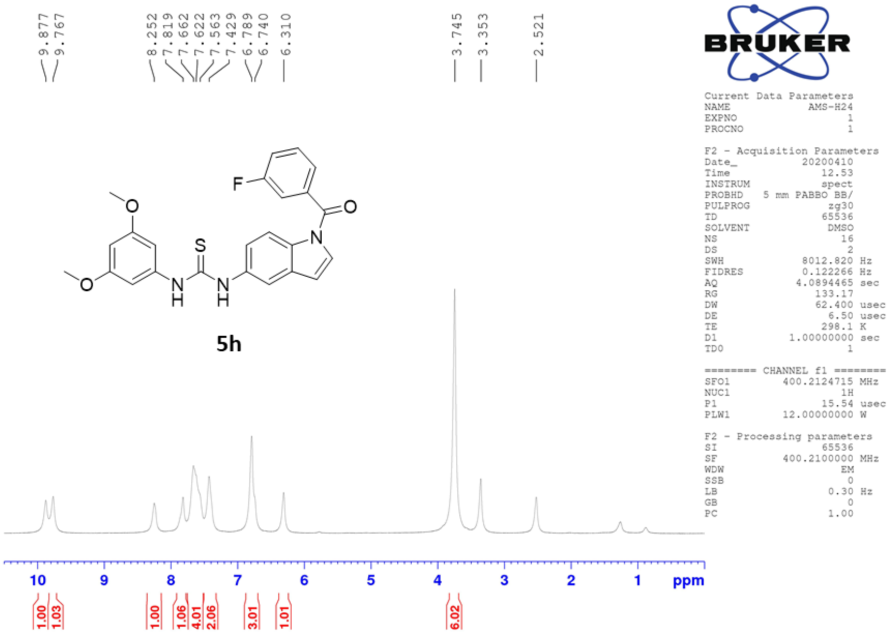
9.877
9.767
8.252
7.819
7.662
7.622
7.563
7.429
6.789
6.740
6.310
3.745
3.353
2.521
F
O
O
O
S
N
N
H
N
H
5h
10 9 8 7 6 5 4 3 2 1 ppm
1.00 1.03 1.00 1.06 4.01 2.06 3.01 1.01 6.02
BRUKER
Current Data Parameters
NAME AMS-H24
EXPNO 1
PROCNO 1
F2 - Acquisition Parameters
Date_ 20200410
Time 12.53
INSTRUM spect
PROBHD 5 mm PABBO BB/
PULPROG zg30
TD 65536
SOLVENT DMSO
NS 16
DS 2
SWH 8012.820 Hz
FIDRES 0.122266 Hz
AQ 4.0894465 sec
RG 133.17
DW 62.400 usec
DE 6.50 usec
TE 298.1 K
D1 1.00000000 sec
TD0 1
======== CHANNEL f1 ========
SFO1 400.2124715 MHz
NUC1 1H
P1 15.54 usec
PLW1 12.00000000 W
F2 - Processing parameters
SI 65536
SF 400.2100000 MHz
WDW EM
SSB 0
LB 0.30 Hz
GB 0
PC 1.00

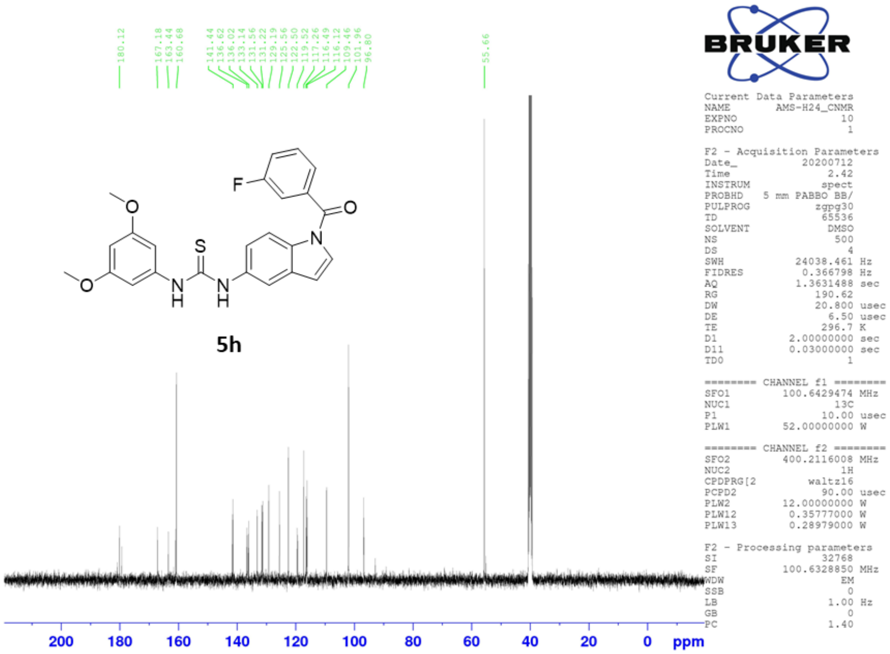
180.12
167.18
163.44
160.68
141.44
136.62
136.02
133.14
131.56
131.22
129.19
125.56
122.50
119.52
117.26
116.49
116.12
109.46
101.96
96.80
55.66
F
O
O
O
S
N
N
H
N
H
5h
200 180 160 140 120 100 80 60 40 20 0 ppm
BRUKER
Current Data Parameters
NAME AMS-H24_CNMR
EXPNO 10
PROCNO 1
F2 - Acquisition Parameters
Date_ 20200712
Time 2.42
INSTRUM spect
PROBHD 5 mm PABBO BB/
PULPROG zgpg30
TD 65536
SOLVENT DMSO
NS 500
DS 4
SWH 24038.461 Hz
FIDRES 0.366798 Hz
AQ 1.3631488 sec
RG 190.62
DW 20.800 usec
DE 6.50 usec
TE 296.7 K
D1 2.00000000 sec
D11 0.03000000 sec
TD0 1
======== CHANNEL f1 ========
SFO1 100.6429474 MHz
NUC1 13C
P1 10.00 usec
PLW1 52.00000000 W
======== CHANNEL f2 ========
SFO2 400.2116008 MHz
NUC2 1H
CPDPRG[2 waltz16
PCPD2 90.00 usec
PLW2 12.00000000 W
PLW12 0.35777000 W
PLW13 0.28979000 W
F2 - Processing parameters
SI 32768
SF 100.6328850 MHz
WDW EM
SSB 0
LB 1.00 Hz
GB 0
PC 1.40

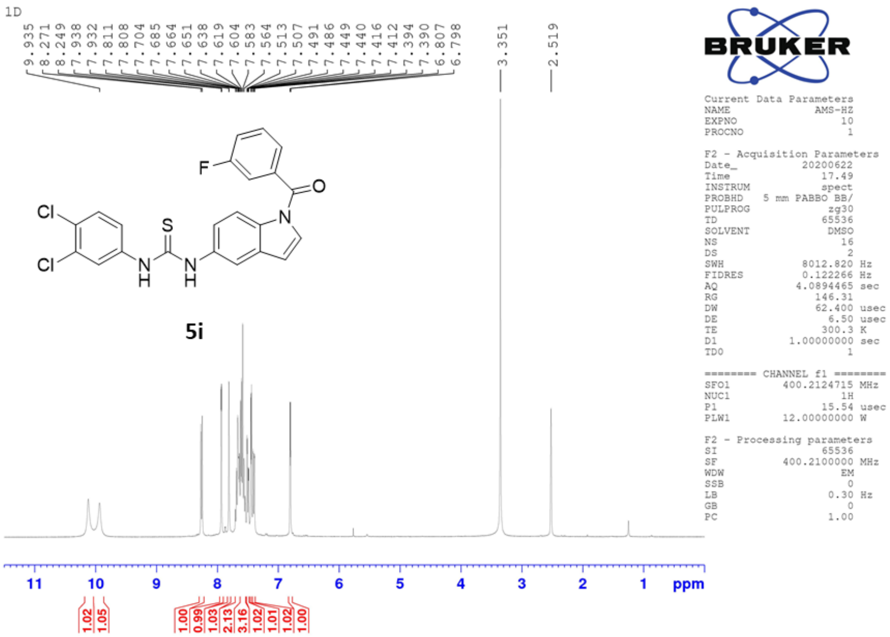

1D
9.935
8.271
8.249
7.938
7.932
7.811
7.808
7.704
7.685
7.664
7.651
7.638
7.619
7.604
7.583
7.564
7.513
7.507
7.491
7.486
7.449
7.440
7.416
7.412
7.394
7.390
6.807
6.798
3.351
2.519
F
O
N
Cl
Cl
S
N
H
N
H
5i
11 10 9 8 7 6 5 4 3 2 1 ppm
1.02 1.05 1.00 0.99 1.03 2.13 3.16 1.02 1.01 1.02 1.00
BRUKER
Current Data Parameters
NAME AMS-HZ
EXPNO 10
PROCNO 1
F2 - Acquisition Parameters
Date_ 20200622
Time 17.49
INSTRUM spect
PROBHD 5 mm PABBO BB/
PULPROG zg30
TD 65536
SOLVENT DMSO
NS 16
DS 2
SWH 8012.820 Hz
FIDRES 0.122266 Hz
AQ 4.0894465 sec
RG 146.31
DW 62.400 usec
DE 6.50 usec
TE 300.3 K
D1 1.00000000 sec
TD0 1
======== CHANNEL f1 ========
SFO1 400.2124715 MHz
NUC1 1H
P1 15.54 usec
PLW1 12.00000000 W
F2 - Processing parameters
SI 65536
SF 400.2100000 MHz
WDW EM
SSB 0
LB 0.30 Hz
GB 0
PC 1.00

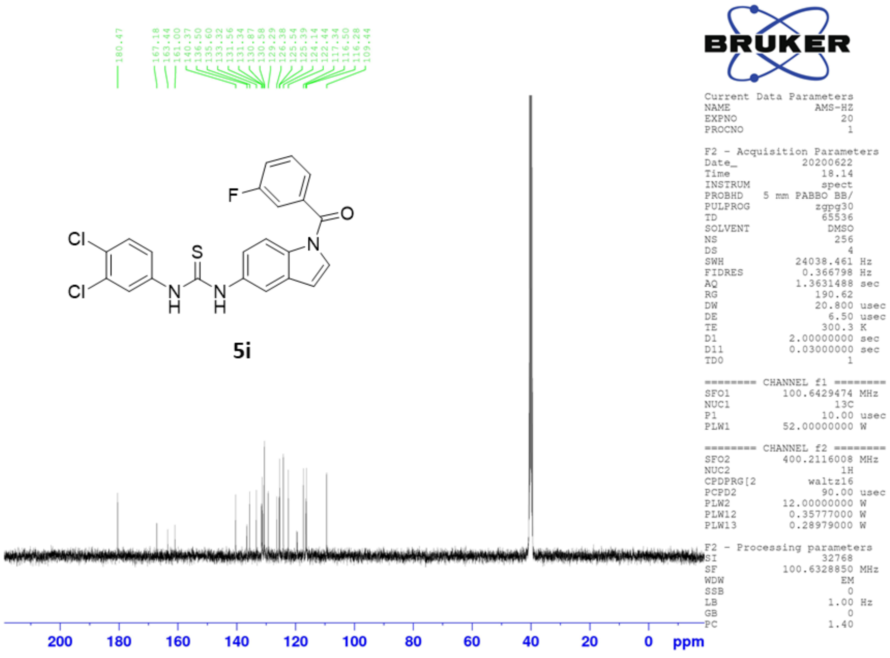

180.47
167.18
163.44
161.00
140.37
136.50
135.60
133.32
131.56
131.34
130.87
130.58
129.29
126.38
125.54
125.39
124.14
122.44
117.34
116.50
116.28
109.44
F
O
N
Cl
Cl
S
N
H
N
H
5i
200 180 160 140 120 100 80 60 40 20 0 ppm
BRUKER
Current Data Parameters
NAME AMS-HZ
EXPNO 20
PROCNO 1
F2 - Acquisition Parameters
Date_ 20200622
Time 18.14
INSTRUM spect
PROBHD 5 mm PABBO BB/
PULPROG zgpg30
TD 65536
SOLVENT DMSO
NS 256
DS 4
SWH 24038.461 Hz
FIDRES 0.366798 Hz
AQ 1.3631488 sec
RG 190.62
DW 20.800 usec
DE 6.50 usec
TE 300.3 K
D1 2.00000000 sec
D11 0.03000000 sec
TD0 1
======== CHANNEL f1 ========
SFO1 100.6429474 MHz
NUC1 13C
P1 10.00 usec
PLW1 52.00000000 W
======== CHANNEL f2 ========
SFO2 400.2116008 MHz
NUC2 1H
CPDPRG[2 waltz16
PCPD2 90.00 usec
PLW2 12.00000000 W
PLW12 0.35777000 W
PLW13 0.28979000 W
F2 - Processing parameters
SI 32768
SF 100.6328850 MHz
WDW EM
SSB 0
LB 1.00 Hz
GB 0
PC 1.40

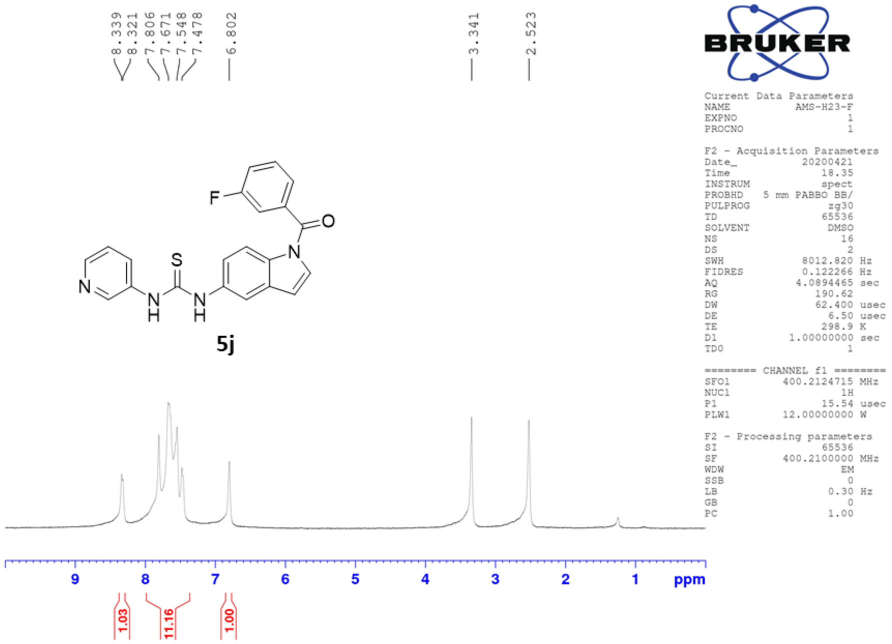
8.339
8.321
7.806
7.671
7.548
7.478
6.802
3.341
2.523
BRUKER
Current Data Parameters
NAME AMS-H23-F
EXPNO 1
PROCNO 1
F2 - Acquisition Parameters
Date_ 20200421
Time 18.35
INSTRUM spect
PROBHD 5 mm PABBO BB/
PULPROG zg30
TD 65536
SOLVENT DMSO
NS 16
DS 2
SWH 8012.820 Hz
FIDRES 0.122266 Hz
AQ 4.0894465 sec
RG 190.62
DW 62.400 usec
DE 6.50 usec
TE 298.9 K
D1 1.00000000 sec
TD0 1
======== CHANNEL f1 ========
SFO1 400.2124715 MHz
NUC1 1H
P1 15.54 usec
PLW1 12.00000000 W
F2 - Processing parameters
SI 65536
SF 400.2100000 MHz
WDW EM
SSB 0
LB 0.30 Hz
GB 0
PC 1.00
F
O
N
S
N
N
H
N
H
5j
9
8
7
6
5
4
3
2
1
ppm
1.03
11.16
1.00

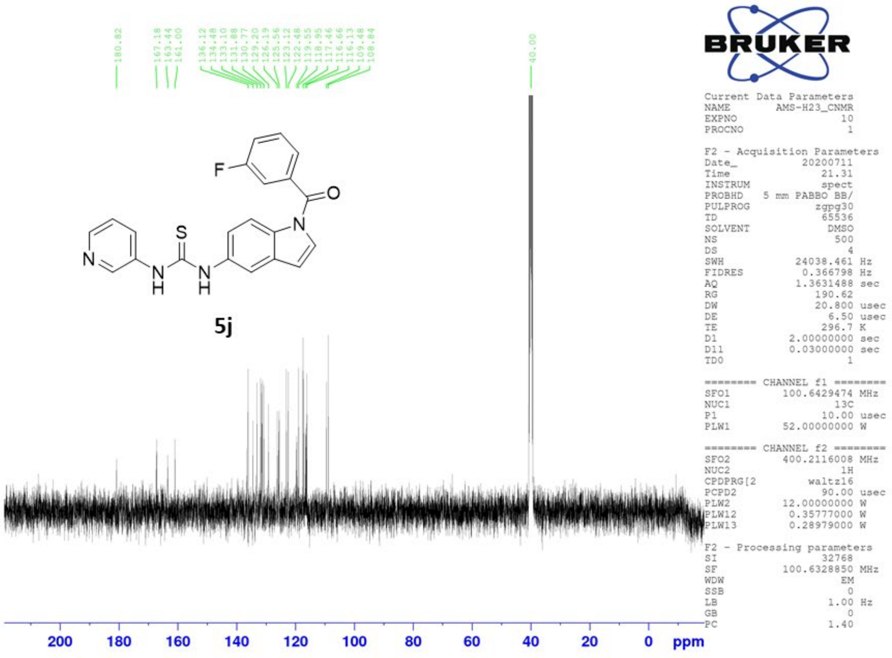
180.82
167.18
163.44
161.00
136.12
134.48
133.10
131.88
130.77
129.20
126.19
125.56
123.12
122.48
119.55
118.95
117.46
116.65
116.13
109.48
108.84
40.00
BRUKER
Current Data Parameters
NAME AMS-H23_CNMR
EXPNO 10
PROCNO 1
F2 - Acquisition Parameters
Date_ 20200711
Time 21.31
INSTRUM spect
PROBHD 5 mm PABBO BB/
PULPROG zgpg30
TD 65536
SOLVENT DMSO
NS 500
DS 4
SWH 24038.461 Hz
FIDRES 0.366798 Hz
AQ 1.3631488 sec
RG 190.62
DW 20.800 usec
DE 6.50 usec
TE 296.7 K
D1 2.00000000 sec
D11 0.03000000 sec
TD0 1
======== CHANNEL f1 ========
SFO1 100.6429474 MHz
NUC1 13C
P1 10.00 usec
PLW1 52.00000000 W
======== CHANNEL f2 ========
SFO2 400.2116008 MHz
NUC2 1H
CPDPRG[2 waltz16
PCPD2 90.00 usec
PLW2 12.00000000 W
PLW12 0.35777000 W
PLW13 0.28979000 W
F2 - Processing parameters
SI 32768
SF 100.6328850 MHz
WDW EM
SSB 0
LB 1.00 Hz
GB 0
PC 1.40
F
O
N
S
N
N
H
N
H
5j
200
180
160
140
120
100
80
60
40
20
0
ppm

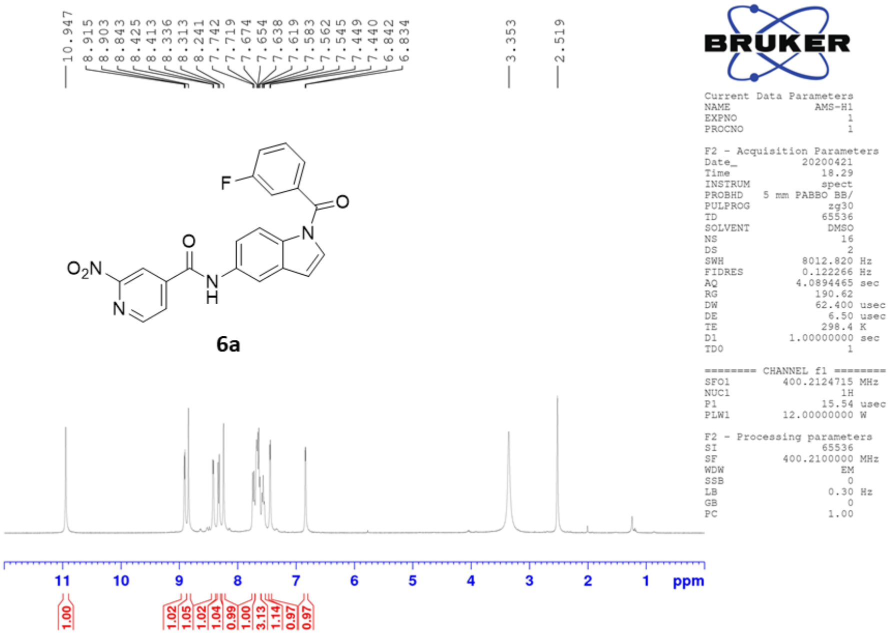
10.947
8.915
8.903
8.843
8.425
8.413
8.336
8.313
8.241
7.742
7.719
7.674
7.654
7.638
7.619
7.583
7.562
7.545
7.449
7.440
6.842
6.834
3.353
2.519
F
O
O
O_2N
N
N
H
N
6a
11
10
9
8
7
6
5
4
3
2
1
ppm
1.00
1.02
1.05
1.02
1.04
0.99
1.00
3.13
1.14
0.97
0.97
BRUKER
Current Data Parameters
NAME AMS-H1
EXPNO 1
PROCNO 1
F2 - Acquisition Parameters
Date_ 20200421
Time 18.29
INSTRUM spect
PROBHD 5 mm PABBO BB/
PULPROG zg30
TD 65536
SOLVENT DMSO
NS 16
DS 2
SWH 8012.820 Hz
FIDRES 0.122266 Hz
AQ 4.0894465 sec
RG 190.62
DW 62.400 usec
DE 6.50 usec
TE 298.4 K
D1 1.00000000 sec
TD0 1
======== CHANNEL f1 ========
SFO1 400.2124715 MHz
NUC1 1H
P1 15.54 usec
PLW1 12.00000000 W
F2 - Processing parameters
SI 65536
SF 400.2100000 MHz
WDW EM
SSB 0
LB 0.30 Hz
GB 0
PC 1.00

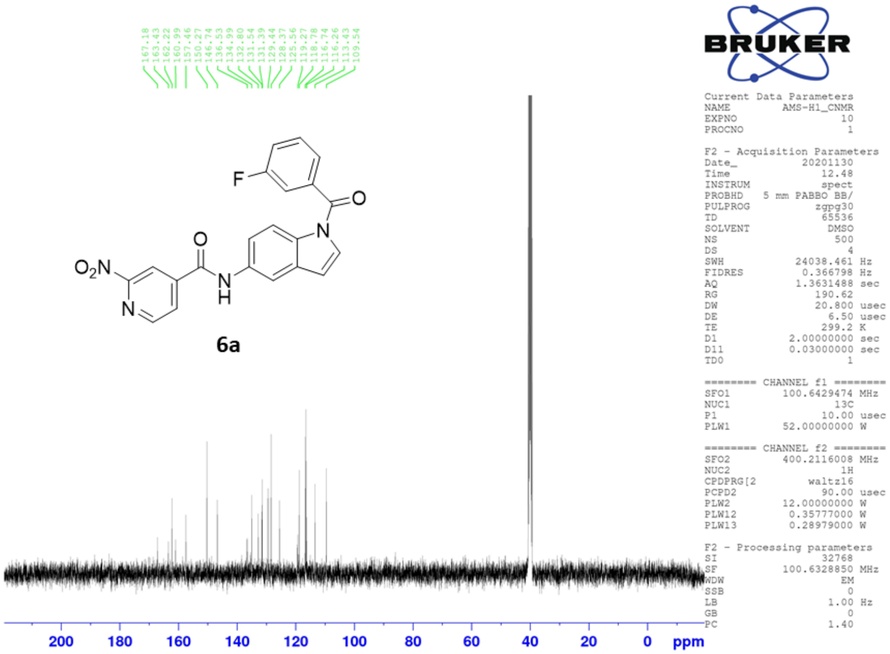
167.18
163.43
162.22
160.99
157.46
150.27
146.74
136.53
134.99
132.80
131.54
131.39
129.44
128.37
125.56
119.27
118.78
116.74
116.26
113.43
109.54
F
O
O
O_2N
N
N
H
N
6a
200
180
160
140
120
100
80
60
40
20
0
ppm
BRUKER
Current Data Parameters
NAME AMS-H1_CNMR
EXPNO 10
PROCNO 1
F2 - Acquisition Parameters
Date_ 20201130
Time 12.48
INSTRUM spect
PROBHD 5 mm PABBO BB/
PULPROG zgpg30
TD 65536
SOLVENT DMSO
NS 500
DS 4
SWH 24038.461 Hz
FIDRES 0.366798 Hz
AQ 1.3631488 sec
RG 190.62
DW 20.800 usec
DE 6.50 usec
TE 299.2 K
D1 2.00000000 sec
D11 0.03000000 sec
TD0 1
======== CHANNEL f1 ========
SFO1 100.6429474 MHz
NUC1 13C
P1 10.00 usec
PLW1 52.00000000 W
======== CHANNEL f2 ========
SFO2 400.2116008 MHz
NUC2 1H
CPDPRG[2 waltz16
PCPD2 90.00 usec
PLW2 12.00000000 W
PLW12 0.35777000 W
PLW13 0.28979000 W
F2 - Processing parameters
SI 32768
SF 100.6328850 MHz
WDW EM
SSB 0
LB 1.00 Hz
GB 0
PC 1.40

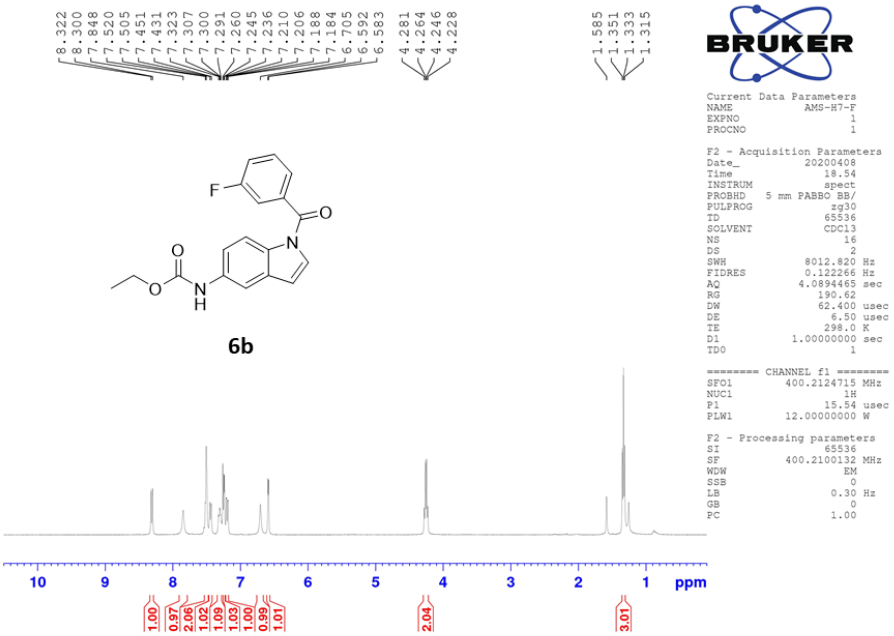
8.322 8.300 7.848 7.520 7.505 7.451 7.431 7.323 7.307 7.300 7.291 7.260 7.245 7.236 7.210 7.206 7.188 7.184 6.705 6.592 6.583
4.281 4.264 4.246 4.228
1.585 1.351 1.333 1.315
BRUKER
F
O
N
O
O
N
H
6b
Current Data Parameters
NAME AMS-H7-F
EXPNO 1
PROCNO 1
F2 - Acquisition Parameters
Date_ 20200408
Time 18.54
INSTRUM spect
PROBHD 5 mm PABBO BB/
PULPROG zg30
TD 65536
SOLVENT CDCl3
NS 16
DS 2
SWH 8012.820 Hz
FIDRES 0.122266 Hz
AQ 4.0894465 sec
RG 190.62
DW 62.400 usec
DE 6.50 usec
TE 298.0 K
D1 1.00000000 sec
TD0 1
======== CHANNEL f1 ========
SFO1 400.2124715 MHz
NUC1 1H
P1 15.54 usec
PLW1 12.00000000 W
F2 - Processing parameters
SI 65536
SF 400.2100132 MHz
WDW EM
SSB 0
LB 0.30 Hz
GB 0
PC 1.00
10 9 8 7 6 5 4 3 2 1 ppm
1.00 0.97 2.06 1.02 1.09 1.03 1.00 0.99 1.01
2.04
3.01

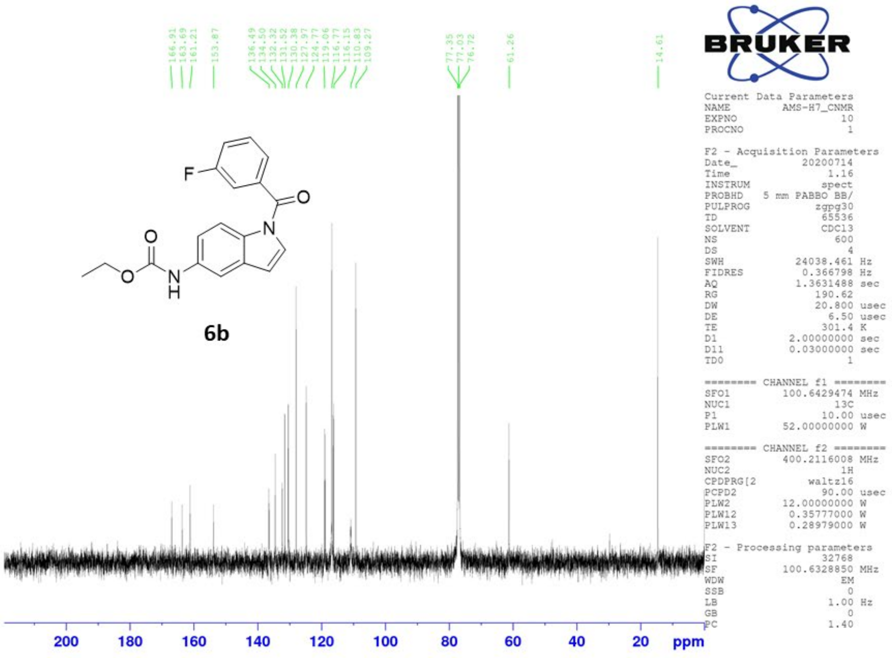
166.91 163.69 161.21 153.87
136.49 134.50 132.32 131.52 130.38 127.97 124.77 119.26 116.77 116.15 110.83 109.27
77.35 77.03 76.72
61.26
14.61
BRUKER
F
O
N
O
O
N
H
6b
Current Data Parameters
NAME AMS-H7_CNMR
EXPNO 10
PROCNO 1
F2 - Acquisition Parameters
Date_ 20200714
Time 1.16
INSTRUM spect
PROBHD 5 mm PABBO BB/
PULPROG zgpg30
TD 65536
SOLVENT CDCl3
NS 600
DS 4
SWH 24038.461 Hz
FIDRES 0.366798 Hz
AQ 1.3631488 sec
RG 190.62
DW 20.800 usec
DE 6.50 usec
TE 301.4 K
D1 2.00000000 sec
D11 0.03000000 sec
TD0 1
======== CHANNEL f1 ========
SFO1 100.6429474 MHz
NUC1 13C
P1 10.00 usec
PLW1 52.00000000 W
======== CHANNEL f2 ========
SFO2 400.2116008 MHz
NUC2 1H
CPDPRG[2 waltz16
PCPD2 90.00 usec
PLW2 12.00000000 W
PLW12 0.35777000 W
PLW13 0.28979000 W
F2 - Processing parameters
SI 32768
SF 100.6328850 MHz
WDW EM
SSB 0
LB 1.00 Hz
GB 0
PC 1.40
200 180 160 140 120 100 80 60 40 20 ppm

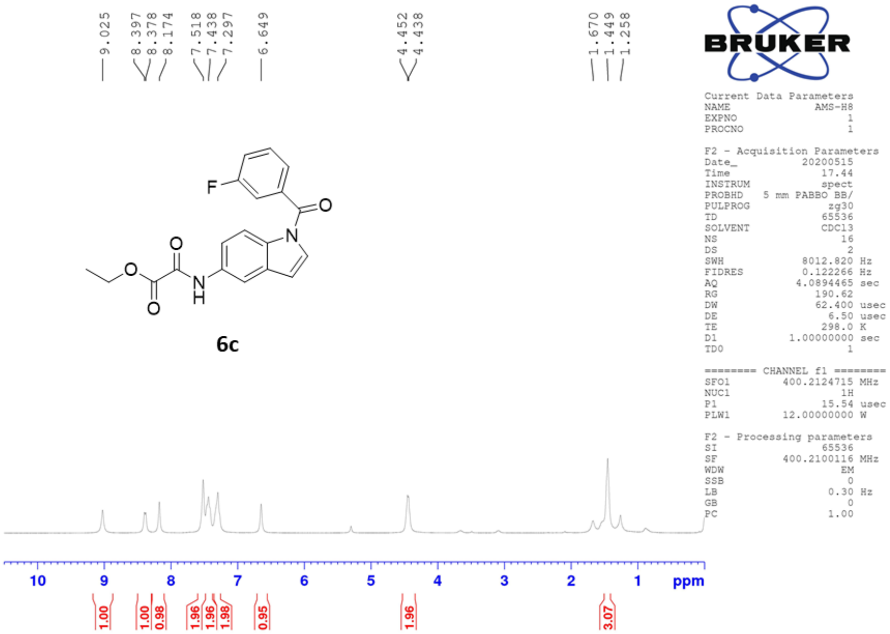
9.025
8.397
8.378
8.174
7.518
7.438
7.297
6.649
4.452
4.438
1.670
1.449
1.258
F
O
N
O
O
N
H
O
6c
BRUKER
Current Data Parameters
NAME AMS-H8
EXPNO 1
PROCNO 1
F2 - Acquisition Parameters
Date_ 20200515
Time 17.44
INSTRUM spect
PROBHD 5 mm PABBO BB/
PULPROG zg30
TD 65536
SOLVENT CDCl3
NS 16
DS 2
SWH 8012.820 Hz
FIDRES 0.122266 Hz
AQ 4.0894465 sec
RG 190.62
DW 62.400 usec
DE 6.50 usec
TE 298.0 K
D1 1.00000000 sec
TD0 1
======== CHANNEL f1 ========
SFO1 400.2124715 MHz
NUC1 1H
P1 15.54 usec
PLW1 12.00000000 W
F2 - Processing parameters
SI 65536
SF 400.2100116 MHz
WDW EM
SSB 0
LB 0.30 Hz
GB 0
PC 1.00
10 9 8 7 6 5 4 3 2 1 ppm
1.00
1.00
0.98
1.96
1.96
1.98
0.95
1.96
3.07

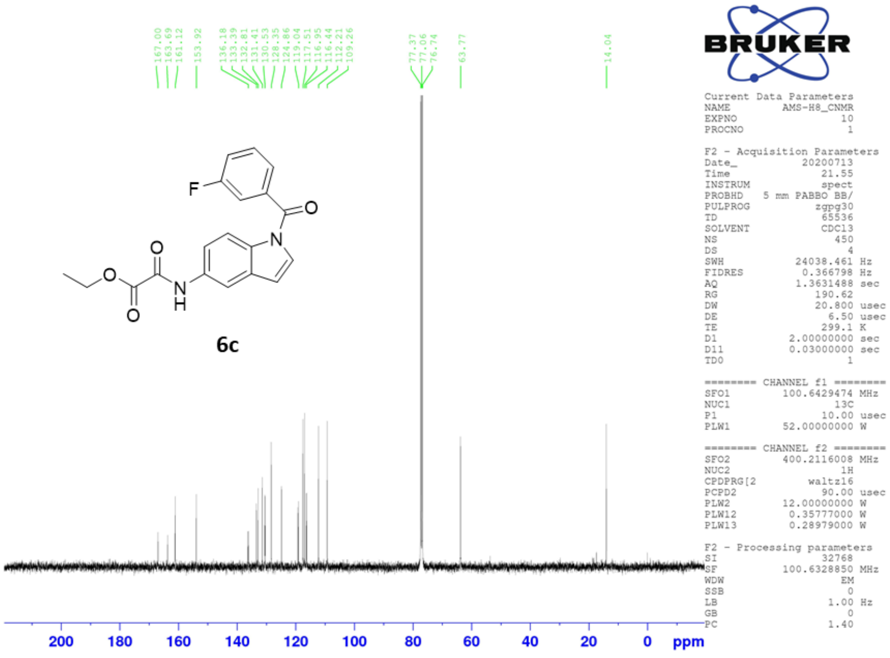
167.00
163.69
161.12
153.92
136.18
133.39
132.81
131.41
130.53
128.35
124.86
119.84
117.51
116.95
116.44
112.21
109.26
77.37
77.06
76.74
63.77
14.04
F
O
N
O
O
N
H
O
6c
BRUKER
Current Data Parameters
NAME AMS-H8_CNMR
EXPNO 10
PROCNO 1
F2 - Acquisition Parameters
Date_ 20200713
Time 21.55
INSTRUM spect
PROBHD 5 mm PABBO BB/
PULPROG zgpg30
TD 65536
SOLVENT CDCl3
NS 450
DS 4
SWH 24038.461 Hz
FIDRES 0.366798 Hz
AQ 1.3631488 sec
RG 190.62
DW 20.800 usec
DE 6.50 usec
TE 299.1 K
D1 2.00000000 sec
D11 0.03000000 sec
TD0 1
======== CHANNEL f1 ========
SFO1 100.6429474 MHz
NUC1 13C
P1 10.00 usec
PLW1 52.00000000 W
======== CHANNEL f2 ========
SFO2 400.2116008 MHz
NUC2 1H
CPDPRG[2 waltz16
PCPD2 90.00 usec
PLW2 12.00000000 W
PLW12 0.35777000 W
PLW13 0.28979000 W
F2 - Processing parameters
SI 32768
SF 100.6328850 MHz
WDW EM
SSB 0
LB 1.00 Hz
GB 0
PC 1.40
200 180 160 140 120 100 80 60 40 20 0 ppm

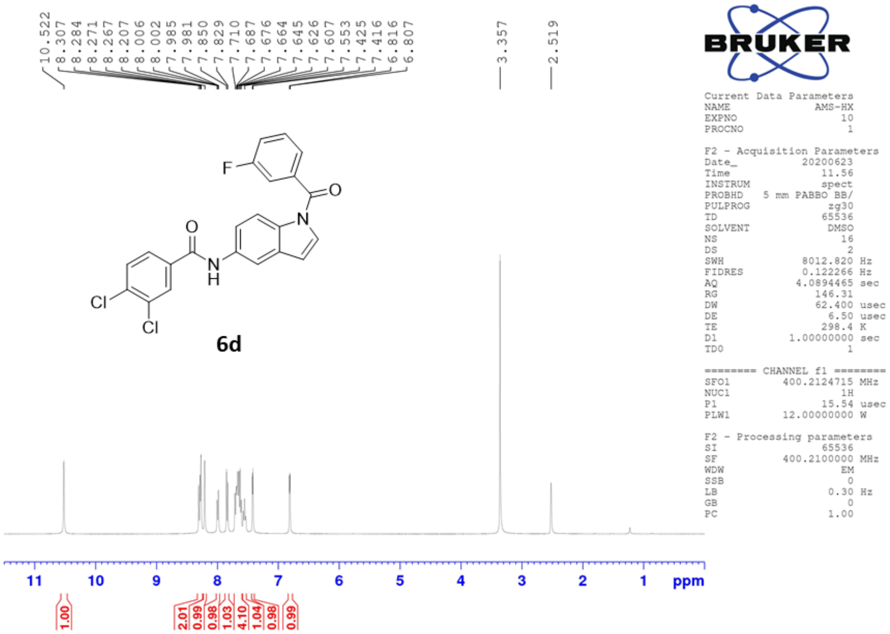
10.522
8.307
8.284
8.271
8.267
8.207
8.006
8.002
7.985
7.981
7.850
7.829
7.710
7.687
7.676
7.664
7.645
7.626
7.607
7.553
7.425
7.416
6.816
6.807
3.357
2.519
F
O
N
O
N
H
Cl
Cl
6d
BRUKER
Current Data Parameters
NAME AMS-HX
EXPNO 10
PROCNO 1
F2 - Acquisition Parameters
Date_ 20200623
Time 11.56
INSTRUM spect
PROBHD 5 mm PABBO BB/
PULPROG zg30
TD 65536
SOLVENT DMSO
NS 16
DS 2
SWH 8012.820 Hz
FIDRES 0.122266 Hz
AQ 4.0894465 sec
RG 146.31
DW 62.400 usec
DE 6.50 usec
TE 298.4 K
D1 1.00000000 sec
TD0 1
======== CHANNEL f1 ========
SFO1 400.2124715 MHz
NUC1 1H
P1 15.54 usec
PLW1 12.00000000 W
F2 - Processing parameters
SI 65536
SF 400.2100000 MHz
WDW EM
SSB 0
LB 0.30 Hz
GB 0
PC 1.00
11 10 9 8 7 6 5 4 3 2 1 ppm
1.00
2.01
0.99
0.98
1.03
4.10
1.04
0.98
0.99

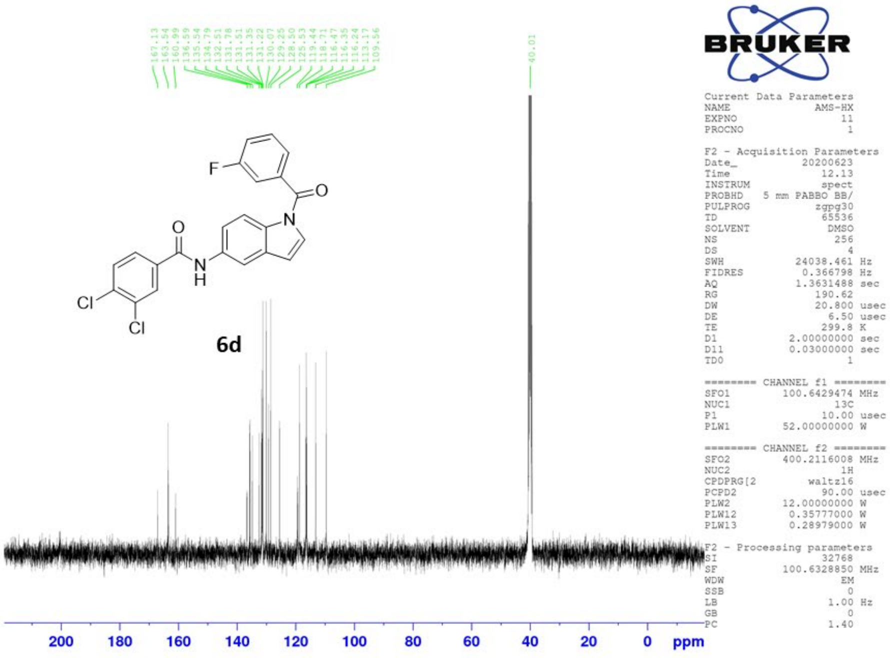
167.13
163.54
160.99
136.59
135.54
134.79
132.51
131.78
131.51
131.35
131.22
130.07
129.25
128.50
125.53
119.44
118.71
116.47
116.35
116.24
113.17
109.56
40.01
F
O
N
O
N
H
Cl
Cl
6d
BRUKER
Current Data Parameters
NAME AMS-HX
EXPNO 11
PROCNO 1
F2 - Acquisition Parameters
Date_ 20200623
Time 12.13
INSTRUM spect
PROBHD 5 mm PABBO BB/
PULPROG zgpg30
TD 65536
SOLVENT DMSO
NS 256
DS 4
SWH 24038.461 Hz
FIDRES 0.366798 Hz
AQ 1.3631488 sec
RG 190.62
DW 20.800 usec
DE 6.50 usec
TE 299.8 K
D1 2.00000000 sec
D11 0.03000000 sec
TD0 1
======== CHANNEL f1 ========
SFO1 100.6429474 MHz
NUC1 13C
P1 10.00 usec
PLW1 52.00000000 W
======== CHANNEL f2 ========
SFO2 400.2116008 MHz
NUC2 1H
CPDPRG[2 waltz16
PCPD2 90.00 usec
PLW2 12.00000000 W
PLW12 0.35777000 W
PLW13 0.28979000 W
F2 - Processing parameters
SI 32768
SF 100.6328850 MHz
WDW EM
SSB 0
LB 1.00 Hz
GB 0
PC 1.40
200 180 160 140 120 100 80 60 40 20 0 ppm

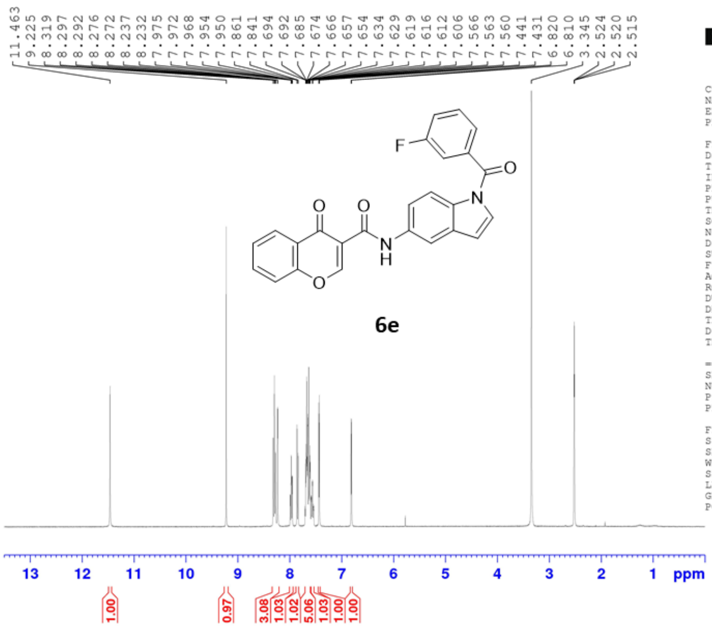


```
Current Data Parameters
NAME       AMS-H111_conc
EXPNO                 10
PROCNO                 1

F2 - Acquisition Parameters
Date_           20200903
Time               16.34
INSTRUM            spect
PROBHD   5 mm PABBO BB/
PULPROG             zg30
TD                 65536
SOLVENT             DMSO
NS                    16
DS                     2
SWH          8012.820 Hz
FIDRES       0.122266 Hz
AQ          4.0894465 sec
RG                190.62
DW             62.400 usec
DE               6.50 usec
TE               297.5 K
D1        1.00000000 sec
TD0                    1

======== CHANNEL f1 ========
SFO1     400.2124715 MHz
NUC1                  1H
P1             15.54 usec
PLW1     12.00000000 W

F2 - Processing parameters
SI                 65536
SF        400.2100000 MHz
WDW                   EM
SSB                    0
LB               0.30 Hz
GB                     0
PC                  1.00
```

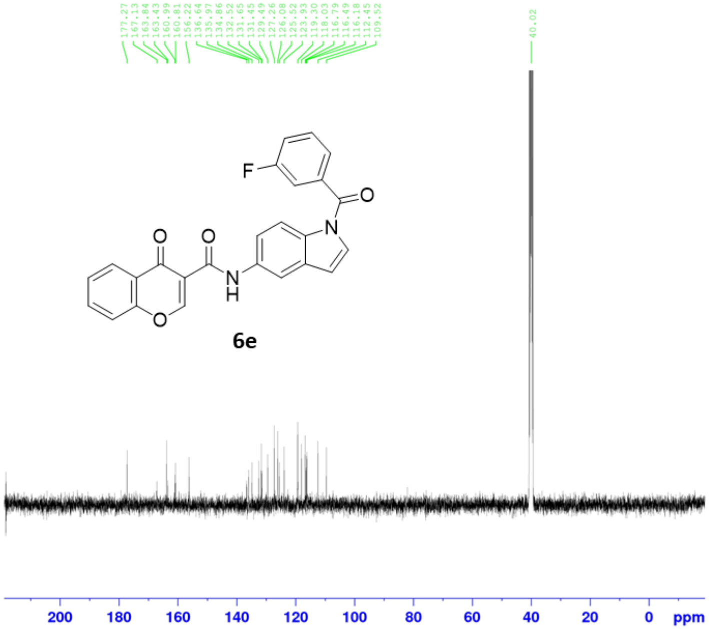


```
Current Data Parameters
NAME            4e_CNMR
EXPNO                 10
PROCNO                 1

F2 - Acquisition Parameters
Date_           20210101
Time                2.50
INSTRUM            spect
PROBHD   5 mm PABBO BB/
PULPROG           zgpg30
TD                 65536
SOLVENT             DMSO
NS                  4000
DS                     4
SWH         24038.461 Hz
FIDRES       0.366798 Hz
AQ          1.3631488 sec
RG                190.62
DW             20.800 usec
DE               6.50 usec
TE               299.8 K
D1        2.00000000 sec
D11       0.03000000 sec
TD0                    1

======== CHANNEL f1 ========
SFO1     100.6429474 MHz
NUC1                 13C
P1             10.00 usec
PLW1     52.00000000 W

======== CHANNEL f2 ========
SFO2     400.2116008 MHz
NUC2                  1H
CPDPRG[2         waltz16
PCPD2          90.00 usec
PLW2     12.00000000 W
PLW12     0.35777000 W
PLW13     0.28979000 W

F2 - Processing parameters
SI                 32768
SF        100.6328850 MHz
WDW                   EM
SSB      0
LB               1.00 Hz
GB       0
PC                  1.40
```

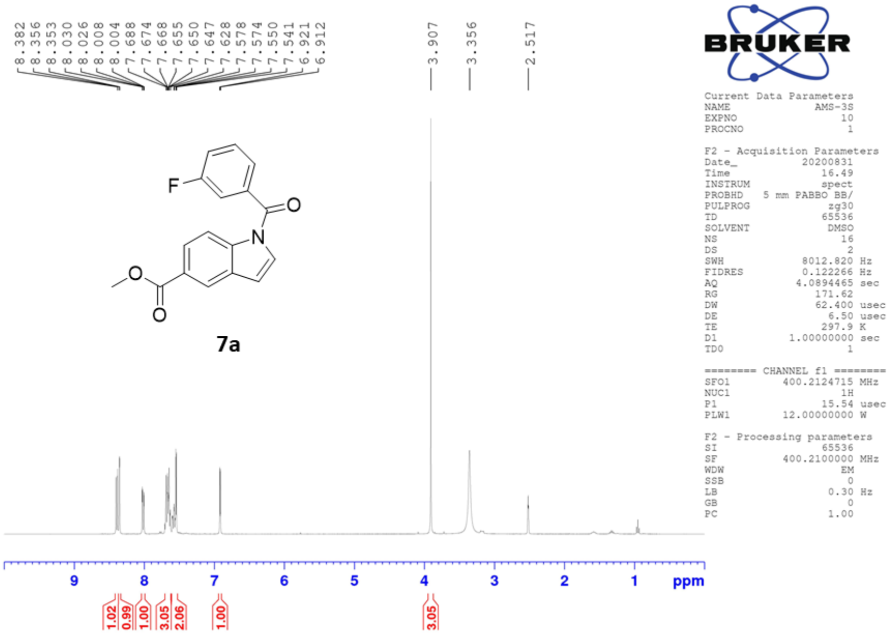
8.382
8.356
8.353
8.030
8.026
8.008
8.004
7.688
7.674
7.668
7.655
7.650
7.647
7.628
7.578
7.574
7.550
7.541
6.921
6.912
3.907
3.356
2.517
F
O
N
O
O
7a
9
8
7
6
5
4
3
2
1
ppm
1.02
0.99
1.00
3.05
2.06
1.00
3.05
BRUKER
Current Data Parameters
NAME AMS-3S
EXPNO 10
PROCNO 1
F2 - Acquisition Parameters
Date_ 20200831
Time 16.49
INSTRUM spect
PROBHD 5 mm PABBO BB/
PULPROG zg30
TD 65536
SOLVENT DMSO
NS 16
DS 2
SWH 8012.820 Hz
FIDRES 0.122266 Hz
AQ 4.0894465 sec
RG 171.62
DW 62.400 usec
DE 6.50 usec
TE 297.9 K
D1 1.00000000 sec
TD0 1
======== CHANNEL f1 ========
SFO1 400.2124715 MHz
NUC1 1H
P1 15.54 usec
PLW1 12.00000000 W
F2 - Processing parameters
SI 65536
SF 400.2100000 MHz
WDW EM
SSB 0
LB 0.30 Hz
GB 0
PC 1.00

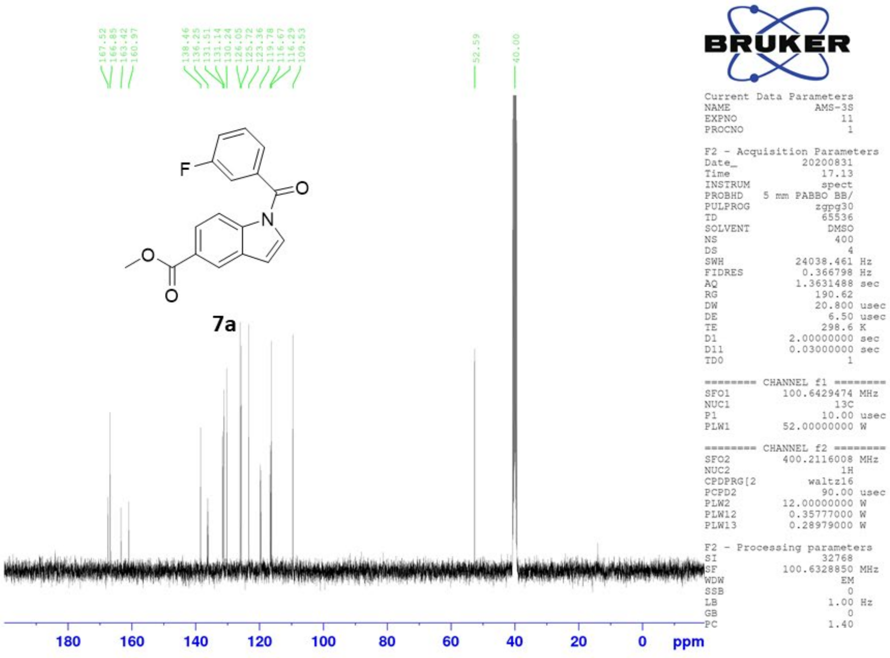
167.52
166.85
163.42
160.97
138.46
136.25
131.51
131.14
130.24
126.05
125.72
123.36
119.78
116.67
116.29
109.53
52.59
40.00
F
O
N
O
O
7a
180
160
140
120
100
80
60
40
20
0
ppm
BRUKER
Current Data Parameters
NAME AMS-3S
EXPNO 11
PROCNO 1
F2 - Acquisition Parameters
Date_ 20200831
Time 17.13
INSTRUM spect
PROBHD 5 mm PABBO BB/
PULPROG zgpg30
TD 65536
SOLVENT DMSO
NS 400
DS 4
SWH 24038.461 Hz
FIDRES 0.366798 Hz
AQ 1.3631488 sec
RG 190.62
DW 20.800 usec
DE 6.50 usec
TE 298.6 K
D1 2.00000000 sec
D11 0.03000000 sec
TD0 1
======== CHANNEL f1 ========
SFO1 100.6429474 MHz
NUC1 13C
P1 10.00 usec
PLW1 52.00000000 W
======== CHANNEL f2 ========
SFO2 400.2116008 MHz
NUC2 1H
CPDPRG[2 waltz16
PCPD2 90.00 usec
PLW2 12.00000000 W
PLW12 0.35777000 W
PLW13 0.28979000 W
F2 - Processing parameters
SI 32768
SF 100.6328850 MHz
WDW EM
SSB 0
LB 1.00 Hz
GB 0
PC 1.40

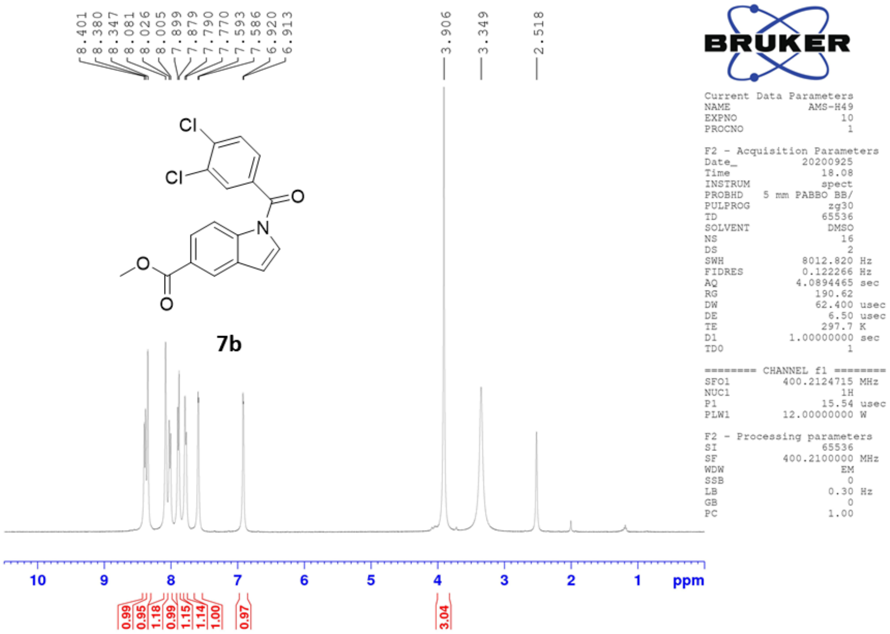
8.401
8.380
8.347
8.081
8.026
8.005
7.899
7.879
7.790
7.770
7.593
7.586
6.920
6.913
3.906
3.349
2.518
Cl
Cl
O
N
O
O
7b
10 9 8 7 6 5 4 3 2 1 ppm
0.99 0.95 1.18 0.99 1.15 1.14 1.00 0.97 3.04
BRUKER
Current Data Parameters
NAME AMS-H49
EXPNO 10
PROCNO 1
F2 - Acquisition Parameters
Date_ 20200925
Time 18.08
INSTRUM spect
PROBHD 5 mm PABBO BB/
PULPROG zg30
TD 65536
SOLVENT DMSO
NS 16
DS 2
SWH 8012.820 Hz
FIDRES 0.122266 Hz
AQ 4.0894465 sec
RG 190.62
DW 62.400 usec
DE 6.50 usec
TE 297.7 K
D1 1.00000000 sec
TD0 1
======== CHANNEL f1 ========
SFO1 400.2124715 MHz
NUC1 1H
P1 15.54 usec
PLW1 12.00000000 W
F2 - Processing parameters
SI 65536
SF 400.2100000 MHz
WDW EM
SSB 0
LB 0.30 Hz
GB 0
PC 1.00

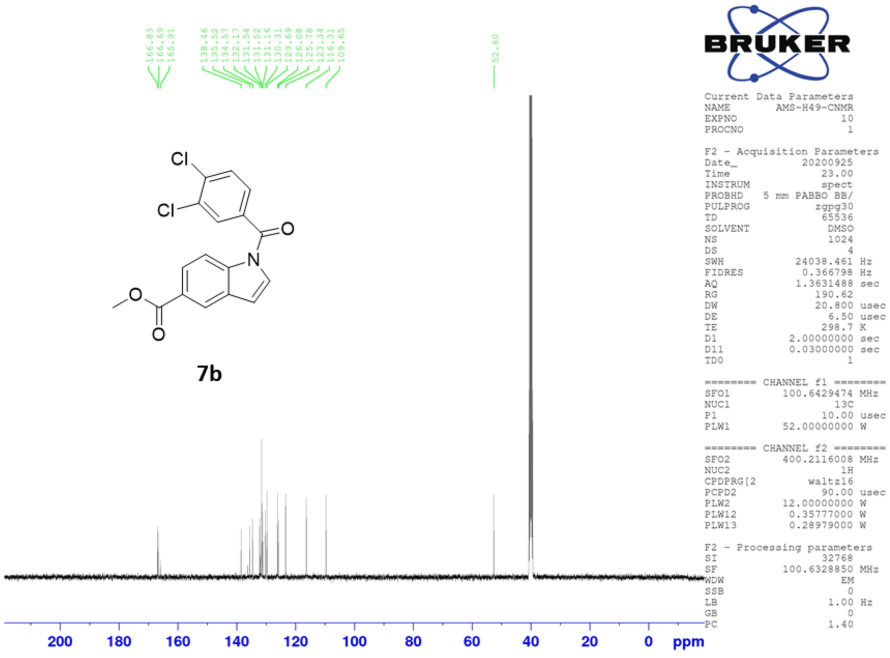
166.83
166.69
165.91
138.46
135.52
134.57
132.17
131.54
131.52
131.16
130.31
129.69
126.08
125.78
123.34
116.31
109.65
52.60
Cl
Cl
O
N
O
O
7b
200 180 160 140 120 100 80 60 40 20 0 ppm
BRUKER
Current Data Parameters
NAME AMS-H49-CNMR
EXPNO 10
PROCNO 1
F2 - Acquisition Parameters
Date_ 20200925
Time 23.00
INSTRUM spect
PROBHD 5 mm PABBO BB/
PULPROG zgpg30
TD 65536
SOLVENT DMSO
NS 1024
DS 4
SWH 24038.461 Hz
FIDRES 0.366798 Hz
AQ 1.3631488 sec
RG 190.62
DW 20.800 usec
DE 6.50 usec
TE 298.7 K
D1 2.00000000 sec
D11 0.03000000 sec
TD0 1
======== CHANNEL f1 ========
SFO1 100.6429474 MHz
NUC1 13C
P1 10.00 usec
PLW1 52.00000000 W
======== CHANNEL f2 ========
SFO2 400.2116008 MHz
NUC2 1H
CPDPRG[2 waltz16
PCPD2 90.00 usec
PLW2 12.00000000 W
PLW12 0.35777000 W
PLW13 0.28979000 W
F2 - Processing parameters
SI 32768
SF 100.6328850 MHz
WDW EM
SSB 0
LB 1.00 Hz
GB 0
PC 1.40

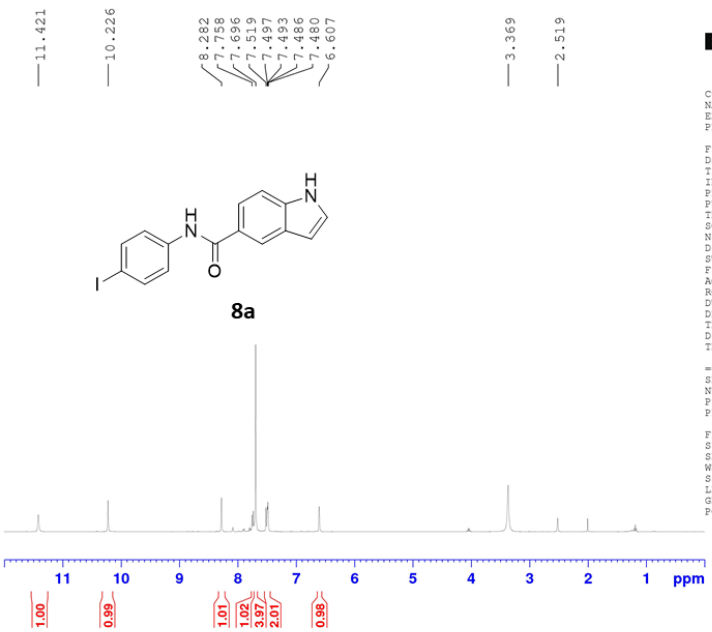


```
Current Data Parameters
NAME          A-H41 (2nd)
EXPNO                  10
PROCNO                  1

F2 - Acquisition Parameters
Date_            20201020
Time                20.05
INSTRUM             spect
PROBHD   5 mm PABBO BB/
PULPROG              zg30
TD                  65536
SOLVENT              DMSO
NS                     16
DS                      2
SWH              8012.820 Hz
FIDRES           0.122266 Hz
AQ              4.0894465 sec
RG                 146.31
DW                 62.400 usec
DE                   6.50 usec
TE                  298.1 K
D1             1.00000000 sec
TD0                     1

======== CHANNEL f1 ========
SFO1        400.2124715 MHz
NUC1                   1H
P1                  15.54 usec
PLW1          12.00000000 W

F2 - Processing parameters
SI                  65536
SF            400.2100000 MHz
WDW                    EM
SSB                     0
LB                   0.30 Hz
GB                      0
PC                   1.00
```

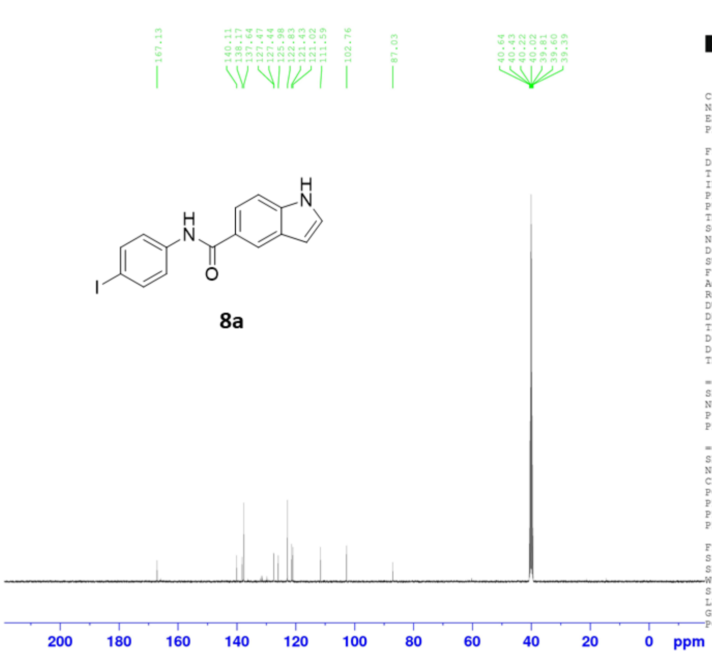


```
Current Data Parameters
NAME           A-H41_CNMR
EXPNO                  10
PROCNO                  1

F2 - Acquisition Parameters
Date_            20201022
Time                22.42
INSTRUM             spect
PROBHD   5 mm PABBO BB/
PULPROG            zgpg30
TD                  65536
SOLVENT              DMSO
NS                    700
DS                      4
SWH             24038.461 Hz
FIDRES           0.366798 Hz
AQ              1.3631488 sec
RG                 190.62
DW                 20.800 usec
DE                   6.50 usec
TE                  299.6 K
D1             2.00000000 sec
D11            0.03000000 sec
TD0                     1

======== CHANNEL f1 ========
SFO1        100.6429474 MHz
NUC1                  13C
P1                  10.00 usec
PLW1          52.00000000 W

======== CHANNEL f2 ========
SFO2        400.2116008 MHz
NUC2                   1H
CPDPRG[2          waltz16
PCPD2               90.00 usec
PLW2          12.00000000 W
PLW12          0.35777000 W
PLW13          0.28979000 W

F2 - Processing parameters
SI                  32768
SF            100.6328850 MHz
WDW                    EM
SSB                     0
LB                   1.00 Hz
GB                      0
PC                   1.40
```

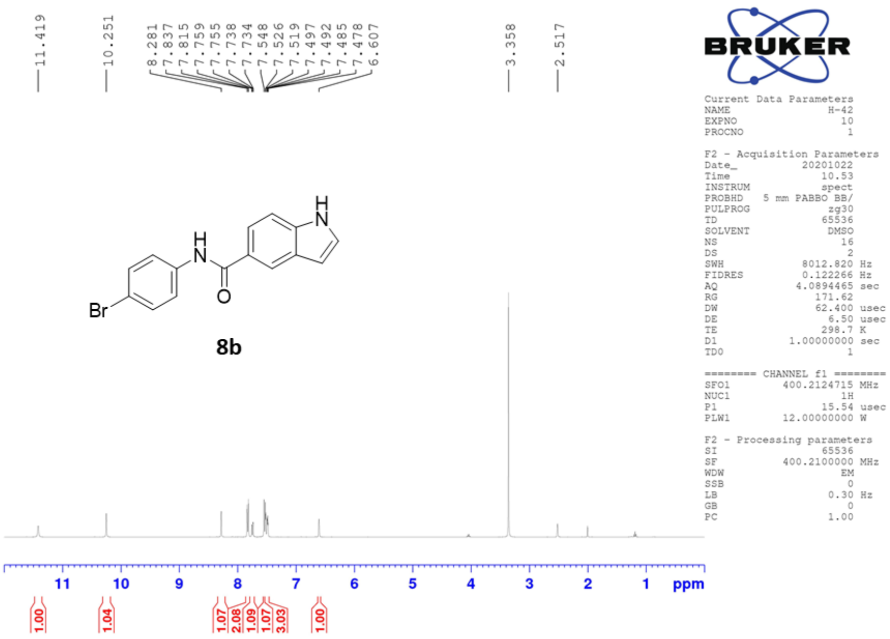
11.419
10.251
8.281
7.837
7.815
7.759
7.755
7.738
7.734
7.548
7.526
7.519
7.497
7.492
7.485
7.478
6.607
3.358
2.517
H
N
Br
O
11
10
9
8
7
6
5
4
3
2
1
ppm
1.00
1.04
1.07
2.08
1.09
1.07
3.03
1.00
BRUKER
Current Data Parameters
NAME H-42
EXPNO 10
PROCNO 1
F2 - Acquisition Parameters
Date_ 20201022
Time 10.53
INSTRUM spect
PROBHD 5 mm PABBO BB/
PULPROG zg30
TD 65536
SOLVENT DMSO
NS 16
DS 2
SWH 8012.820 Hz
FIDRES 0.122266 Hz
AQ 4.0894465 sec
RG 171.62
DW 62.400 usec
DE 6.50 usec
TE 298.7 K
D1 1.00000000 sec
TD0 1
======== CHANNEL f1 ========
SFO1 400.2124715 MHz
NUC1 1H
P1 15.54 usec
PLW1 12.00000000 W
F2 - Processing parameters
SI 65536
SF 400.2100000 MHz
WDW EM
SSB 0
LB 0.30 Hz
GB 0
PC 1.00

8b

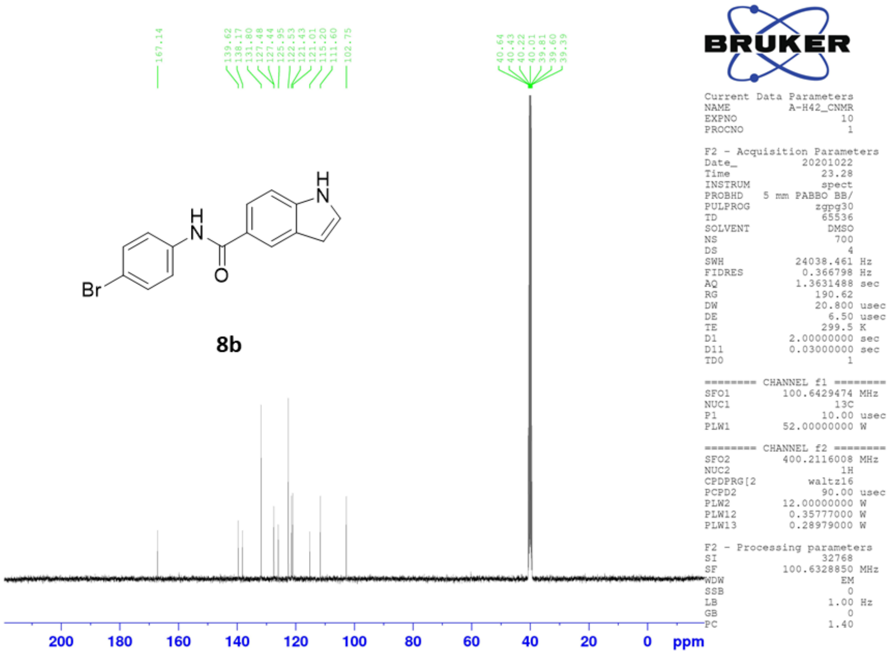
167.14
139.62
138.17
131.80
127.48
127.44
125.95
122.53
121.63
121.01
115.20
111.60
102.75
40.64
40.43
40.22
40.01
39.81
39.60
39.39
H
N
Br
O
200
180
160
140
120
100
80
60
40
20
0
ppm
BRUKER
Current Data Parameters
NAME A-H42_CNMR
EXPNO 10
PROCNO 1
F2 - Acquisition Parameters
Date_ 20201022
Time 23.28
INSTRUM spect
PROBHD 5 mm PABBO BB/
PULPROG zgpg30
TD 65536
SOLVENT DMSO
NS 700
DS 4
SWH 24038.461 Hz
FIDRES 0.366798 Hz
AQ 1.3631488 sec
RG 190.62
DW 20.800 usec
DE 6.50 usec
TE 299.5 K
D1 2.00000000 sec
D11 0.03000000 sec
TD0 1
======== CHANNEL f1 ========
SFO1 100.6429474 MHz
NUC1 13C
P1 10.00 usec
PLW1 52.00000000 W
======== CHANNEL f2 ========
SFO2 400.2116008 MHz
NUC2 1H
CPDPRG[2 waltz16
PCPD2 90.00 usec
PLW2 12.00000000 W
PLW12 0.35777000 W
PLW13 0.28979000 W
F2 - Processing parameters
SI 32768
SF 100.6328850 MHz
WDW EM
SSB 0
LB 1.00 Hz
GB 0
PC 1.40

8b

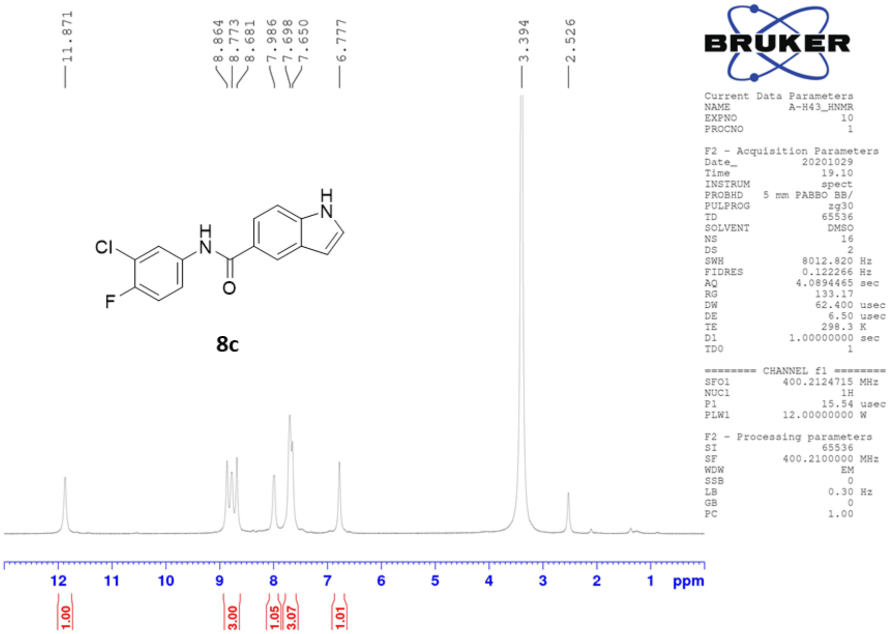
11.871
8.864
8.773
8.681
7.986
7.698
7.650
6.777
3.394
2.526
BRUKER
Cl
F
H
N
O
NH
8c
Current Data Parameters
NAME A-H43_HNMR
EXPNO 10
PROCNO 1
F2 - Acquisition Parameters
Date_ 20201029
Time 19.10
INSTRUM spect
PROBHD 5 mm PABBO BB/
PULPROG zg30
TD 65536
SOLVENT DMSO
NS 16
DS 2
SWH 8012.820 Hz
FIDRES 0.122266 Hz
AQ 4.0894465 sec
RG 133.17
DW 62.400 usec
DE 6.50 usec
TE 298.3 K
D1 1.00000000 sec
TD0 1
======== CHANNEL f1 ========
SFO1 400.2124715 MHz
NUC1 1H
P1 15.54 usec
PLW1 12.00000000 W
F2 - Processing parameters
SI 65536
SF 400.2100000 MHz
WDW EM
SSB 0
LB 0.30 Hz
GB 0
PC 1.00
12
11
10
9
8
7
6
5
4
3
2
1
ppm
1.00
3.00
1.05
3.07
1.01

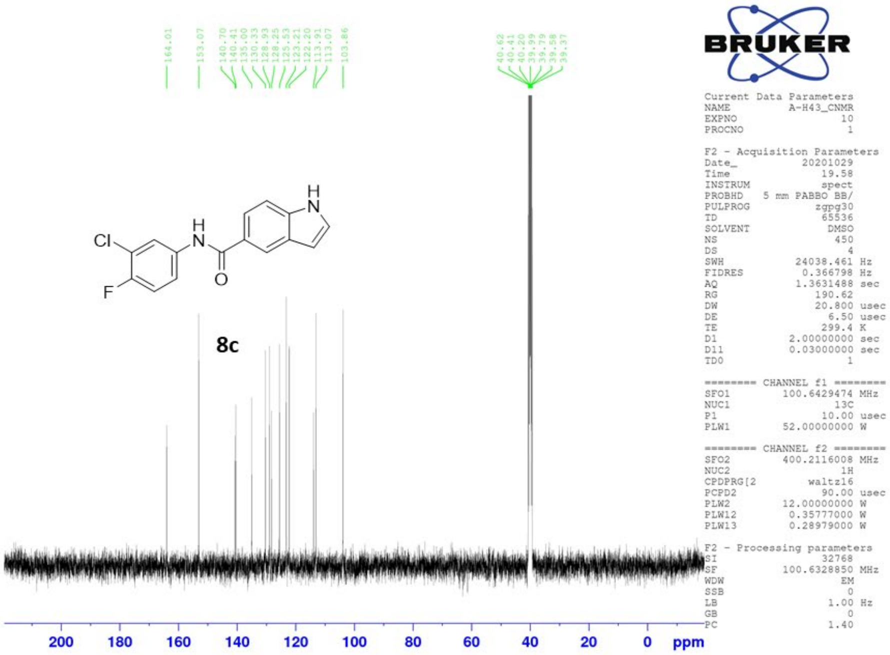
164.01
153.07
140.70
140.41
135.60
130.33
128.93
125.25
123.53
123.21
122.20
113.91
113.07
103.86
40.62
40.41
40.20
39.99
39.79
39.58
39.37
BRUKER
Cl
F
H
N
O
NH
8c
Current Data Parameters
NAME A-H43_CNMR
EXPNO 10
PROCNO 1
F2 - Acquisition Parameters
Date_ 20201029
Time 19.58
INSTRUM spect
PROBHD 5 mm PABBO BB/
PULPROG zgpg30
TD 65536
SOLVENT DMSO
NS 450
DS 4
SWH 24038.461 Hz
FIDRES 0.366798 Hz
AQ 1.3631488 sec
RG 190.62
DW 20.800 usec
DE 6.50 usec
TE 299.4 K
D1 2.00000000 sec
D11 0.03000000 sec
TD0 1
======== CHANNEL f1 ========
SFO1 100.6429474 MHz
NUC1 13C
P1 10.00 usec
PLW1 52.00000000 W
======== CHANNEL f2 ========
SFO2 400.2116008 MHz
NUC2 1H
CPDPRG[2 waltz16
PCPD2 90.00 usec
PLW2 12.00000000 W
PLW12 0.35777000 W
PLW13 0.28979000 W
F2 - Processing parameters
SI 32768
SF 100.6328850 MHz
WDW EM
SSB 0
LB 1.00 Hz
GB 0
PC 1.40
200
180
160
140
120
100
80
60
40
20
0
ppm

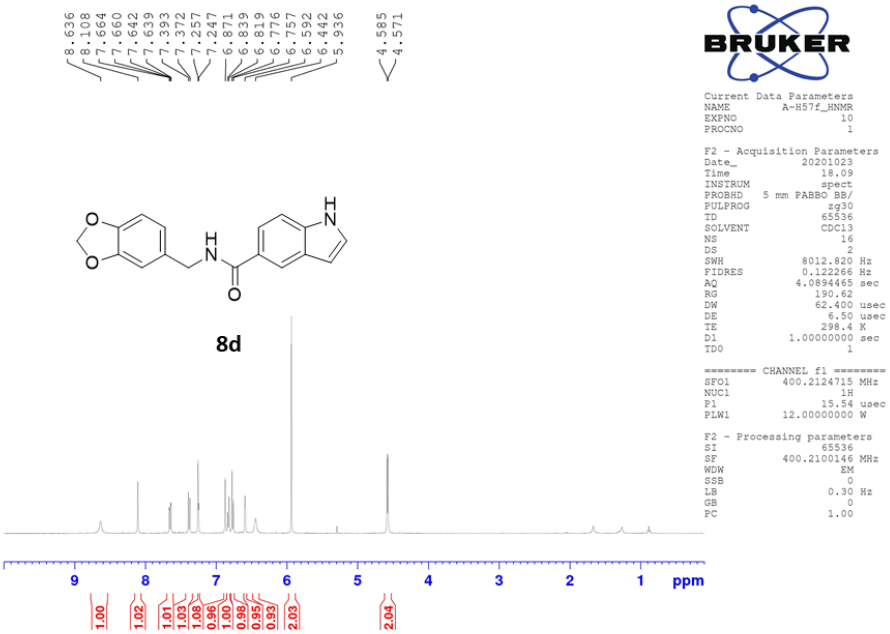
8.636
8.108
7.664
7.660
7.642
7.639
7.393
7.372
7.257
7.247
6.871
6.839
6.819
6.776
6.757
6.592
6.442
5.936
4.585
4.571
8d
9 8 7 6 5 4 3 2 1 ppm
1.00
1.02
1.01
1.03
1.08
0.96
1.00
0.98
0.95
0.93
2.03
2.04
BRUKER
Current Data Parameters
NAME A-H57f_HNMR
EXPNO 10
PROCNO 1
F2 - Acquisition Parameters
Date_ 20201023
Time 18.09
INSTRUM spect
PROBHD 5 mm PABBO BB/
PULPROG zg30
TD 65536
SOLVENT CDCl3
NS 16
DS 2
SWH 8012.820 Hz
FIDRES 0.122266 Hz
AQ 4.0894465 sec
RG 190.62
DW 62.400 usec
DE 6.50 usec
TE 298.4 K
D1 1.00000000 sec
TD0 1
======== CHANNEL f1 ========
SFO1 400.2124715 MHz
NUC1 1H
P1 15.54 usec
PLW1 12.00000000 W
F2 - Processing parameters
SI 65536
SF 400.2100146 MHz
WDW EM
SSB 0
LB 0.30 Hz
GB 0
PC 1.00

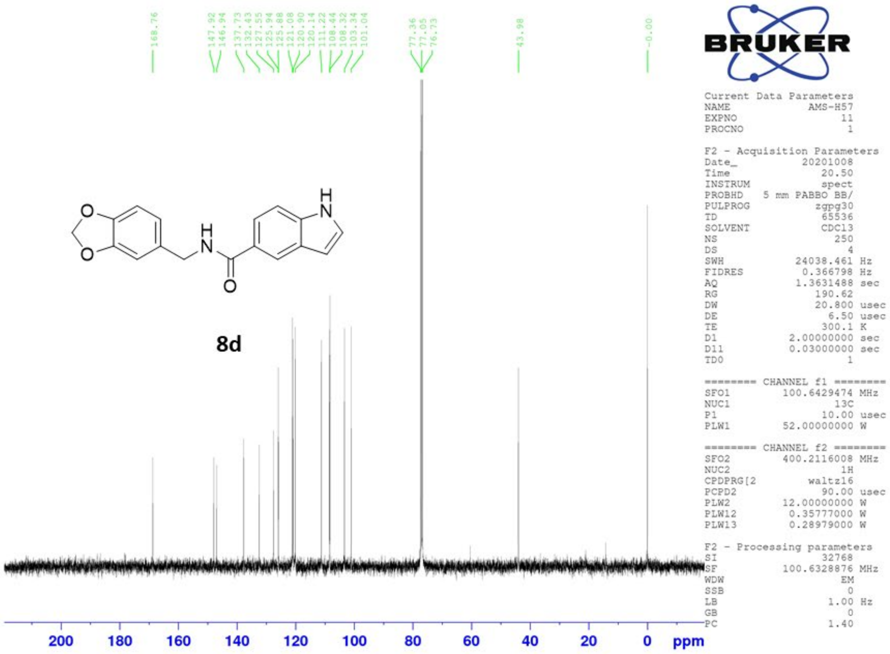
168.76
147.92
146.94
137.73
132.43
127.55
125.94
125.88
121.08
120.90
120.14
111.22
108.44
108.32
103.34
101.04
77.36
77.05
76.73
43.98
-0.00
8d
200 180 160 140 120 100 80 60 40 20 0 ppm
BRUKER
Current Data Parameters
NAME AMS-H57
EXPNO 11
PROCNO 1
F2 - Acquisition Parameters
Date_ 20201008
Time 20.50
INSTRUM spect
PROBHD 5 mm PABBO BB/
PULPROG zgpg30
TD 65536
SOLVENT CDCl3
NS 250
DS 4
SWH 24038.461 Hz
FIDRES 0.366798 Hz
AQ 1.3631488 sec
RG 190.62
DW 20.800 usec
DE 6.50 usec
TE 300.1 K
D1 2.00000000 sec
D11 0.03000000 sec
TD0 1
======== CHANNEL f1 ========
SFO1 100.6429474 MHz
NUC1 13C
P1 10.00 usec
PLW1 52.00000000 W
======== CHANNEL f2 ========
SFO2 400.2116008 MHz
NUC2 1H
CPDPRG[2 waltz16
PCPD2 90.00 usec
PLW2 12.00000000 W
PLW12 0.35777000 W
PLW13 0.28979000 W
F2 - Processing parameters
SI 32768
SF 100.6328876 MHz
WDW EM
SSB 0
LB 1.00 Hz
GB 0
PC 1.40

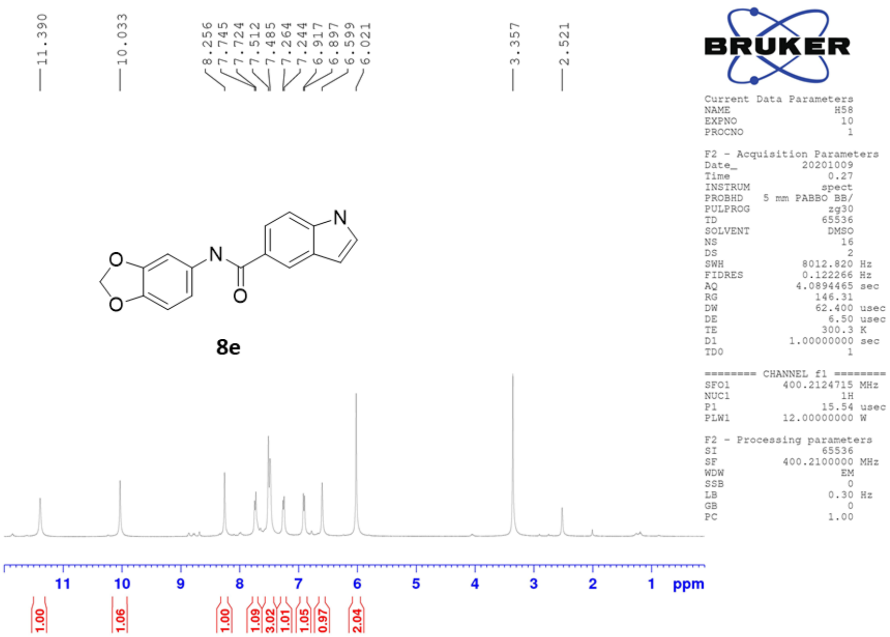
11.390
10.033
8.256
7.745
7.724
7.512
7.485
7.264
7.244
6.917
6.897
6.599
6.021
3.357
2.521
BRUKER
Current Data Parameters
NAME H58
EXPNO 10
PROCNO 1
F2 - Acquisition Parameters
Date_ 20201009
Time 0.27
INSTRUM spect
PROBHD 5 mm PABBO BB/
PULPROG zg30
TD 65536
SOLVENT DMSO
NS 16
DS 2
SWH 8012.820 Hz
FIDRES 0.122266 Hz
AQ 4.0894465 sec
RG 146.31
DW 62.400 usec
DE 6.50 usec
TE 300.3 K
D1 1.00000000 sec
TD0 1
======== CHANNEL f1 ========
SFO1 400.2124715 MHz
NUC1 1H
P1 15.54 usec
PLW1 12.00000000 W
F2 - Processing parameters
SI 65536
SF 400.2100000 MHz
WDW EM
SSB 0
LB 0.30 Hz
GB 0
PC 1.00
N
O
O
N
O
8e
11
10
9
8
7
6
5
4
3
2
1
ppm
1.00
1.06
1.00
1.09
3.02
1.01
1.05
0.97
2.04

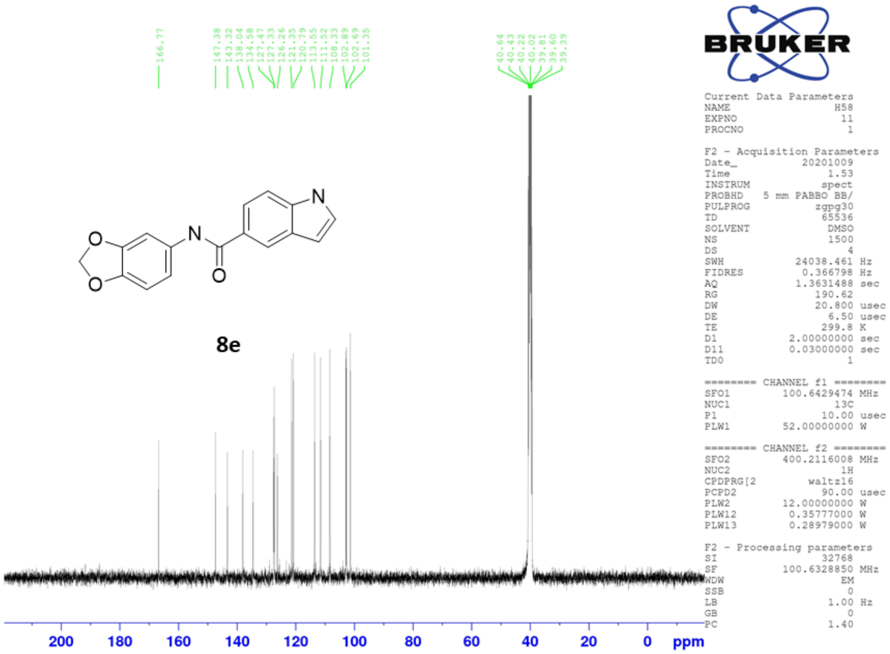
166.77
147.38
143.32
138.04
134.58
127.47
127.33
126.26
121.35
120.79
113.55
111.52
108.33
102.89
102.69
101.35
40.64
40.43
40.22
40.02
39.81
39.60
39.39
BRUKER
Current Data Parameters
NAME H58
EXPNO 11
PROCNO 1
F2 - Acquisition Parameters
Date_ 20201009
Time 1.53
INSTRUM spect
PROBHD 5 mm PABBO BB/
PULPROG zgpg30
TD 65536
SOLVENT DMSO
NS 1500
DS 4
SWH 24038.461 Hz
FIDRES 0.366798 Hz
AQ 1.3631488 sec
RG 190.62
DW 20.800 usec
DE 6.50 usec
TE 299.8 K
D1 2.00000000 sec
D11 0.03000000 sec
TD0 1
======== CHANNEL f1 ========
SFO1 100.6429474 MHz
NUC1 13C
P1 10.00 usec
PLW1 52.00000000 W
======== CHANNEL f2 ========
SFO2 400.2116008 MHz
NUC2 1H
CPDPRG[2 waltz16
PCPD2 90.00 usec
PLW2 12.00000000 W
PLW12 0.35777000 W
PLW13 0.28979000 W
F2 - Processing parameters
SI 32768
SF 100.6328850 MHz
WDW EM
SSB 0
LB 1.00 Hz
GB 0
PC 1.40
N
O
O
N
O
8e
200
180
160
140
120
100
80
60
40
20
0
ppm

2. Representative HPLC purity data of the compounds

	Name	Retention Time (min)	Purity1 Angle	Purity1 Threshold	PDA Match1 Spect. Name	PDA Match1 Angle	PDA Match1 Threshold	PDA Match1 Lib. Name	Area (μV*sec)	% Area	Height (μV)	Int Type	Amount
1		4.171							182884	1.44	36389	bb	
2		5.631							362117	2.86	67497	bb	
3		14.149							12135189	95.70	1702752	bb	

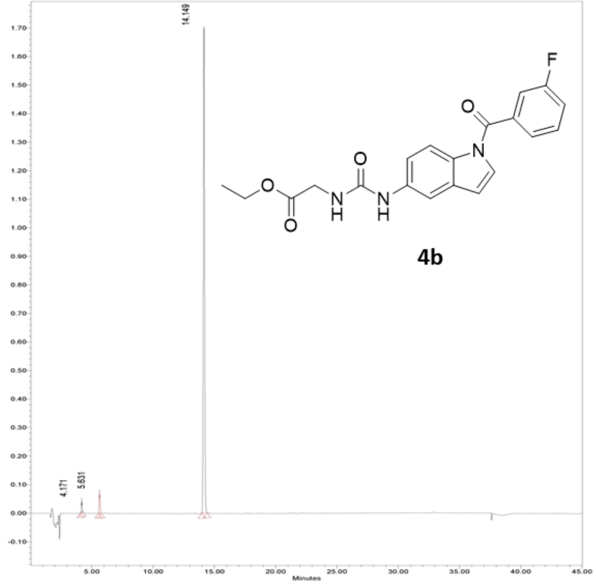

	Name	Retention Time (min)	Purity1 Angle	Purity1 Threshold	PDA Match1 Spect. Name	PDA Match1 Angle	PDA Match1 Threshold	PDA Match1 Lib. Name	Area (μV*sec)	% Area	Height (μV)	Int Type	Amount
1		11.528							722618	4.30	108944	bb	
2		12.746							34035	0.20	6370	bb	
3		18.282							16041627	95.50	2201606	bb	

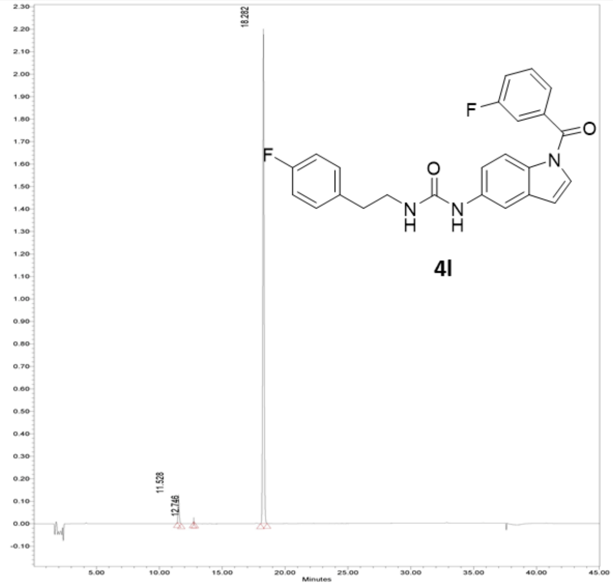

	Name	Retention Time (min)	Purity1 Angle	Purity1 Threshold	PDA Match1 Spect. Name	PDA Match1 Angle	PDA Match1 Threshold	PDA Match1 Lib. Name	Area (µV*sec)	% Area	Height (µV)	Int Type	Amount
1		9.569							84076	0.88	14431	bb	
2		17.348							9455947	99.12	1252820	bb	

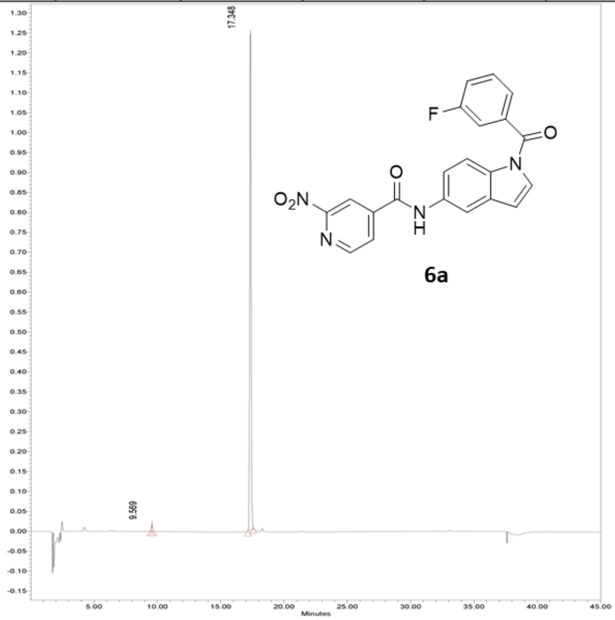

6a

	Name	Retention Time (min)	Purity1 Angle	Purity1 Threshold	PDA Match1 Spect. Name	PDA Match1 Angle	PDA Match1 Threshold	PDA Match1 Lib. Name	Area (µV*sec)	% Area	Height (µV)	Int Type	Amount
1		11.807							143417	0.85	20959	bb	
2		12.907							36312	0.21	4101	bb	
3		16.628							16480558	97.34	2106271	bb	
4		21.830							271366	1.60	37619	bb	

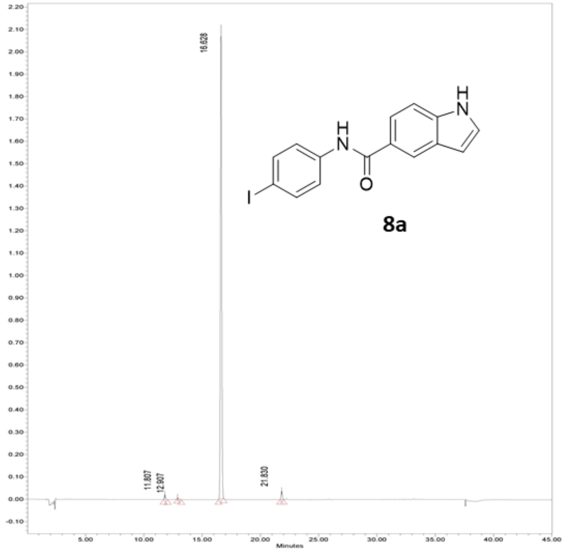

8a

	Name	Retention Time (min)	Purity1 Angle	Purity1 Threshold	PDA Match1 Spect. Name	PDA Match1 Angle	PDA Match1 Threshold	PDA Match1 Lib. Name	Area (µV*sec)	% Area	Height (µV)	Int Type	Amount
1		15.725							26481219	95.64	2461374	bb	
2		20.990							1206129	4.36	174283	bb	

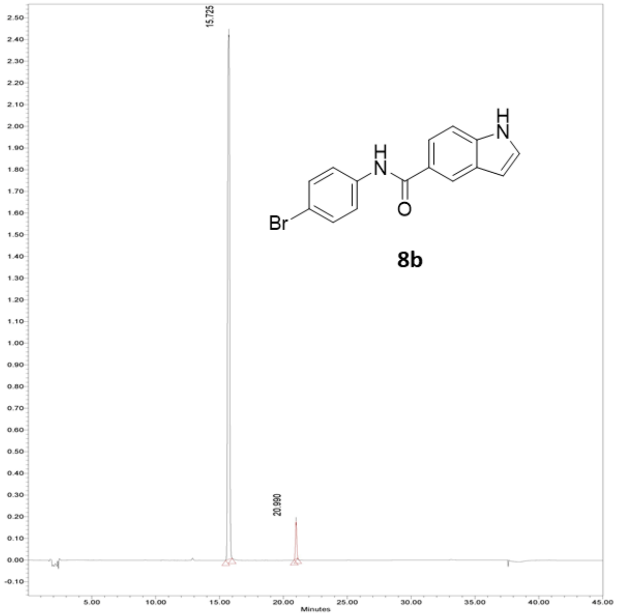

8b

	Name	Retention Time (min)	Purity1 Angle	Purity1 Threshold	PDA Match1 Spect. Name	PDA Match1 Angle	PDA Match1 Threshold	PDA Match1 Lib. Name	Area (µV*sec)	% Area	Height (µV)	Int Type	Amount
1		10.548							173366	1.05	27024	bb	
2		12.866							16023703	97.28	2134594	bb	
3		14.794							3519	0.02	838	bb	
4		15.765							2730	0.02	573	bb	
5		17.723							14939	0.09	2821	bb	
6		19.510							15872	0.10	2458	bb	
7		21.026							41357	0.25	6726	bb	
8		25.096							196114	1.19	27752	bb	

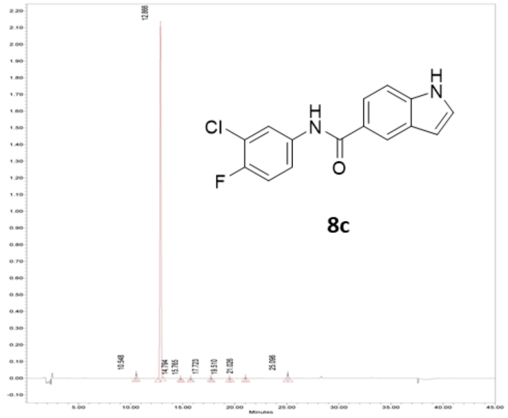

8c

3. Representative HRMS data of the compounds

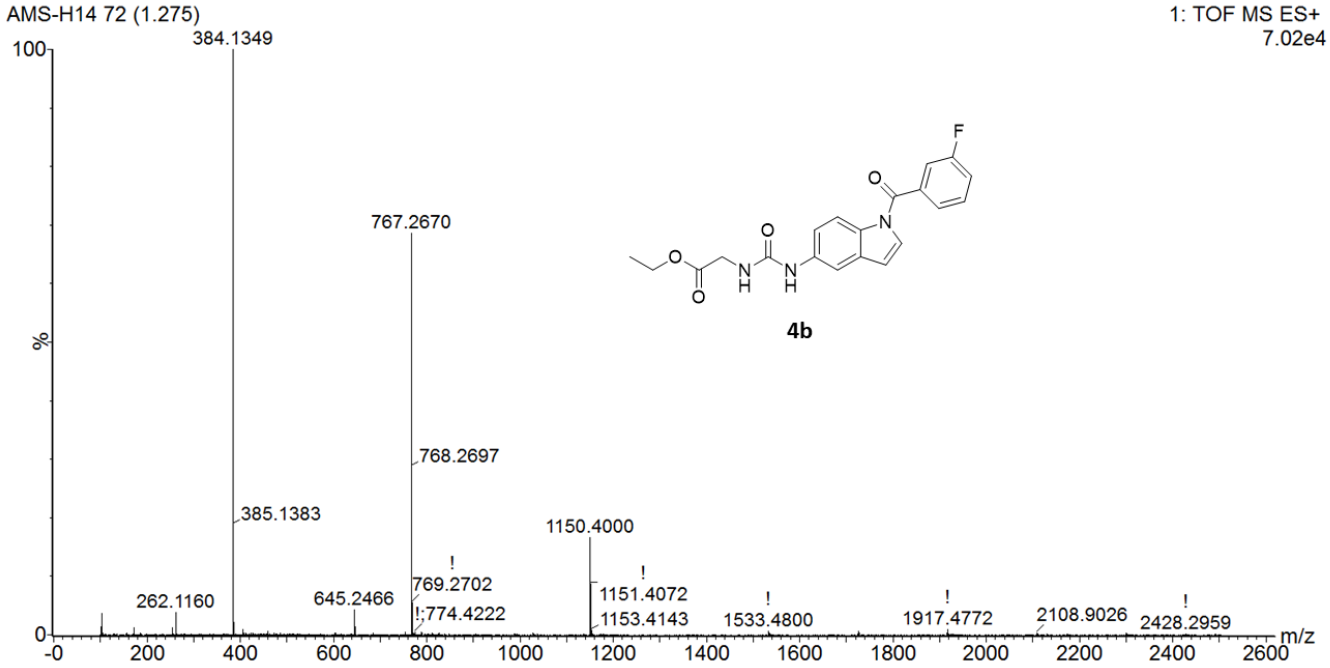

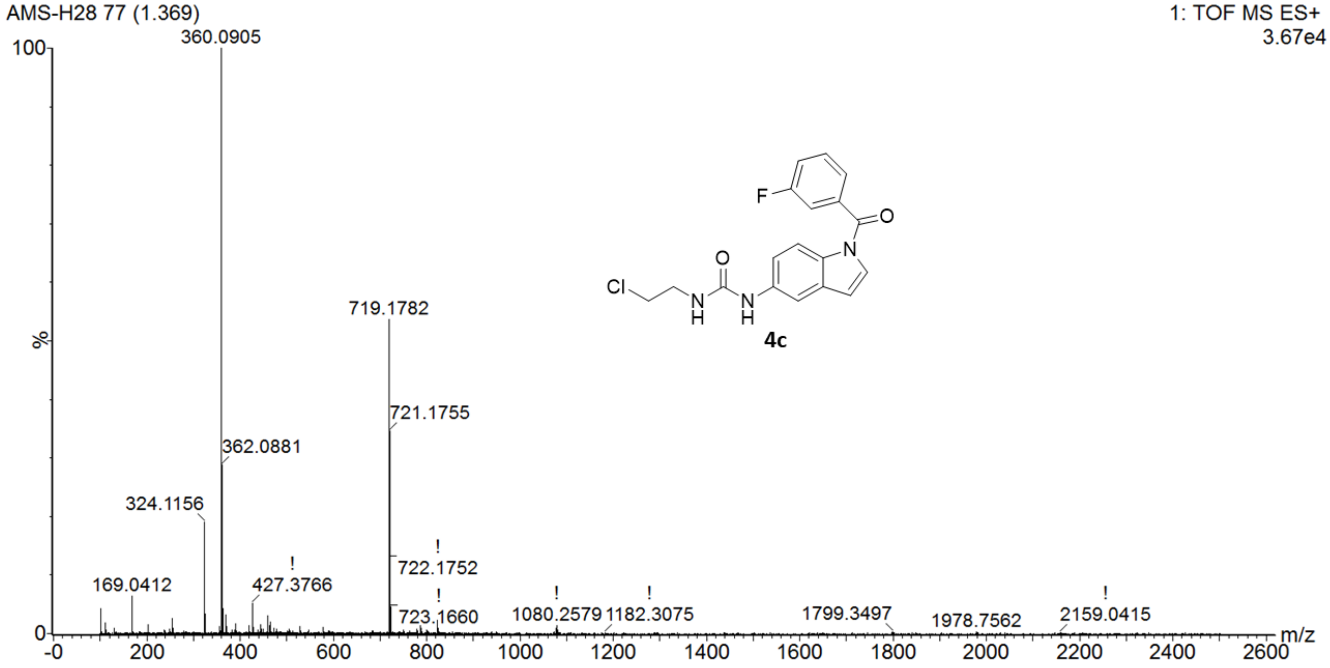

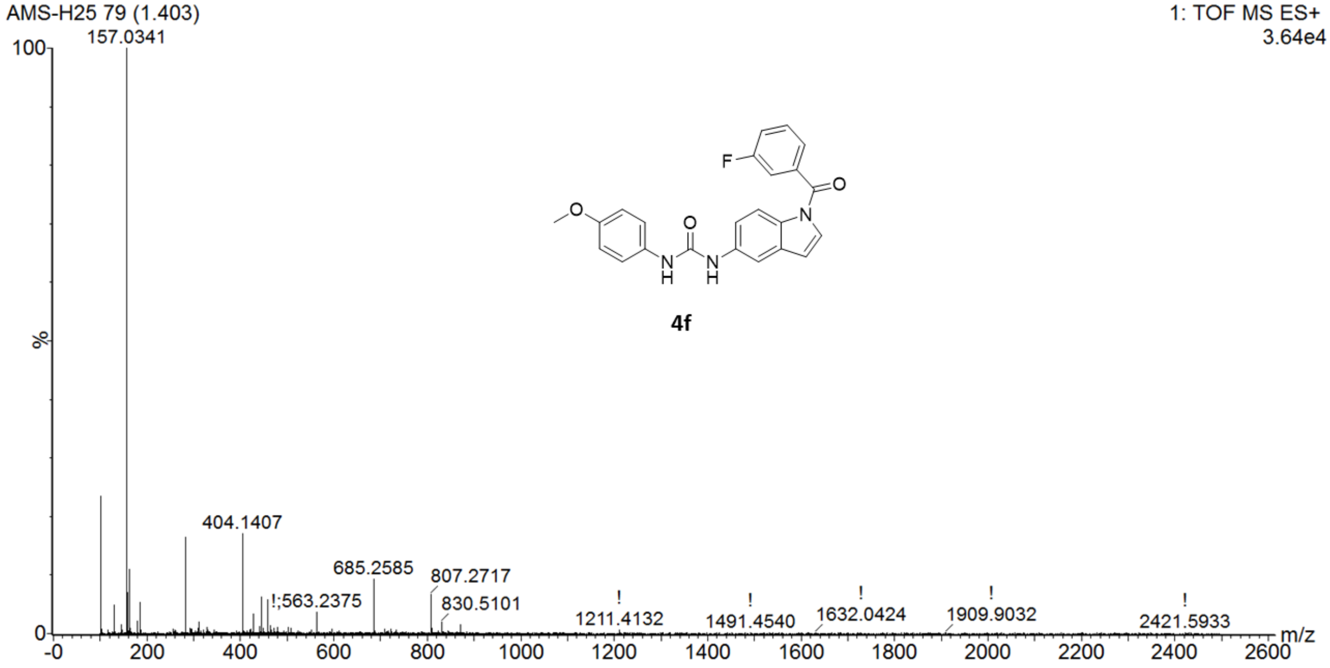
AMS-H25 79 (1.403)
1: TOF MS ES+
3.64e4
157.0341
404.1407
685.2585
807.2717
!;563.2375
830.5101
1211.4132
1491.4540
1632.0424
1909.9032
2421.5933
%
m/z
4f

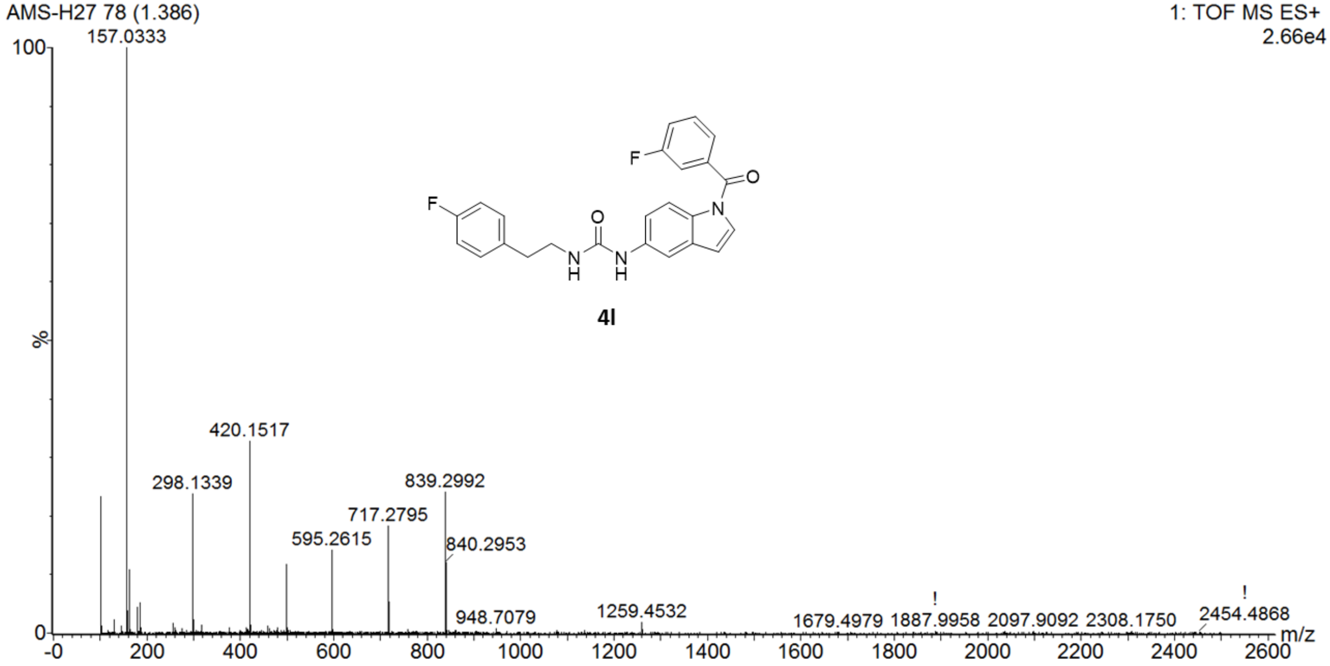
AMS-H27 78 (1.386)
1: TOF MS ES+
2.66e4
157.0333
420.1517
298.1339
839.2992
717.2795
595.2615
840.2953
948.7079
1259.4532
1679.4979
1887.9958
2097.9092
2308.1750
2454.4868
%
m/z
4l

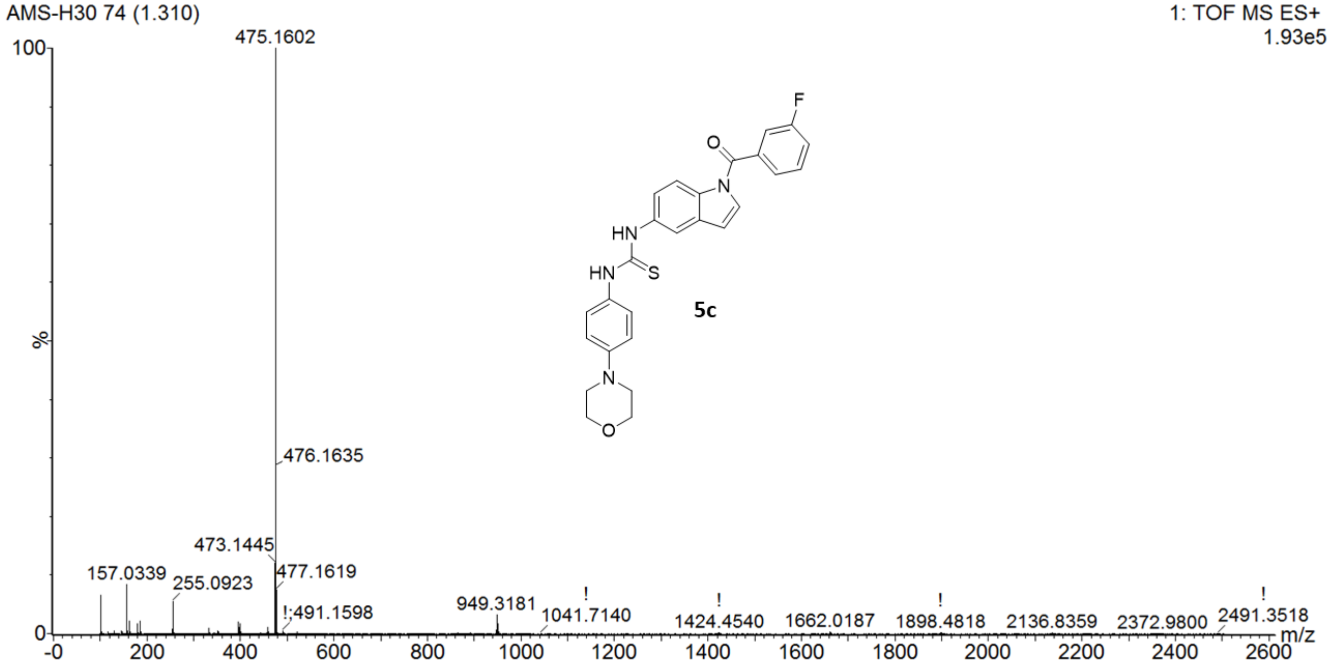
AMS-H30 74 (1.310)
1: TOF MS ES+
1.93e5
475.1602
476.1635
473.1445
477.1619
157.0339
255.0923
!;491.1598
949.3181
1041.7140
1424.4540
1662.0187
1898.4818
2136.8359
2372.9800
2491.3518
%
m/z
5c

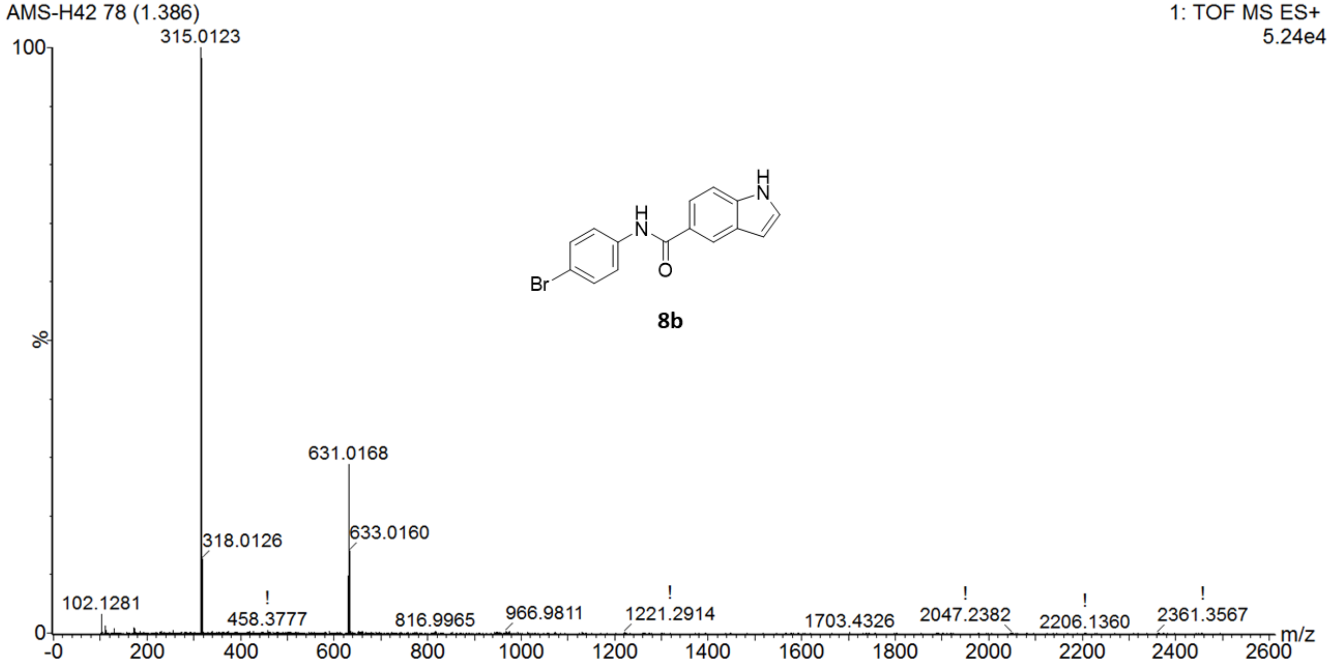
AMS-H42 78 (1.386)
1: TOF MS ES+
5.24e4
315.0123
631.0168
318.0126
633.0160
102.1281
458.3777
816.9965
966.9811
1221.2914
1703.4326
2047.2382
2206.1360
2361.3567
%
m/z
8b

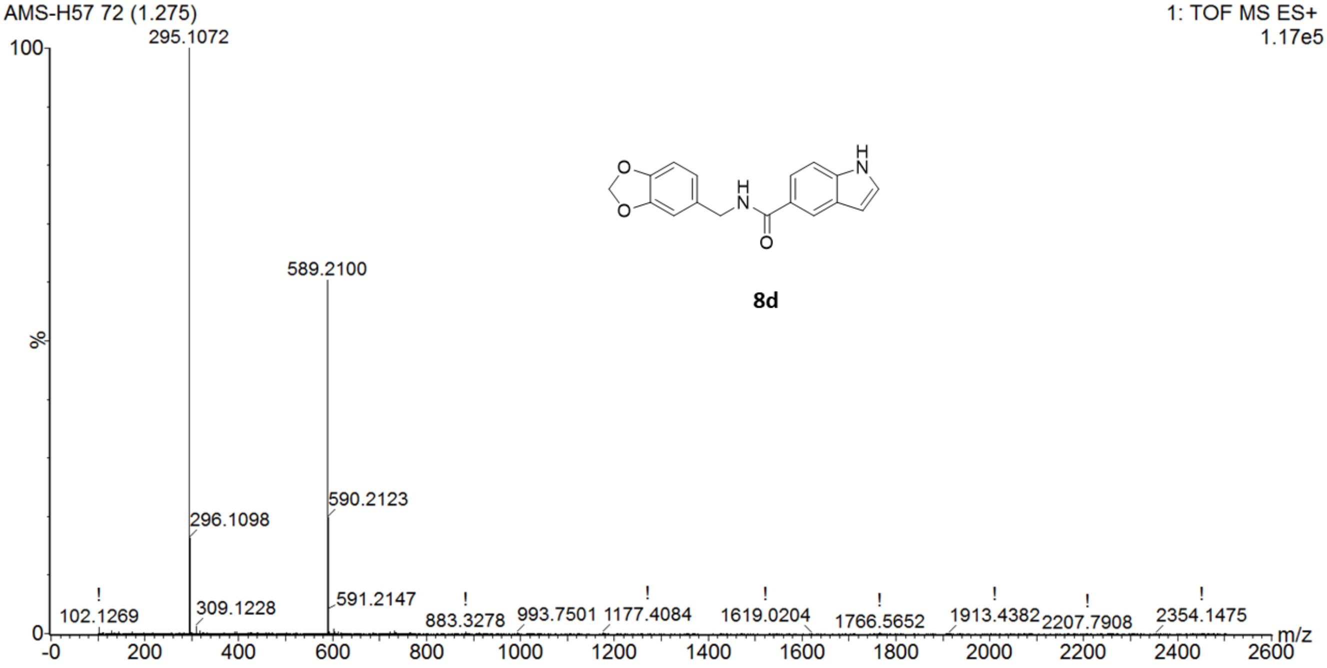
AMS-H57 72 (1.275)
1: TOF MS ES+
1.17e5
295.1072
589.2100
590.2123
296.1098
591.2147
309.1228
102.1269
883.3278
993.7501
1177.4084
1619.0204
1766.5652
1913.4382
2207.7908
2354.1475
%
m/z
8d

4. Molecular modelling supplementary material

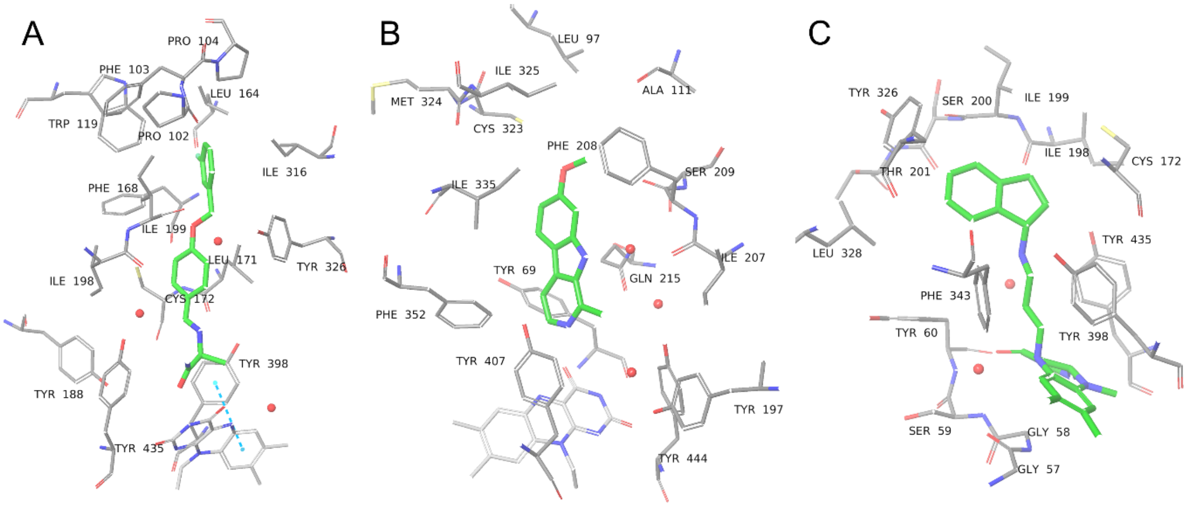

Figure S1. Binding modes of reference reversible inhibitors (A) safinamide bound to MAO-B (PDB ID: 2V5Z), (B) harmine bound to MAO-A (PDB ID: 2Z5X) and covalent irreversible inhibitor (C) rasagiline bound to MAO-B (PDB ID: 1S2Y). Ligands are presented as green thick sticks, pocket atoms – gray thin sticks and crucial water molecules - red spheres. Hydrogen atoms omitted for clarity.
